# Exploring Mesoionic Imine‐Carbodiimide (MII‐CDI) Adducts: 1,3 H‐Shift, N(I) Compounds and Guanidinate‐Type Ligands

**DOI:** 10.1002/anie.202502097

**Published:** 2025-07-16

**Authors:** Alok Mahata, Richard Rudolf, Robert R. M. Walter, Nicolás. I. Neuman, Biprajit Sarkar

**Affiliations:** ^1^ Institut für Anorganische Chemie Universität Stuttgart Pfaffenwaldring 55 70569 Stuttgart Germany; ^2^ Institut für Chemie und Biochemie, Anorganische Chemie Freie Universität Berlin Fabeckstraße 34–36 14195 Berlin Germany; ^3^ Instituto de Desarrollo Tecnológico para la Industria Química, Colectora Ruta Nacional 168 Km 0, Paraje El Pozo (S3000ZAA) Santa Fe Argentina

**Keywords:** Acyclic amino carbenes, Carbodiimide, Guanidinate ligands, Mesoionic imines, N(I) compounds

## Abstract

In this study, we report our recent findings on the synthesis and reactivity of a novel 1,2,3‐triazolin‐5‐imine‐type mesoionic imine‐carbodiimide (**MII‐CDI**) adduct. Unlike reported **NHC‐CDI** adducts, formed by the reactions of *N*‐heterocyclic carbene (**NHC**) with **CDI**, these zwitterionic compounds undergo a spontaneous 1,3‐hydrogen shift (1,3‐H shift), resulting in guanidine‐type compounds. The mechanism of this 1,3‐H shift has been investigated through quantum chemical calculations. The **MII‐CDI** adduct serves as a valuable synthon for the synthesis of mesoionic carbene‐acyclic diamino carbene (MIC‐ADC)‐based nitreone (**N(I)**) compounds. We have conducted a detailed investigation into the electronic properties, chemical reactivity, and electrochemical behavior of these nitreone (**N(I)**) compounds. Additionally, the potential of these **MII‐CDI** adducts as guanidinate ligands is explored. Our investigations here display the distinct reactivities of **MII** in contrast to their *N*‐heterocyclic imine (**NHI**) congeners.

## Graphical abstract

We demonstrate the unique reactivity of a mesoionic imine with bis‐aryl carbodiimides, forming an MII‐CDI adduct at room temperature via a spontaneous 1,3‐H shift. These MII‐CDI adducts serve as important synthons for mesoionic carbene‐acyclic diamino carbene‐stabilized nitreones (I) and guanidinate‐type ligands.

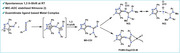

## Introduction

**Figure 1:**
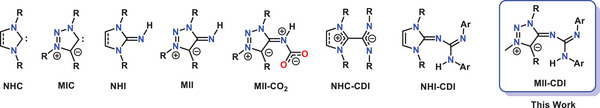
Chemical structures of *N*‐heterocyclic compounds.

*N*‐Heterocyclic carbenes (**NHCs**) are an important class of reactive organic molecules used as ligands and organocatalysts (Figure 1).^[^ ^1^ ^]^ Triazolylidene‐based mesoionic carbenes (**MICs**) are a comparatively new class of carbenes that have been popularized in recent years.^[^ ^2^, ^3^, ^4^, ^5^ ^]^ Just like NHCs, *N*‐heterocyclic imines (**NHIs**) are another significant class of ligands that are derived from the NHC scaffold with a highly polarized exocyclic C─N bond (Figure 1).^[^ ^6^ ^]^ Due to their high electron‐donating ability, NHIs have found numerous applications in stabilizing various low‐valent main group and transition metal compounds.^[^ ^7^, ^8^ ^]^ Since the first report of NHIs by Kuhn and coworkers in 1995,^[^ ^9^ ^]^ several other types of imines based on different carbene scaffolds and their reactivities have been reported by the groups of Tamm,^[^ ^10^ ^]^ Inoue,^[^ ^11^ ^]^ Braunschweig,^[^ ^12^ ^]^ and others.^[^ ^13^, ^14^, ^15^ ^]^ Among these, the 1,2,3‐triazolin‐5‐imine‐type mesoionic imine (**MII**) based on a MIC platform is one of the newest members, recently reported by us and others.^[^ ^16^, ^17^, ^18^, ^19^, ^20^, ^21^, ^22^ ^]^ Just like NHCs, NHIs as well as MIIs can react with carbon dioxide to form zwitterionic MII‐CO_2_ adduct (Figure 1).^[^ ^1^, ^15^, ^16^ ^]^

Another intriguing reactivity of NHCs involves their reaction with carbodiimides (**CDIs**) to form a special type of zwitterionic adduct (**NHC‐CDI**), which finds applications as ligands, junctions in supramolecular polymers, and stabilizers for radical cations (Figure 1).^[^ ^23^, ^24^, ^25^, ^26^ ^]^ Although carbon dioxide and carbodiimides are isostructural and show similar reactivity with NHCs, a direct reaction between any kind of NHI and CDI has not been reported in the literature, to the best of our knowledge.

In this study, we tested the reactivity of 1,2,3‐triazolin‐5‐imine‐type mesoionic imine (**MII**) with carbodiimides. It was found that MII can react with aryl‐CDIs at room temperature to form the corresponding adduct. However, this zwitterionic adduct undergoes an instantaneous 1,3 H‐shift to form a guanidine‐type compound (**MII‐CDI**) (Figure 1). To the best of our knowledge, such a 1,3 H‐shift in a reaction between carbodiimides and imines has not been previously reported in the literature. While harsh reaction conditions or metal‐catalyzed guanylation reactions of amines involving a 1,3 H‐shift are well‐documented,^[^ ^27^, ^28^, ^29^ ^]^ this spontaneous shift at room temperature without the addition of any additives in the context of MII‐CDI adducts is unprecedented. A similar type of adduct, **NHI‐CDI**, with an NHI backbone has also been reported in the literature (Figure 1).^[^ ^30^ ^]^ In that case, deprotonation of the corresponding NHI by *n*‐BuLi was used to activate it for the reaction with carbodiimide, and afterward, H₂O was used to reprotonate the corresponding lithiated NHI‐CDI compound.^[^ ^30^ ^]^

**Figure 2:**
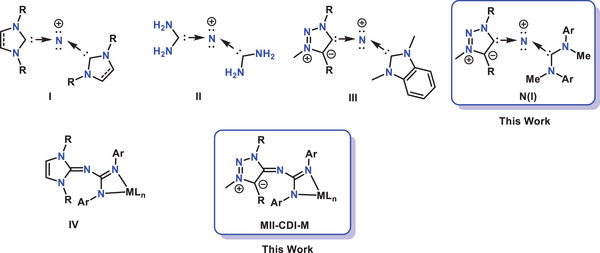
Chemical structures of **I–IV**, **N(I)**, and **MII‐CDI‐M**.

Along with synthesis, in this report, we delve into two distinct reactivities of MII‐CDIs. Firstly, we explore their potential as synthons for the synthesis of monocationic nitrogen(I) (**N(I)**) compounds. With the electronic structure (L → N ← L′)^+^, such a compound carries two lone pairs of electrons at the central nitrogen atom and can therefore be regarded as divalent **N(I)** compounds (Figure 2).^[^ ^31^ ^]^ Since nitrogen(I) is isoelectronic to carbon(0)‐species, the donor‐stabilized **N(I)** compounds are known as nitreones.^[^ ^32^ ^]^ These **N(I)** compounds have also gained considerable attention due to their medicinal importance as well as catalytic activity in phase transfer reactions.^[^ ^33^, ^34^ ^]^ Alongside phosphines and silylenes, several carbene‐based nitreones are reported in the literature (Figure 2, Compounds **I**–**III**).^[^ ^35^, ^36^, ^37^, ^38^, ^39^, ^40^ ^]^ The debate on the valid representation of nitreones remains active in the scientific community with two borderline considerations: Display of dative bonds (more arrows) is supported by Frenking, while Krossing proposed the representation according to Lewis with “classical” covalent bonds and less arrows.^[^ ^41^, ^42^, ^43^ ^]^

Second, we investigate the deprotonation of the **MII‐CDI** adduct and the potential of the resulting compound as a guanidinate‐type ligand. Guanidinate ligands, recognized for their Y‐shaped monoanionic structure, are adept at stabilizing a broad spectrum of mononuclear and polynuclear complexes.^[^ ^44^, ^45^, ^46^ ^]^ Their steric and electronic versatility, enhanced by amino or imino substituents on the central carbon atom, surpasses that of related formamidinate and amidinate ligands.^[^ ^44^, ^45^, ^46^ ^]^ Although several reports on guanidinate‐type ligands exist, the report by Fortier and coworkers on an imidazolin‐2‐iminato‐based guanidinate ligand (**NHI‐CDI**) and its corresponding Fe‐complexes is the only example of an NHI‐based guanidinate ligand reported in the literature (Figure 2, Compound **IV**).^[^ ^30^ ^]^

Through this research, we disclose the synthesis of a formal mesoionic carbene‐acyclic diamino carbene (MIC‐ADC)‐based nitreone compound (**N(I)**) derived from **MII‐CDI** and examine its (electro‐) chemical reactivities. Additionally, we also explored the reactivity of the deprotonated **MII‐CDI** adduct as a guanidinate type of ligand.

## Results and Discussion

### Synthesis and Characterization of MII‐CDI Adducts

**Scheme 1:**
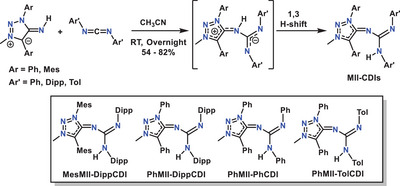
Synthesis of **MII‐CDIs** (Mes = 2,4,6‐Me_3_C_6_H_2_, Dipp = 2,6‐*i*Pr_2_C_6_H_3_, and Tol = 4‐Me‐C_6_H_4_).

**Figure 3:**
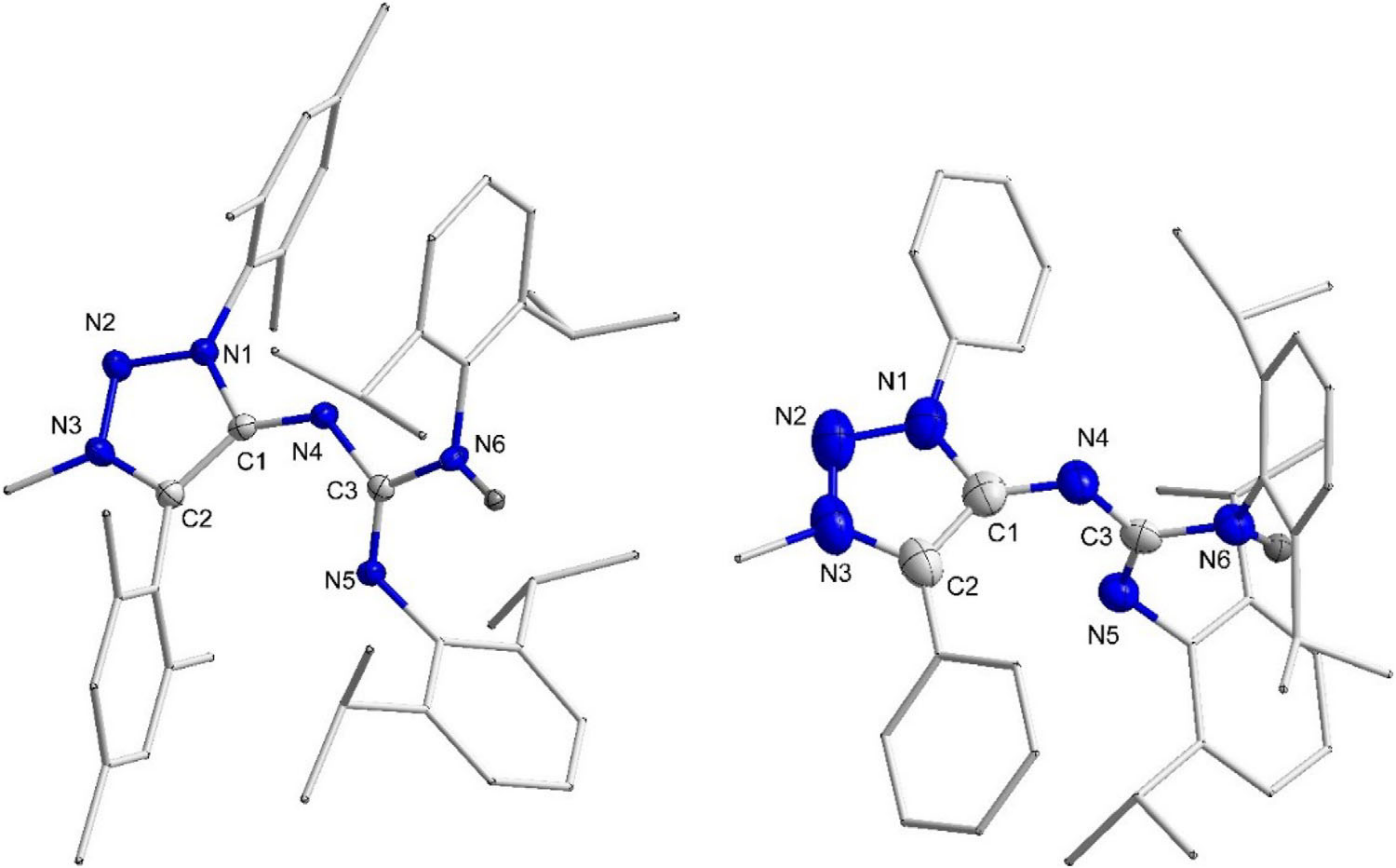
Molecular structure of **MesMII‐DippCDI** (left) and **PhMII‐DippCDI** (right) with thermal ellipsoids at the 50% probability level. CH_3_CN in the case of **PhMII‐DippCDI**, and all H atoms except N6─H are omitted and some fragments are displayed in wireframe representation for clarity reasons. Selected bond lengths (Å) and bond angle (°) **MesMII‐DippCDI**: N1─C1 1.396(1); C1─C2 1.415(2); C1─N4 1.311(2); N4─C3 1.365(1); C3─N5 1.293(2); C3─N6 1.388(2); C1─N4─C3 122.7(1). **PhMII‐DippCDI**: N1─C1 1.384(2); C1─C2 1.401(3); C1─N4 1.331(2); N4─C3 1.342(2); C3─N5 1.305(2); C3─N6 1.381(2); C1─N4─C3 121.4(1).

The synthesis of the mesoionic imines (MIIs) was performed following previously reported literature methods.^[^ ^16^ ^]^ Stirring MIIs overnight at room temperature with aryl‐carbodiimides resulted in the formation of the corresponding **MII‐CDI** adducts after a spontaneous 1,3‐hydrogen shift from the zwitterionic intermediate (Scheme 1). Density functional theory (DFT) calculations of the barrier for this reaction step for the case of **PhMII‐DippCDI** (Figure S41 and Table S12), performed using the NEB procedure in Orca,^[^ ^47^, ^48^ ^]^ show an activation energy of 104.8 kJ mol^−1^, with a TS where the H atom is nearly equidistant to both N atoms. The product is ∼55 kJ mol^−1^ more stable than the intermediate in Scheme 1. This reaction supports the amido‐type reactivity of mesoionic imines. All compounds were characterized by ^1^H, ^13^C NMR spectroscopy, elemental analysis as well as high‐resolution mass spectrometry. ^1^H NMR spectra of the 2,6‐diisopropylphenyl (Dipp)‐substituted adducts show characteristic signals of the N─H moiety at 4.34 and 4.68 ppm for **MesMII‐DippCDI** and **PhMII‐DippCDI**, respectively (Figures S1 and S3). However, the signal was missing in case of the substrates with the less sterically bulky phenyl (**PhMII‐PhCDI**) and tolyl (**PhMII‐TolCDI**) substituents (Figures S5 and S7). This behavior is presumably induced by *E*–*Z* isomerization of guanidine‐type compounds.^[^ ^49^, ^50^ ^]^ The more bulky Dipp substituents prefer the Z configuration to minimize steric repulsion with the MII backbone. But in the case of the less bulky phenyl or tolyl substituents, *E*–*Z* isomerization occurs at room temperature and hence the N─H proton engages in tautomerization with the two amidinate N‐atoms. By variable temperature ^1^H NMR spectroscopy conducted for **PhMII‐TolCDI**, we could show that different isomers exist at lower temperatures as apparent by individual signals assigned to the N─H moiety (Figure S9). The compounds were further characterized by single crystal XRD analysis in case of **MesMII‐DippCDI** and **PhMII‐DippCDI** (Figure 3).^[^ ^51^ ^]^ Both compounds crystallize in the triclinic P‐1 space group, **PhMII‐DippCDI** was co‐crystallized with CH_3_CN (Figure S29). As expected, the C3─N5 bonds were found to be much shorter than the C3─N6 bonds but more elongated than in the corresponding carbodiimide (1.213(2)Å and 1.221(2)Å, respectively).^[^ ^46^ ^]^ The C1─N4 bond (Table 1) in both **MesMII‐DippCDI** and **PhMII‐DippCDI** is elongated compared to the corresponding MIIs (1.300(3)Å for **MesMII** and 1.296(2)Å for **PhMII**).^[^ ^16^ ^]^ The N4─C3 bonds are slightly longer than the C1─N4 bonds (Figure 3 and Table 1), and both the bond lengths are shorter compared to a typical C─N single bond, further confirming the guanidine‐type nature of these adducts.

**Table 1:** Selected bond lengths (Å) and angles (°).

Bond/angle	**Dipp‐N(I)‐HOTf** (Å)/(°)	**Dipp‐N(I)** (Å)/(°)	**PhMII‐DippCDI** (Å)/(°)	**MIC‐N(I)‐NHC** (NT2) (Å)/(°)^[^ ^54^ ^]^
C1─N4	1.397(2)	1.341(3)	1.331(2)	1.332(4)
N4─C3	1.366(6)	1.309(3)	1.342(2)	1.335(4)
N1─C1	1.366(3)	1.372(4)	1.384(2)	1.393(3)
C1─C2	1.365(3)	1.398(4)	1.401(3)	1.397(4)
C3─N5	1.343(2)	1.370(3)	1.305(2)	1.351(4)
C3─N6	1.338(3)	1.353(3)	1.381(2)	1.356(4)
C1─N4─C3	132.9(2)	134.0(2)	121.4(1)	118.9(2)

To extend this study to alkyl‐substituted CDI'4s, we performed the reaction of **PhMII** with *N*,*N*‐diisopropyl carbodiimide (**DIC** or **iPrCDI**). Unfortunately, under the same conditions as applied for the reaction with the aryl‐substituted CDIs no reaction with the corresponding **MII** with **iPrCDI** at room temperature was observed. In contrast to the aryl‐substituted congeners the reactivity of alky‐CDIs is decreased as the alkyl‐substituents increase the electron density on the electrophilic C‐atom.^[^ ^52^ ^]^ However, the **PhMII‐iPrCDI** adduct could be formed by heating the reaction mixture to 65 °C for 3 days. In this case an excess of (more than 3 equiv) of **iPrCDI** was necessary to increase the conversion to preparative reasonable quantities. As the separation of the desired product from the unconverted starting CDI proved to be difficult, isolation of the desired product in completely pure form was not possible. However, the formation of the product could be confirmed by HRMS and NMR spectroscopy (Figure S10). Current work in our laboratories is focussed at solving this problem. In order to understand the different outcomes of this reaction, we performed preliminary calculations of the energy profiles for the reaction of **MII** with **iPrCDI** and **DippCDI** (Figures S52 and S53). The barrier for the first case was found to be approximately 70 kJ mol^−1^, with a local maximum displaying an N─C distance of 1.9 Å. For the second case, the barrier was considerably lower (45 kJ mol^−1^), with an N─C distance of 1.9 Å and a pre‐barrier local minimum with a distance of 2.8 Å. The differences in energies appear to be due to stabilizing effects of π‐stacking interactions between the Ph groups in the **MII** and the Dipp groups in **DippCDI**. Despite the preliminary character of these calculations, the energy profiles offer insight on the difference in reactivity.

### Synthesis, Characterization, and Properties of N(I) Compounds

**Scheme 2:**
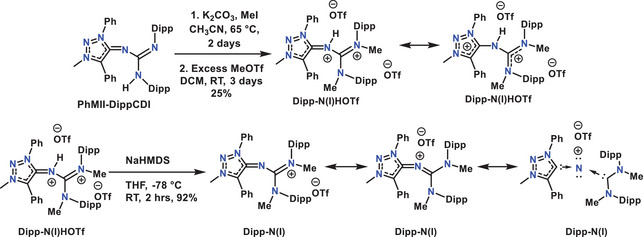
Synthesis of **Dipp‐N(I)HOTf** and **Dipp‐N(I)**.

To explore the further reactivity of these adducts, we have chosen **PhMII‐DippCDI** as a template initially. Two successive methylations of the adduct was expected to produce the desired **Dipp‐N(I)** compound (Scheme 2). However, when 1 equiv of MeOTf was used in the second step of the reaction, we consistently ended up with a mixture of products. Therefore, an excess of MeOTf was used, but instead of the formation of the expected **Dipp‐N(I)** compound, further protonation occurred, and the formation of **Dipp‐N(I)HOTf** was observed. A similar type of reactivity has been reported in the literature for bis‐NHC stabilized nitreone (I) compounds.^[^ ^53^ ^]^ Gratifyingly, deprotonation of **Dipp‐N(I)HOTf** with NaHMDS afforded the expected MIC‐ADC‐based **Dipp‐N(I)** compound (Scheme 2). Both the compounds **Dipp‐N(I)** and **Dipp‐N(I)HOTf** were characterized by ^1^H, ^13^C‐NMR spectroscopy, elemental analysis as well as high‐resolution mass spectrometry. Due to restricted rotation, several rotational isomers are present at room temperature. Thorough interpretation of the NMR spectra at room temperature therefore proved to be difficult. ^1^H‐NMR spectra of **Dipp‐N(I)** at variable temperatures were recorded which confirms the presence of rotational isomers (Figure S14). Despite a doubly cationic charge, the signal assigned to the N─H proton of **Dipp‐N(I)HOTf** in the ^1^H NMR spectrum is highly shielded (4.4 ppm, Figure S11), suggesting the presence of electron density around the nitreone nitrogen.

**Figure 4:**
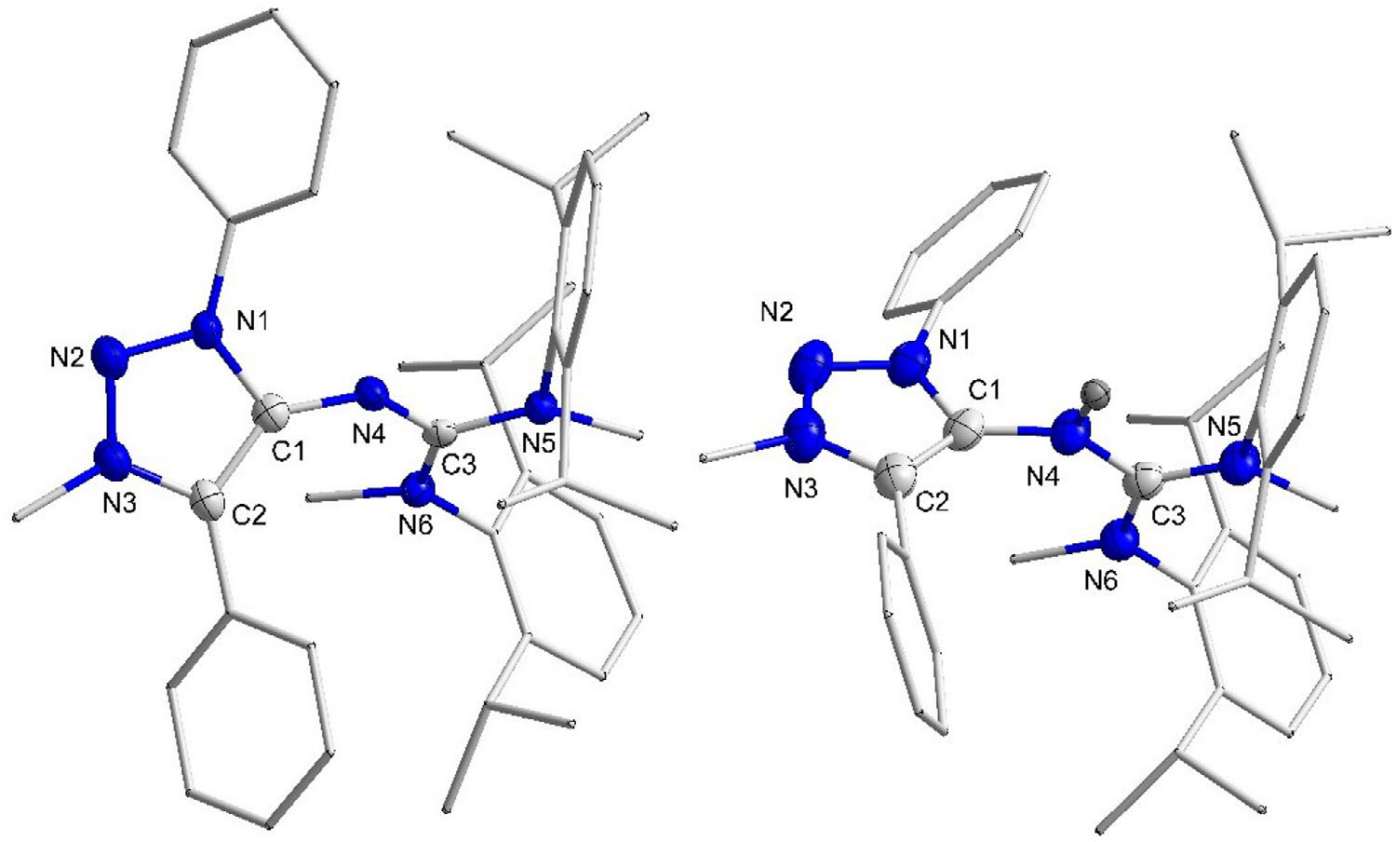
Molecular structure of **Dipp‐N(I)** (left) and **Dipp‐N(I)HOTf** (right) with thermal ellipsoids at the 50% probability level. Triflate anions and all H atoms except N4─H (in case of **Dipp‐N(I)HOTf**) are omitted, and some fragments are displayed in wireframe representation for clarity reasons. Selected bond lengths (Å) and bond angle (°) for **Dipp‐N(I)**: N1─C1 1.372(4); C1─C2 1.398(4); C1─N4 1.341(3); N4─C3 1.309(3); C3─N5 1.370(3); C3─N6 1.353(3); C1─N4─C3 134.0(2). **Dipp‐N(I)HOTf**: N1─C1 1.366(3); C1─C2 1.365(3); C1─N4 1.397(2); N4─C3 1.366(6); C3─N5 1.343(2); C3─N6 1.338(3); C1─N4─C3 132.9(2).

Single crystals of both **Dipp‐N(I)** and **Dipp‐N(I)HOTf**, suitable for XRD analysis, were obtained after the slow diffusion of Et₂O into a saturated solution of the compound in DCM. In the following section, the structural properties of these compounds are discussed (Figure 4). While **Dipp‐N(I)** crystallizes in the monoclinic P2₁/c space group, **Dipp‐N(I)HOTf** crystallizes in the triclinic P‐1 space group.^[^ ^51^ ^]^ The molecular structure in the crystal of **Dipp‐N(I)** (Figure 4) reveals a bent geometry across the C1─N4─C3 with an angle of 134.0(2)°. The C1─N4 and N4─C3 bond lengths are 1.341(3) and 1.309(3) Å, respectively, which are intermediate between typical C─N single bonds and C═N double bonds, as expected for nitreones. The angle between the plane containing N1─C1─C2─N4 and that containing N4─C3─N5─N6 is 55.6°, suggesting there is no conjugation between the two carbene units via N4 and also indicating that there is no heteroallene character, as both units are not orthogonal to each other.

In the case of **Dipp‐N(I)HOTf**, the C1─N4 and N4─C3 bond lengths are 1.397(2) and 1.366(6) Å, respectively, which are significantly elongated compared to those in **Dipp‐N(I)**. Additionally, the C1─C2, C3─N5, and C3─N6 bond lengths are 1.365(3), 1.343(2), and 1.338(3) Å, respectively, which are considerably shorter than those in **Dipp‐N(I)**, which are 1.398(4), 1.370(3), and 1.353(3) Å, respectively. These observations imply that the two cationic charges are more localized on the two carbene units, corroborating the findings from the ¹H NMR spectra of **Dipp‐N(I)HOTf**, and giving credence to the description shown on the top right of Scheme 2. All bond parameters have been summarized and compared with the reported data for the nitreone MIC‐N(I)‐NHC^[^ ^54^ ^]^ in Table 1.

**Scheme 3:**
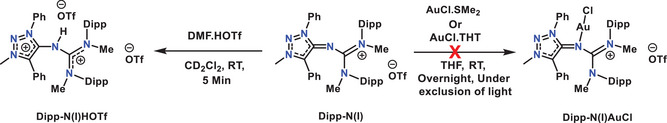
Reactivities of **Dipp‐N(I)** toward protonation and gold chloride.

Due to the presence of a cationic charge in the molecules, nitreones (I) have much less donor capability compared to carbon(0).^[^ ^35^, ^36^, ^38^, ^39^, ^40^ ^]^ Here, we tested the reactivity of **Dipp‐N(I)** toward protonation (using DMF·HOTf) and gold chloride (using AuCl·SMe_2_ and AuCl·THT) (Scheme 3). In the first case, **Dipp‐N(I)** immediately reacted with DMF·HOTf to form the corresponding **Dipp‐N(I)HOTf**. However, it was not possible to form a complex during the reaction with either AuCl·SMe_2_ or AuCl·THT. In both cases, deposition of elemental gold was observed, likely pointing to a redox reaction involving the **Dipp‐N(I)** compound. Following the reaction of **Dipp‐N(I)** with AlCl_3_ via ^1^H‐NMR spectroscopy suggested the formation of the desired complex as well resolved peaks and a downfield shift of the signals assigned to the methyl and aryl protons compared to the starting compound **Dipp‐N(I)** was detected (Figure S23). Unfortunately, until now the isolation of the target compound was unsuccessful and further structural characterization is pending.

**Scheme 4:**
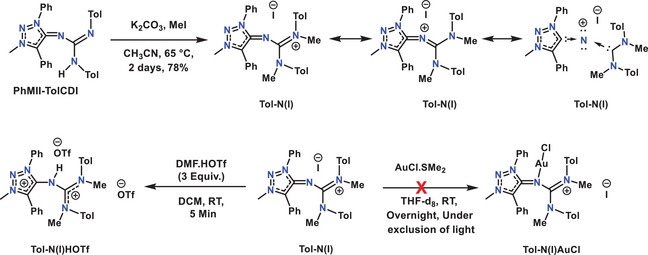
Synthesis of **Tol‐N(I)** and **Tol‐N(I)HOTf**.

To further gauge if the observed reactivities of the nitreone compounds are dominated by steric or electronic effects, we synthesized the compound **Tol‐N(I)** from the corresponding **PhMII‐TolCDI** adduct with the less bulky tolyl‐substituents. In this case, the reaction went as expected. Two successive methylation of the **PhMII‐TolCDI** adduct using MeI in presence of K_2_CO_3_ in CH_3_CN yielded **Tol‐N(I)** in good yields after heating at 65 °C for 3 days (Scheme 4). The product was characterized by ^1^H, ^13^C NMR spectroscopy, elemental analysis as well as high‐resolution mass spectrometry. In contrast to **Dipp‐N(I)** with the bulky dipp‐substituents ^1^H NMR spectroscopy of **Tol‐N(I)** shows well resolved signals. All the Ph‐protons come as multiplets between 7.59 and 7.83 ppm, aryl protons from tolyl group as two doublets at 6.96 and 6.68 ppm. CH_3_ protons appear at 4.15, 2.68 and 2.23 ppm for triazole N‐C*H*_3_, amidine N‐C*H*_3_, and tolyl‐C*H*_3_, respectively (Figure S16). The reactivity tests of **Tol‐N(I)** toward DMF·HOTf and AuCl·SMe_2_ showed similar results as observed for **Dipp‐N(I)**. **Tol‐N(I)HOTf** could be synthesized by the reaction of **Tol‐N(I)** with DMF·HOTf (Scheme 4). The ^1^H NMR spectra of **Tol‐N(I)HOTf** also shows well resolved signals. Compared to the signals of **Tol‐N(I)**, the signals of the obtained compound in the ^1^H NMR spectrum are shifted to downfield which suggests decrease of electron density and hence formation of another cationic charge in the molecule. The reaction of **Tol‐N(I)** with the Au(I)‐precursor unfortunately did not lead to the formation of the desired Au‐complex. This suggests that the reactivity of the herein reported nitreone compounds is the result of the electronic structure with no decisive influence of the sterics. As observed with **Dipp‐N(I)**, the reaction of **Tol‐N(I)** with AlCl_3_ resulted in the formation of a selective product according to ^1^H NMR spectroscopy. The formed compound shows a similar downfield shift of all the methyl and aryl proton of the tolyl groups (Figure S24). This again suggests decrease of electron density from the molecule and hence formation of **Tol‐N(I)AlCl_3_**. Isolation and structural characterization of this compound is pending.

**Figure 5:**
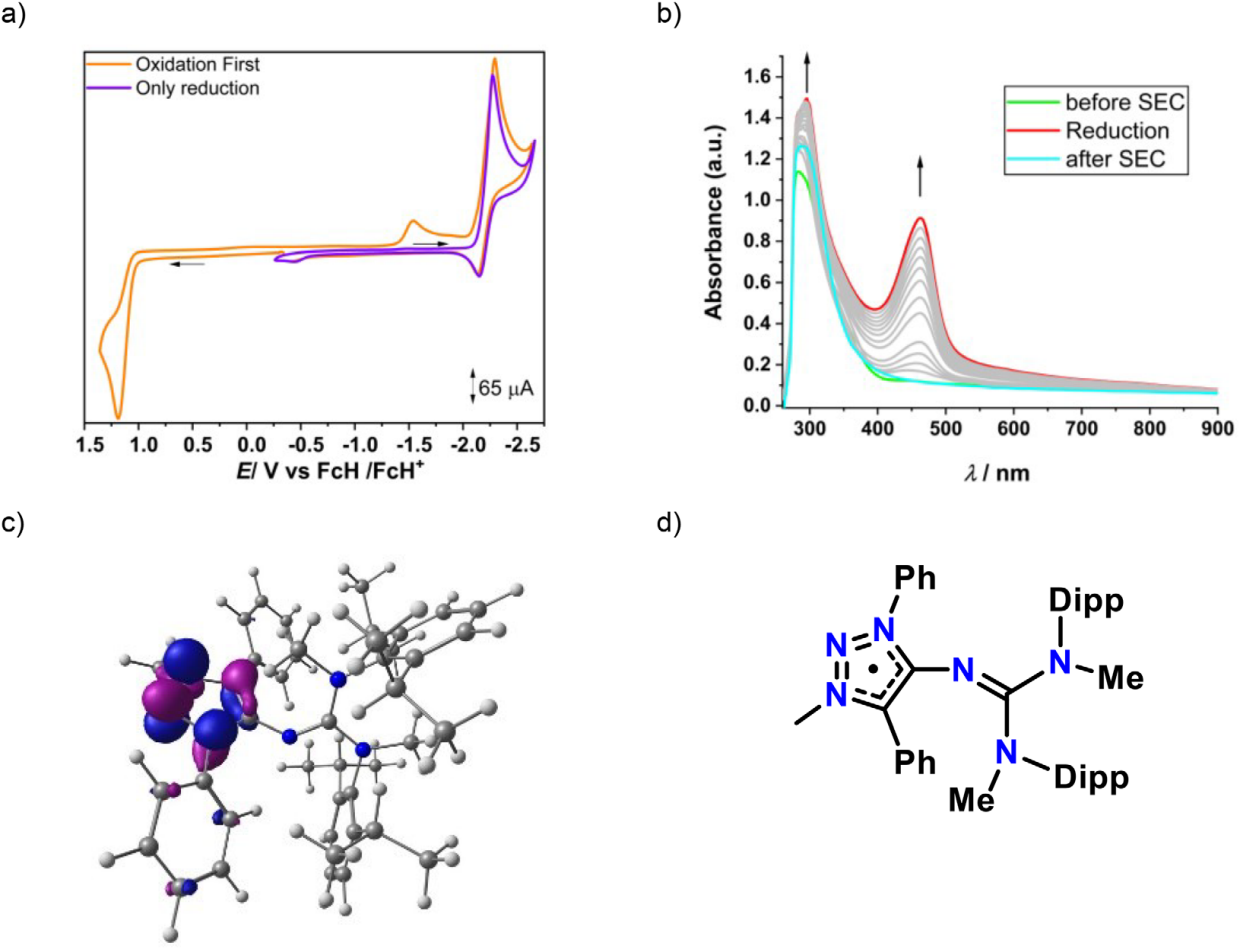
a) CV of **Dipp‐N(I)** in *o*‐DFB/0.1 MBu_4_NPF_6_ was measured at a GC working electrode at 100 mVs^−1^. b) Changes in the UV/VIS/NIR spectra of **Dipp‐N(I)** in *o*‐DFB/0.1 MBu_4_NPF_6_ during the reduction with an Au working electrode. c) SOMO of the reduced species of **Dipp‐N(I)** (isosurface 0.06). d) Expected radical species generated after one electron reduction of **Dipp‐N(I)**.

The cyclic voltammetry (CV) study of **Dipp‐N(I)** in *o*‐difluorobenzene, with NBu_4_PF_6_ as the supporting electrolyte, revealed one irreversible oxidation and one quasi‐reversible reduction (Figure 5). The oxidation step most likely involves the formation of a nitreone‐N‐based radical cation, which immediately undergoes hydrogen atom abstraction or other quenching processes under CV conditions. The quasi‐reversible reduction process appears to be primarily associated with the triazole ring. It is known in the literature that 1,2,3‐triazole‐based mesoionic backbone can stabilize a radical.^[^ ^55^, ^56^, ^57^ ^]^

To further investigate the nature of the reduced species, UV/Vis/NIR spectroelectrochemistry was performed, suggesting the formation of a new compound with *λ*_Max_ around 470 nm (Figure 5). DFT calculations suggest that the SOMO of the reduced species is predominantly located on the triazole ring (Figure 5), also the spin density calculations suggest the same (Figure S40). To acquire a better understanding of this reduced species, EPR spectroelectrochemistry also was performed at −10 °C. A signal for organic radical species could be seen during electrolysis (Figure S37), but it undergoes further transformations at that condition. To the best of our knowledge, this is the first thorough electrochemical and spectroelectrochemical characterization of a reduced nitreone species, which is a *formal* **N(0)** compound. The *formal* **N(0)** species is strongly stabilized by the triazoline substituent. However, chemical isolation of the reduced species did not work out. Even though a trapping reaction with known radical trapping reagent dimethyldisulfide (DMDS) during the reduction with KC_8_ did indicate the formation of an adduct (ESI‐MS evidence), we were unfortunately not able to isolate that adduct in a pure form.

**Figure 6:**
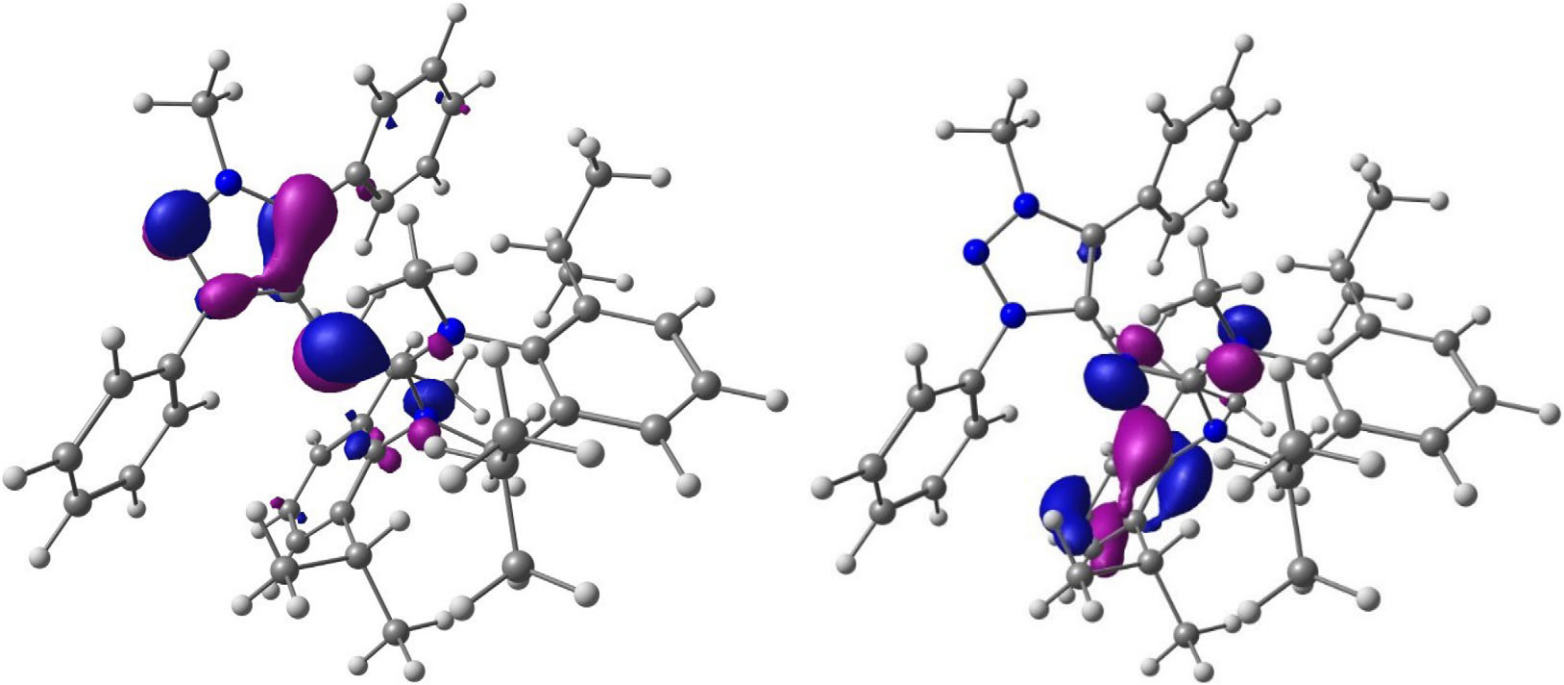
HOMO (left) and HOMO‐3 (right) of the **Dipp‐N(I)** (isosurface 0.06).

To gain more insight into the electronic structures of **Dipp‐N(I)** and **Dipp‐N(I)HOTf**, we performed DFT calculations using the PBE0 method with the def2TZVP basis set (details are provided in the Supporting Information). The electronic structures were examined through natural bond orbital (NBO) analysis, natural population analysis (NPA), Wiberg Bond Indices (WBI), and frontier molecular orbital calculations. The analysis of the frontier molecular orbitals indicated the presence of two lone pairs of electrons at the nitreone *N*. Specifically, the HOMO of **Dipp‐N(I)** corresponds to the π‐type lone pair, while the HOMO‒3 corresponds to the σ‐type lone pair (Figure 6). These findings are consistent with the recently reported MIC‐N(I)‐NHC complex^[^ ^54^ ^]^ and are summarized in Table 2. Moreover, a significant negative charge (−0.61) at the nitreone nitrogen of **Dipp‐N(I)HOTf**, along with a WBI value for C1─N4 and N4─C3 of approximately 1.0, further supports the hypothesis of the presence of electron density around the nitreone nitrogen in **Dipp‐N(I)HOTf**.

**Table 2:** Summary of computational calculations.

	NBO charge at N1	WBI (C1─N4)	WBI (C3─N4)	1st proton affinity^a)^	Me cation affinity^a)^	AuCl complexation energy^a)^	BH_3_ complexation energy^a)^
**Dipp‐N(I)‐HOTf**	−0.61	1.0	1.1	–	–	–	–
**Dipp‐N(I)**	−0.63	1.3	1.3	260.31	72.62	−38.06	−13.98
MIC‐N(I)‐NHC (NT2)^[^ ^54^ ^]^	−0.64	1.3	1.3	149.67	–	−30.89	−15.53

^a)^ Values given in kcal mol^−1^.

We also calculated the energy profiles for torsion around the C1─N4 and C3─N4 bonds (Figures S42 and S44) finding a maximum barrier of rotation of ∼30 kJ mol^−1^ (∼7.5 kcal mol^−1^) in both cases, suggesting a predominantly single bond character of the C‐N bonds (for amides, the barriers have been reported to be in the 15–20 kcal mol^−1^ range).^[^ ^58^ ^]^ Figures S43 and S45 show the superimposed initial and final structures from each scan. The final structures are higher in energy due to different orientations of the phenyl and diisopropylphenyl substituents. Furthermore, we performed electron localization function (ELF) analysis using the Multiwfn program.^[^ ^59^, ^60^ ^]^ The ELF was calculated and plotted as an isosurface (Figure S46). The shape of the ELF around the N(I) atom suggests the presence of occupied σ‐type p‐orbital and to a lesser extent a π‐type p‐orbital, located perpendicular to the C─N─C plane. Figures S47–S49 show heatmap plots of the intersections of the ELF function on different molecular planes obtained by selecting sets of 3 atoms. Further insight on the electronic properties of the N(I) center can be obtained from analyzing the bond angle energy profile for the central C─N─C motif. This energy profile, obtained from relaxed surface scans on **Dipp‐N(I)**, as well as a truncated form where the Dipp groups were replaced by Me groups, is shown in Figure S50. The full molecule presents a minimum energy angle of 135°, while the truncated form, one of 123°. The higher linearity of the bond in **Dipp‐N(I)**, compared to its truncated analog, suggests steric repulsion effects caused by the bulky Dipp substituents. An ideal N(I) center would tend to form a linear C─N─C bond due to the presence of two occupied lone pairs in the N, while a smaller bond angle suggests a higher nitrenium character.^[^ ^32^ ^]^ The C─N─C angle in **Dipp‐N(I)** is on the larger side compared to the systems studied in Ref. [32] Finally, we calculated the Fukui functions *f*_+_ and *f_−_*, corresponding to the difference between the electron densities of the reduced and starting species, and between that of the starting and oxidized species, respectively (Figure S51). The *f*_+_ function indicates that reduction is mostly localized on the triazole moiety, in agreement with the results shown in Figure 5c. On the other hand, *f_−_* suggests electron subtraction occurs at the N(I) site.

### Conversion of the MII‐CDI Adduct to Guanidinate Ligand

**Scheme 5:**
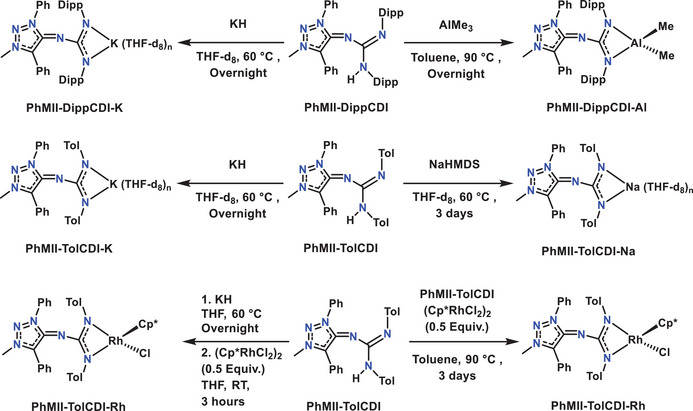
**MII‐CDI** adducts as precursor of guanidinate ligand (Cp* = C_5_(CH_3_)_5_ anion).

To show the second type of reactivity study of **MII‐CDIs**, we choose to form a guanidinate ligand‐based aluminum complex. This kind of complexes are known in the literature for their activity as a catalyst in ring opening polymerization and catalytic guanylation of carbodiimides.^[^ ^29^, ^61^, ^62^ ^]^ The reaction of the adduct **PhMII‐DippCDI** with AlMe_3_ resulted in the formation of the complex **PhMII‐DippCDI‐Al** (Scheme 5). Preparation of the corresponding alkali metal salts were conducted by deprotonation reaction of the **MII‐CDI** adducts with bases like KH or NaHMDS in a J Young NMR tube (Scheme 5). The product formation was followed by ^1^H NMR spectroscopy. Overnight heating at 60 °C was required to deprotonate **PhMII‐DippCDI** and **PhMII‐TolCDI** using KH in THF‐d_8_. During the reaction, the formation of a gas (H_2_) was observed and the color of the solution changed from yellow to light orange. The recorded ^1^H NMR spectra showed significant up‐field shift of all the aromatic protons which suggests formation of a negative charge in the molecule (Figures S25 and S26). Additionally, a characteristic peak at 4.55 ppm was observed which provided spectroscopical evidence for the formation of H_2_.^[^ ^63^ ^]^ Deprotonation reactions of **PhMII‐TolCDI** with NaHMDS resulted in similar results and the formation of the desired salt as indicated by ^1^H‐NMR‐spectroscopy (Figure S27). For complete conversion, the reaction mixture was heated at 60 °C for 3 days. These methods provided a simple access to guanidinate synthons in the form of alkali metal salts. We therefore decided to react 2 equiv of **PhMII‐TolCDI‐K** with (Cp*RhCl_2_)_2_ to form corresponding air stable rhodium complex. The formation of the product **PhMII‐TolCDI‐Rh** could be confirmed by ^1^H, ^13^C‐NMR spectroscopy as well as high‐resolution mass spectrometry. In the presence of a metal precursor like (Cp*RhCl_2_)_2_, **PhMII‐TolCDI** itself can act as a base to catch the HCl formed in the complex formation. Hence, the reaction of 4 equiv **PhMII‐TolCDI** with (Cp*RhCl_2_)_2_ also provided the same metal complex **PhMII‐TolCDI‐Rh** after heating at 90 °C in toluene for 3 days (Scheme 5).

**Figure 7:**
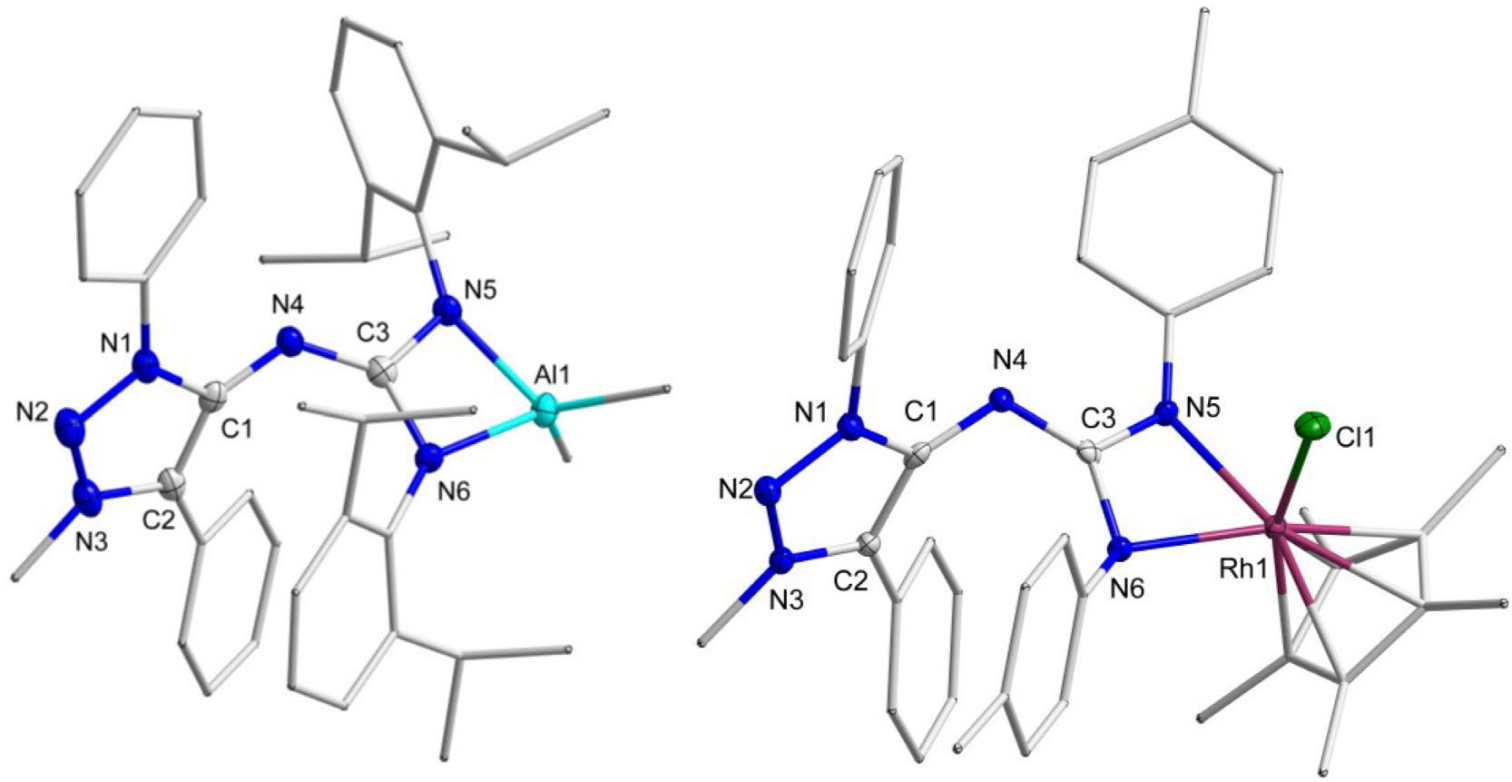
Molecular structure of **PhMII‐DippCDI‐Al** (left) and **PhMII‐DippCDI‐Rh** (right) with thermal ellipsoids at the 50% probability level. All H atoms are omitted, and some fragments are displayed in wireframe representation for clarity reasons. Selected bond lengths (Å) and bond angle (°) for **PhMII‐DippCDI‐Al**: N1─C1 1.387(2); C1─C2 1.411(2); C1─N4 1.334(2); N4─C3 1.329(2); C3─N5 1.361(2); C3─N6 1.367(2); N5─Al1 1.899(1); N6─Al1 1.952(1); C1─N4─C3 127.2(1); N5─C3─N6 107.8(1); N5─Al1─N6 69.81(5). **PhMII‐DippCDI‐Rh**: N1─C1 1.381(5); C1─C2 1.411(5); C1─N4 1.326(5); N4─C3 1.345(5); C3─N5 1.341(5); C3─N6 1.358(5); N5─Rh1 2.079(4); N6─Rh1 2.225(3); C1─N4─C3 118.1(3); N5─C3─N6 106.9(3); N5─Rh1─N6 62.3(1).

Both of the metal complexes, **PhMII‐DippCDI‐Al** and **PhMII‐TolCDI‐Rh**, were further characterized by single crystal XRD analysis (Figure 7).^[^ ^51^ ^]^ In the case of **PhMII‐DippCDI‐Al** single crystals suitable for XRD analysis were obtained from highly concentrated solutions of the complex in pentane. Slow diffusion of Et_2_O in a concentrated solution of **PhMII‐TolCDI‐Rh** in DCM yielded suitable single‐crystals of the corresponding Rh‐complex, which was co‐crystallized with DCM (Figure S33). Unlike the adducts, the molecular structures from crystal of both complexes reveal comparable bond length of the C3─N5 and C3─N6 bond, suggesting the delocalization of the negative charge over the N5─C3─N6 unit. This fact is expected for a guanidinate type of ligand‐based metal complex. The C1─N4 bond remains comparable with **PhMII‐DippCDI**, but shorter N4─C3 bond and wider C1─N4─C3 angle in case of **PhMII‐DippCDI‐Al** complex suggests more delocalization of electron density from the central nitrogen (N4) to the CDI unit (Figure 7).

## Conclusion

In conclusion, we have successfully synthesized a mesoionic imine‐carbodiimide (**MII‐CDI**) adduct from the corresponding **MII** and **CDI** following a spontaneous 1,3‐H shift, without the addition of any base. This reactivity highlights the distinct chemical behavior of mesoionic imines compared to their more conventional counterparts. We also synthesized nitreone compounds (**Dipp‐N(I)** and **Tol‐N(I)**), stabilized by a mesoionic 1,2,3‐triazolylidene carbene and an acyclic diamino carbene, using **PhMII‐DippCDI** and **PhMII‐TolCDI**, respectively, as the starting precursor. The electronic and geometric parameters of **Dipp‐N(I)**, obtained through DFT calculations and X‐ray crystallography, confirm the nitreone nature of the central nitrogen. We also studied the chemical reactivity of these nitreones toward protonation and complex formation reactions, as well as their electrochemical properties. The protonated version of **Dipp‐N(I)**, i.e., **Dipp‐N(I)HOTf**, was successfully isolated and structurally characterized. To the best of our knowledge, this is the first example of a crystal structure of a protonated nitreone compound supported by a mesoionic carbene. Sterically less bulky tolyl substituents, compared to Dipp substituents, can ease the synthesis. However, the resulting compounds show similar reactivities, which suggests that the electronic structure dominates the chemical reactivities of these compounds over sterics. We have also presented electrochemical and spectroelectrochemical data on a transient, *formally* **N(0)** compound formed by the one‐electron reduction of the nitreone species. **MII‐CDI** adducts could be used as precursors for the synthesis of guanidinate‐type metal complexes. In this regard, we have presented the preparation and structural characterization of both main group (**PhMII‐DippCDI‐Al**) and transition metal complex (**PhMII‐TolCDI‐Rh**) using **PhMII‐DippCDI** and **PhMII‐TolCDI**, respectively. Further investigations in both of these directions are currently ongoing in our laboratory.

## Conflict of Interests

The authors declare no conflict of interest.

## Supporting information



Supporting Information

Supporting Information

## Data Availability

The data that support the findings of this study are available in the Supporting Information of this article.

## References

[1] M. N. Hopkinson , C. Ritter , M. Schedler , F. Glorious , Nature 2014, 510, 485–496. 24965649 10.1038/nature13384

[2] G. Guisado‐Barrios , M. Soleilhavoup , G. Bertrand , Acc. Chem. Res. 2018, 51, 3236–3244. 30417642 10.1021/acs.accounts.8b00480

[3] Á. Vivancos , C. Segarra , M. Albrecht , Chem. Rev. 2018, 118, 9493–9586. 30014699 10.1021/acs.chemrev.8b00148

[4] D. Schweinfurth , L. Hettmanczyk , L. Suntrup , B. Sarkar , Z. Anorg. Allg. Chem. 2017, 643, 554–584.

[5] R. Maity , B. Sarkar , JACS Au 2022, 2, 22–57. 35098220 10.1021/jacsau.1c00338 PMC8790748

[6] A. Doddi , M. Peters , M. Tamm , Chem. Rev. 2019, 119, 6994–7112. 30983327 10.1021/acs.chemrev.8b00791

[7] T. Ochiai , D. Franz , S. Inoue , Chem. Soc. Rev. 2016, 45, 6327–6344. 27499203 10.1039/c6cs00163g

[8] X. Wu , M. Tamm , Coord. Chem. Rev. 2014, 260, 116–138.

[9] N. Kuhn , R. Fawzi , M. Steimann , J. Wiethoff , D. Bläser , R. Z. Boese , Z. Naturforsch. B 1995, 50, 1779–1784.

[10] M. Tamm , D. Petrovic , S. Randoll , S. Beer , T. Bannenberg , P. G. Jones , J. Grunenberg , Org. Biomol. Chem. 2007, 5, 523. 17252136 10.1039/b615418b

[11] D. Franz , E. Irran , S. Inoue , Angew. Chem. Int. Ed. 2014, 53, 14264–14268. 10.1002/anie.201407809 25323781

[12] J. T. Goettel , H. Gao , S. Dotzauer , H. Braunschweig , Chem. Eur. J. 2020, 26, 1136–1143. 31777982 10.1002/chem.201904715 PMC7027825

[13] L. F. B. Wilm , T. Eder , C. Mgck‐Lichtenfeld , P. Mehlmann , M. Wgnsche , F. Buß , F. Dielmann , Green Chem. 2019, 21, 640–648.

[14] L. F. B. Wilm , M. Das , D. Janssen‐Müller , C. Mück‐Lichtenfeld , F. Glorius , F. Dielmann , Angew. Chem. Int. Ed. 2022, 61, e202112344. 10.1002/anie.202112344 PMC9299603 34694044

[15] A. Das , P. Sarkar , S. Maji , S. K. Pati , S. K. Mandal , Angew. Chem. Int. Ed. 2022, 61, e202213614. 10.1002/anie.202213614 36259383

[16] R. Rudolf , N. I. Neuman , R. R. M. Walter , M. R. Ringenberg , B. Sarkar , Angew. Chem. Int. Ed. 2022, 61, e202200653. 10.1002/anie.202200653 PMC9322014 35286004

[17] R. Rudolf , D. Batman , N. Mehner , R. R. M. Walter , B. Sarkar , Chem. Eur. J. 2024, 30, e202400730. 38634285 10.1002/chem.202400730

[18] R. Rudolf , B. Sarkar , Inorg. Chem. 2024, 63, 23103–23117. 39576206 10.1021/acs.inorgchem.4c02631

[19] R. Rudolf , B. Sarkar , ChemistryEurope 2025, 3, e202400066.

[20] D. Kase , R. Haraguchi , Org. Lett. 2022, 24, 90–94. 34914400 10.1021/acs.orglett.1c03677

[21] S. Huang , Y. Wu , L. Huang , C. Hu , X. Yan , Chem. Asian J. 2022, 17, e202200281. 35502454 10.1002/asia.202200281

[22] D. Kase , T. Takada , N. Tsuji , T. Mitsuhashi , R. Haraguchi , Asian J. Org. Chem. 2023, 12, e202300108.

[23] J. R. Lamb , C. M. Brown , J. A. Johnson , C. C. Cummins , Chem. Sci. 2021, 12, 2699–2715. 34164037 10.1039/d0sc06465c PMC8179359

[24] A. Baishya , L. Kumar , M. K. Barman , T. Peddarao , S. Nembenna , ChemistrySelect 2016, 1, 498–503.

[25] L. M. Martínez‐Prieto , I. Cano , A. Márquez , E. A. Baquero , S. Tricard , L. Cusinato , I. Del Rosal , R. Poteau , Y. Coppel , K. Philippot , B. Chaudret , J. Cámpora , P. W. N. M. Van Leeuwen , Chem. Sci. 2017, 8, 2931–2941. 28451359 10.1039/c6sc05551f PMC5376718

[26] N. M. Gallagher , H. Z. Ye , S. Feng , J. Lopez , Y. G. Zhu , T. Van Voorhis , Y. Shao‐Horn , J. A. Johnson , Angew. Chem. Int. Ed. 2020, 59, 3952–3955. 10.1002/anie.201915534 31825136

[27] W.‐X. Zhang , L. Xu , Z. Xi , Chem. Commun. 2015, 51, 254–265. 10.1039/c4cc05291a 25298218

[28] L. Xu , W.‐X. Zhang , Z. Xi , Organometallics 2015, 34, 1787–1801.

[29] H. Karmakar , S. Anga , T. K. Panda , V. Chandrasekhar , RSC Adv. 2022, 12, 4501–4509. 35425514 10.1039/d2ra00242f PMC8981115

[30] M. Castillo , O. Barreda , A. K. Maity , B. Barraza , J. Lu , A. J. Metta‐Magaña , S. Fortier , J. Coord. Chem. 2016, 69, 2003–2014. 28216799 10.1080/00958972.2016.1167198 PMC5312970

[31] N. Patel , M. Arfeen , T. Singh , S. Bhagat , A. Sakhare , P. V. Bharatam , J. Comput. Chem. 2020, 41, 2624–2633. 10.1002/jcc.26417 32964506

[32] D. S. Patel , P. V. Bharatam , J. Phys. Chem. A 2011, 115, 7645–7655. 21650162 10.1021/jp111017u

[33] S. Bhagat , M. Arfeen , G. Das , M. Ramkumar , S. I. Khan , B. L. Tekwani , P. V. Bharatam , Bioorg. Chem. 2019, 91, 103094. 31376783 10.1016/j.bioorg.2019.103094

[34] N. Patel , R. Sood , P. V. Bharatam , Chem. Rev. 2018, 118, 8770–8785. 30113821 10.1021/acs.chemrev.8b00169

[35] A. Pleschke , A. Marhold , M. Schneider , A. Kolomeitsev , G.‐V. Röschenthaler , J. Fluorine Chem. 2004, 125, 1031–1038.

[36] R. Schwesinger , H. Schlemper , C. Hasenfratz , J. Willaredt , T. Dambacher , T. Breuer , C. Ottaway , M. Fletschinger , J. Boele , H. Fritz , Liebigs Ann. 1996, 1996, 1055–1081.

[37] S. Yao , T. Szilvási , Y. Xiong , C. Lorent , A. Ruzicka , M. Driess , Angew. Chem. Int. Ed. 2020, 59, 22043–22047. 10.1002/anie.202011598 PMC7756627 32841449

[38] N. Patel , M. Arfeen , R. Sood , S. Khullar , A. K. Chakraborti , S. K. Mandal , P. V. Bharatam , Chem. Eur. J. 2018, 24, 6418–6425. 29504658 10.1002/chem.201705999

[39] P. V. Bharatam , M. Arfeen , N. Patel , P. Jain , S. Bhatia , A. K. Chakraborti , S. Khullar , V. Gupta , S. K. Mandal , Chem. Eur. J. 2016, 22, 1088–1096. 26615987 10.1002/chem.201503618

[40] A. Kozma , G. Gopakumar , C. Fares , W. Thiel , M. Alcarazo , Chem. Eur. J. 2013, 19, 3542–3546. 23436732 10.1002/chem.201204186

[41] D. Himmel , I. Krossing , A. Schnepf , Angew. Chem. Int. Ed. 2014, 53, 370–374. 10.1002/anie.201300461 24243854

[42] D. Himmel , I. Krossing , A. Schnepf , Angew. Chem. Int. Ed. 2014, 53, 6047–6048. 10.1002/anie.201403078 24889370

[43] G. Frenking , Angew. Chem. Int. Ed. 2014, 53, 6040–6046. 10.1002/anie.201311022 24849466

[44] X.‐Y. Cui , C.‐H. Tan , D. Leow , Org. Biomol. Chem. 2019, 17, 4689–4699. 31032829 10.1039/c8ob02240b

[45] F. Carrillo‐Hermosilla , R. Fernández‐Galán , A. Ramos , D. Elorriaga , Molecules 2022, 27, 5962. 36144698 10.3390/molecules27185962 PMC9501388

[46] M. Findlater , N. J. Hill , A. H. Cowley , Dalton Trans. 2008, 4419. 18698443 10.1039/b800625c

[47] V. Ásgeirsson , B. O. Birgisson , R. Bjornsson , U. Becker , F. Neese , C. Riplinger , H. Jónsson , J. Chem. Theory Comput. 2021, 17, 4929–4945. 34275279 10.1021/acs.jctc.1c00462

[48] F. Neese , WIREs Comput. Mol. Sci. 2022, 12, e1606.

[49] R. Rashmi , P. K. Yadav , A. Seal , M. Paranjothy , U. Lourderaj , ChemPhysChem 2023, 24, e202200640. 36205532 10.1002/cphc.202200640

[50] K. Kanamori , J. D. Roberts , J. Am. Chem. Soc. 1983, 105, 4698–4701.

[51] Deposition Number(s) 2375078 (for **MesMII‐DippCDI**), 2375077 (for **PhMII‐DippCDI**), 2375076 (for **Dipp‐N(I))**, 2375065 (for **Dipp‐N(I)HOTf**), 2388350 (for **PhMII‐DippCDI‐Al**), 2449570 (for **PhMII‐TolCDI‐Rh**) contain(s) the supplementary crystallographic data for this paper. These data are provided free of charge by the joint Cambridge Crystallographic Data Centre and Fachinformationszentrum Karlsruhe Access Structures service.

[52] L. D. Pham , R. O. Smith‐Sweetser , B. Krupinsky , C. E. Dewey , J. R. Lamb , Angew. Chem. Int. Ed. 2023, 62, e202314376. 10.1002/anie.202314376 37824288

[53] R. A. Kunetskiy , I. Císařová , D. Šaman , I. M. Lyapkalo , Chem. Eur. J. 2009, 15, 9477–9485. 19655354 10.1002/chem.200901203

[54] G. Dubey , S. Awari , T. Singh , S. C. Sahoo , P. V. Bharatam , ChemPlusChem 2021, 86, 1416–1420. 34636173 10.1002/cplu.202100281

[55] J. Beerhues , M. Neubrand , S. Sobottka , N. I. Neuman , H. Aberhan , S. Chandra , B. Sarkar , Chem. Eur. J. 2021, 27, 6557–6568. 33502818 10.1002/chem.202100105 PMC8252451

[56] F. Stein , S. Suhr , A. S. Hazari , R. Walter , M. Nößler , B. Sarkar , Chem. Eur. J. 2023, 29, e202300771. 37042487 10.1002/chem.202300771

[57] A. Varenikov , M. Gandelman , M. S. Sigman , J. Am. Chem. Soc. 2024, 146, 19474–19488. 38963077 10.1021/jacs.4c05799

[58] Y. K. Kang , H. S. Park , J. Mol. Struct.: THEOCHEM 2004, 676, 171–176.

[59] A. D. Becke , K. E. Edgecombe , J. Chem. Phys. 1990, 92, 5397–5403.

[60] T. Lu , F. Chen , J. Comput. Chem. 2012, 33, 580–592. 22162017 10.1002/jcc.22885

[61] A. Rösch , F. Seifert , V. Vass , H. Görls , R. Kretschmer , New J. Chem. 2021, 45, 972–981.

[62] Y. R. Yepes , Á. Mesías‐Salazar , A. Becerra , C. G. Daniliuc , A. Ramos , R. Fernández‐Galán , A. Rodríguez‐Diéguez , A. Antiñolo , F. Carrillo‐Hermosilla , Organometallics 2021, 40, 2859–2869.

[63] G. R. Fulmer , A. J. M. Miller , N. H. Sherden , H. E. Gottlieb , A. Nudelman , B. M. Stoltz , J. E. Bercaw , K. I. Goldberg , Organometallics 2010, 29, 2176–2179.

