# Supplementary material for: Exploring Mesoionic Imine‐Carbodiimide (MII‐CDI) Adducts: 1,3 H‐Shift, N(I) Compounds and Guanidinate‐Type Ligands
Source: Angew Chem Int Ed Engl. 2025 Jul 16;64(34):e202502097. doi: 10.1002/anie.202502097 (PMC12363636; doi:10.1002/anie.202502097)
Supplement: Supplementary file 1 — Supporting Information [file ANIE-64-e202502097-s002.docx]

Supporting Information

Exploring Mesoionic Imine-Carbodiimide (MII-CDI) Adducts: 1,3 H-Shift, N(I) Compounds and Guanidinate-Type Ligands

Alok Mahata, Richard Rudolf, Robert R. M. Walter, Dr. Nicolás. I. Neuman, Prof. Dr. Biprajit Sarkar

1. Instrumental ................................................................................................................................. 2

2. Synthetic procedures ................................................................................................................... 3

3. NMR Spectroscopy................................................................................……………………………9

4. NMR Spectroscopy controlled reactions...............................................…………………………….20

5. Crystal structures and crystallographic data.................................................................................24

6. Cyclic voltammetry, UV-Vis-NIR and EPR Spectro-electrochemistry...........................................33

7. Computational details ...................................................................................................................35

8. References ...................................................................................................................................67

**1. Instrumental**

Unless otherwise noted, all reactions were performed using standard Schlenk-line techniques under an intert atmosphere of argon (Linde, Argon 4.8, purity ≥ 99.998) or an MBraun glove box fitted with a gas purification and recirculation unit. Commercially available chemicals were used without further purification. Mesoionic imines, diphenyl carbodiimide and (Cp*RhCl_2_)_2_ were synthesized using literature reported procedures.^[1]^ *o*-Difluorobenzene was dried and distilled from CaH_2_ and stored over 4Å molecular sieve. Other solvents were available from MBRAUN MB-SPS-800 solvent system and additionally degassed using standard techniques. ^1^H NMR, ^13^C NMR and ^19^F NMR were recorded on Brucker 250 and 400 MHz spectrometer. Chemical shifts are reported in ppm (relative to the TMS signal) with reference to the residual solvent peaks.^[2]^ Residual signal of water and grease peak are denoted by asterisks (*) and other solvent or non-separable impurities are denoted by hashtags (#) in some cases. Multiplets are reported as follows: singlet (s), duplet (d), triplet (t), quartet (q), septet (sept), and multiplate (m). NMRs under the exclusion of air were conducted in J. Young’s NMR tubes oven dried and flushed with argon prior to use. Mass spectrometry was performed on a microTOFQ Bruker Daltonics. Elemental analysis was performed on an Elementar VarioMICRO cube.

Cyclic voltammograms were recorded with a Palm Sens 4 potentiostat by working in anhydrous and degassed *O*-Difluorobenzene with 0.1 м NBu_4_PF_6_ (dried, > 99.0%, electrochemical grade, Fluka) as supporting electrolyte. Concentrations of the compounds were about 1·10^-4^ м. A three-electrode setup was used with a glassy carbon working electrode, a coiled platinum wire as counter electrode, and a coiled silver wire as a pseudo-reference electrode. The ferrocene/ferrocenium couple was used as internal reference. UV/Vis spectra were recorded on a J&M TIDAS UV-VisNIR spectrophotometer. UV/vis spectroelectrochemical measurements were carried out in an optically transparent thin-layer electrochemical (OTTLE)^[3]^ cell (CaF_2_ windows) with a gold mesh working electrode, a platinum-mesh counter electrode, and a silver-foil pseudo reference. EPR spectra at the X-band frequency (ca. 9.5 GHz) were obtained with a Magnettech MS 5000 benchtop EPR spectrometer equipped with a rectangular TE 102 cavity and a TC HO4 temperature controller. The measurements were carried out in synthetic quartz glass tubes. For EPR spectroelectrochemistry, a three-electrode setup was employed using two Teflon-coated platinum wires (0.005 in. bare and 0.008 in. coated) as the working and counter electrodes and a Teflon-coated silver wire (0.005 in. bare and 0.007 in coated) as the pseudo reference electrode. The experiments were carried out in dry *O*-Difluorobenzene containing 0.1 M Bu_4_NPF_6_ as the supporting electrolyte.

X-ray data were collected on a Bruker Kappa Apex Duo system at 295(2), 150(2) K or 100(2) K, using graphite-monochromated MoΚα radiation (λα= 0.71073 Å). The strategy for the data collection was evaluated by using the Smart software. The data were collected by the standard omega scan or omega + phi scan techniques, and were scaled and reduced using Saint+ and SADABS software. The structures were solved by direct methods using SHELXS-97 or intrinsic phasing using SHELXL-2014/7 and refined by full matrix least-squares, refining on F2 . Non-hydrogen atoms were refined anisotropically.^[4-6]^ Structures were solved using SHELXS-97^[4]^ and the software OLEX2,^[6]^ while refinement was carried out on F2 against all independent reflections by the full matrix least-squares method using the SHELXL-97 program. All non-hydrogen atoms were refined using anisotropic thermal parameters.

**2. Synthetic procedures**

Synthesis of **MesMII-DippCDI**

A mixture of **MesMII** (167 mg, 0.5 mmol, 1 equiv.) and Bis(2,6-diisopropylphenyl)carbodiimide (182 mg, 0.5 mmol, 1 equiv.) was added to an oven-dried Schlenk flask and dissolved in dry acetonitrile (20 mL). The reaction mixture was stirred at room temperature overnight. During this time, the color of the mixture changed from reddish-yellow to light yellow, and a light yellow precipitate formed. The precipitate was collected by filtration, washed with 5 mL of diethyl ether (Et_2_O), yielding the compound **MesMII-DippCDI**. Single crystals suitable for XRD were obtained by keeping the filtrate at -20 °C for 3 days. Yield: 244 mg (0.35 mmol, 70%).

**^1^H-NMR** (CD_2_Cl_2_, 250 MHz) *δ*: 7.02 – 6.71 (m, 10H, Ar-*H*), 4.34 (s, 1H, N-*H*), 3.54 (s, 3H, N-C*H_3_*), 3.08 (sept, 2H, C*H*(CH_3_)_2_), 2.78 (sept, 2H, C*H*(CH_3_)_2_), 2.29 – 2.23 (3s, 12H, Aryl-*o*-C*H*_3_), 1.86 (s, 6H, Aryl-*p*-C*H*_3_), 1.10 (d, 6H, CH(C*H*_3_)_2_), 0.83 - 0.78 (m, 18H, CH(C*H*_3_)_2_) ppm.

**^13^C{^1^H}-NMR** (CD_2_Cl_2_, 63 MHz) *δ*: 151.1, 150.4, 148.1, 147.5, 142.5, 139.7, 139.1, 137.4, 136.5, 132.4, 128.9, 128.8, 125.9, 125.6, 125.4, 122.9, 122.3, 120.7, 37.8, 28.0, 27.8, 25.2, 24.0, 21.6, 21.3, 20.6, 17.7 ppm.

**EA**: calc. (C_46_H_60_N_6_, 697.03 g/mol) C 79.27 H 8.68 N 12.06; found C 79.02 H 8.60 N 11.91

**MS(ESI)**: m/z = 697.49 [M+H]^+^.

**HRMS(ESI)**: calc (C_46_H_61_N_6_) m/z = 697.4952; found m/z = 697.4936.

Synthesis of **PhMII-DippCDI**

A mixture of **PhMII** (250 mg, 1 mmol, 1 equiv.) and Bis(2,6-diisopropylphenyl)carbodiimide (363 mg, 1 mmol, 1 equiv.) was added to an oven-dried Schlenk flask and dissolved in dry acetonitrile (20 mL). The reaction mixture was stirred at room temperature overnight. During this time, the color of the mixture changed from reddish-yellow to light yellow, and a light yellow precipitate formed. The precipitate was collected by filtration, washed with 5 mL of diethyl ether (Et_2_O), yielding the compound **PhMII-DippCDI**. Single crystals suitable for XRD were obtained by keeping the filtrate at -20 °C for 3 days. Yield: 380 mg (0.62 mmol, 62%).

**^1^H-NMR** (CD_2_Cl_2_, 250 MHz) *δ*: 7.96 – 7.92 (m, 2H, Ar-*H*), 7.75 – 7.71 (m, 2H, Ar-*H*), 7.58 – 7.52 (m, 6H, Ar-*H*), 7.13 – 6.97 (m, 6H, Ar-*H*), 4.68 (s, 1H, N-*H*), 4.04 (s, 3H, N-C*H_3_*), 3.03 (sept, 4H, C*H*(CH_3_)_2_), 1.13 - 0.79 (m, 24H, CH(C*H*_3_)_2_) ppm.

**^13^C{^1^H}-NMR** (CD_2_Cl_2_, 63 MHz) *δ*: 158.9, 154.0, 147.4, 145.1, 136.1, 130.5, 130.4, 129.6, 129.4, 127.1, 126.5, 125.5, 123.5, 123.3, 39.4, 28.6, 28.5, 26.1, 23.1 ppm.

**EA**: calc. (C_40_H_48_N_6_, 612.86 g/mol) C 78.39 H 7.89 N 13.71; found C 74.60 H 7.88 N 12.88

**MS(ESI)**: m/z = 613.40 [M+H]^+^.

**HRMS(ESI)**: calc (C_40_H_49_N_6_) m/z = 613.4013; found m/z = 613.4001.

Synthesis of **PhMII-PhCDI**

A mixture of **PhMII** (250 mg, 1 mmol, 1 equiv.) and diphenylcarbodiimide (194 mg, 1 mmol, 1 equiv.) was added to an oven-dried Schlenk flask and dissolved in dry acetonitrile (20 mL). The reaction mixture was stirred at room temperature overnight. During this time, the color of the mixture changed from reddish-yellow to light yellow. All the volatiles were removed under vaccum. 30 mL of pentane was added to the remaining solid and stirred for 5 minutes. A yellow precipitate formed, was collected by filtration washed with 5 mL of diethyl ether (Et_2_O), yielding the compound **PhMII-PhCDI**. Yield: 240 mg (0.54 mmol, 54%).

**^1^H-NMR** (CD_2_Cl_2_, 250 MHz) *δ*: 7.95 – 7.92 (m, 2H, Ar-*H*), 7.58 – 7.36 (m, 8H, Ar-*H*), 7.12 – 7.05 (m, 4H, Ar-*H*), 6.90 – 6.85 (m, 6H, Ar-*H*), 3.90 (s, 3H, N-C*H_3_*) ppm.

**^13^C{^1^H}-NMR** (CD_2_Cl_2_, 63 MHz) *δ*: 152.4, 135.7, 130.5, 130.3, 129.8, 129.6, 129.1, 128.9, 127.4, 125.7, 124.6, 123.1, 122.1, 39.1 ppm.

**EA**: calc. (C_28_H_24_N_6_, 444.54 g/mol) C 75.65 H 5.44 N 18.91; found C 70.23 H 5.43 N 17.53

**MS(ESI)**: m/z = 445.21 [M+H]^+^.

**HRMS(ESI)**: calc (C_28_H_25_N_6_) m/z = 445.2135; found m/z = 445.2122.

Synthesis of **PhMII-TolCDI**

A mixture of **PhMII** (250 mg, 1 mmol, 1 equiv.) and di-*p*-tolyl-carbodiimide (229 mg, 1 mmol, 1 equiv.) was added to an oven-dried Schlenk flask and dissolved in acetonitrile (20 mL). The reaction mixture was stirred at room temperature overnight. During this time, the color of the mixture changed from reddish-yellow to light yellow. All the volatiles were removed under vaccum. 30 mL of pentane was added to the remaining solid and titurated for 10 minutes. A yellow precipitate formed, was collected by filtration washed with 10 mL of pentane, yielding the compound **PhMII-TolCDI**. Yield: 388 mg (0.82 mmol, 82%).

**^1^H-NMR** (CD_2_Cl_2_, 250 MHz) *δ*: 8.02 – 7.99 (m, 2H, Ar-*H*), 7.58 – 7.41 (m, 8H, Ar-*H*), 6.91 – 6.67 (m, 8H, Tol-*H*), 3.91 (s, 3H, N-C*H_3_*), 3.23 (s, 6H, Tol-C*H_3_*) ppm.

**^13^C{^1^H}-NMR** (CD_2_Cl_2_, 63 MHz) *δ*: 152.7, 136.6, 130.5, 129.7, 129.5, 129.4, 129.2, 127.2, 125.8, 124.1, 123.5, 121.7, 38.9, 20.9 ppm.

**EA**: calc. (C_30_H_28_N_6_, 472.59 g/mol) C 76.24 H 5.97 N 17.78; found C 75.73 H 6.03 N 17.46

**MS(ESI)**: m/z = 473.24 [M+H]^+^.

**HRMS(ESI)**: calc (C_30_H_29_N_6_) m/z = 473.2448; found m/z = 473.2443

Synthesis of **PhMII-iPrCDI**

A mixture of **PhMII** (250 mg, 1 mmol, 1 equiv.) and di-isopropyl-carbodiimide (379 mg, 3 mmol, 3 equiv.) was added to an oven-dried Schlenk flask and dissolved in acetonitrile (20 mL). The reaction mixture was heated at 65 °C for 3 days. All the volatiles were removed under vaccum. Brown oil compound was formed. Which was washed several times with penatne. Then it was redissolved in minium amount of Et_2_O and reprecipitate with pentane by titurating for 15 minutes. A off white precipitate formed, was collected by filtration washed with 10 mL of pentane, yielding the compound **PhMII-iPrCDI**. Because of formation of several side products we could not able to a pure compound. However, the formation of the product could be identified by ^1^H NMR and HRMS measurement. Unoptimized Yield: 30 mg (0.08 mmol, 8%).

**^1^H-NMR** (CD_2_Cl_2_, 250 MHz) *δ*: 7.92 – 7.89 (m, 2H, Ar-*H*), 7.71 – 7.69 (m, 2H, Ar-*H*), 7.54 – 7.47 (m, 6H, Ar-*H*), 4.12 (s, 3H, N-C*H_3_*), 3.50 (bs, 2H, C*H*(CH_3_)_2_), 0.94 - 0.91 (m, 12H, CH(C*H*_3_)_2_) ppm.

**MS(ESI)**: m/z = 377.24 [M+H]^+^.

**HRMS(ESI)**: calc (C_22_H_29_N_6_) m/z = 377.2448; found m/z = 377.2444

Methylation of **PhMII-DippCDI** for the synthesis of **Dipp-N(I)HOTf**

The adduct **PhMII-DippCDI** (610 mg, 1 mmol, 1 equiv.) and K_2_CO_3_ (83 mg, 0.6 mmol, 1,2 equiv.) were placed into an oven-dried Schlenk flask and dissolved in dry MeCN (30 mL). Methyliodide (456mg, 0.2 mL, 3.2 mmol, 3.2 equiv.) was added, and the solution was heated to 65 °C in the closed flask. The stopcock was briefly opened to release pressure and then closed. The mixture was stirred at 65 °C for 2 days. After cooling to room temperature, volatiles were removed under reduced pressure. The residue was extracted with DCM (10 mL), and volatiles were again removed under vacuum. The remaining solid was washed with Et_2_O, dried under vacuum, and used in the next step without further purification.

The crude product was dissolved in 15 mL of dry DCM in a Schlenk flask, and a solution of MeOTf (820 mg, 0.6 mL, 5 mmol, 5 equiv.) in 10 mL of DCM was added to it at –78 °C. The reaction mixture was slowly warmed to room temperature and stirred for 3 days. Volatiles were removed under vacuum, and the solid was washed with 30 mL of Et_2_O. The crude product was dissolved in a small amount of acetonitrile and recrystallized by slow diffusion of Et_2_O. After 3 days, the product **N(I)HOTf** crystallized as colorless needles. The product was isolated by decanting the solvents, washed with 10 mL Et_2_O and dried under reduced pressure. Yield: 235 mg (0.25 mmol, 25%).

**^1^H-NMR** (CD_2_Cl_2_, 250 MHz) *δ*: 8.02 – 7.75 (m, 10H, Ar-*H*), 7.47 – 7.41 (m, 2H, Ar-*H*), 7.29 – 7.20 (m, 4H, Ar-*H*), 4.42 (s, 1H, N-*H*), 4.14 (s, 3H, N-C*H_3_*), 3.63 (s, 3H, N-C*H_3_*), 2.82 – 2.57 (m, 2H, C*H*(CH_3_)_2_ & 3H, N-C*H_3_*), 1.31 - 0.74 (m, 24H, CH(C*H*_3_)_2_) ppm.

**^13^C{^1^H}-NMR** (CD_2_Cl_2_, 63 MHz) *δ*: 151.5, 145.0, 144.9, 144.5, 140.8, 140.0, 138.0,134.0, 133.9, 133.5, 132.6, 131.9, 131.5, 131.2, 131.1, 130.7, 127.1, 127.0, 126.8, 125.9, 125.8, 123.54, 121.6, 118.4, 49.7, 47.1, 40.6, 30.0, 29.9, 29.5, 29.4, 26.5, 26.4, 26.2, 26.1, 25.2, 23.0, 22.7, 22.3, 22.1 ppm.

**^19^F-NMR** (CD_2_Cl_2_, 235 MHz) *δ*: -78.8 ppm.

**EA**: calc. (C_44_H_54_N_6_F_6_O_6_S_2_, 941.06 g/mol) C 56.16 H 5.78 N 8.93; found C 56.10 H 5.95 N 8.91

**MS(ESI)**: m/z = 641.43 [M-HOTf-OTf^−^]^+^.

**HRMS(ESI)**: calc (C_42_H_53_N_6_) m/z = 641.4326; found m/z = 641.4332.

Deprotonation of **Dipp-N(I)HOTf** for the synthesis of **Dipp-N(I)**

**Dipp-N(I)HOTf** (235 g, 0.25 mmol, 1 equiv.) and NaHMDS (46 mg, 0.25 mmol, 1 equiv.) were placed into an oven-dried Schlenk-flask and cooled to –20°C. Dry o-DFB (20 mL) was added and the solution was stirred for one hour and an additional hour at RT. Volatiles were removed under reduced pressure and the residue was extracted with DCM (30 mL). The solution was filtered and volatiles were again removed under vacuum. The remaining solid was washed with Et_2_O, dried under vacuum to get pure **Dipp-N(I)**. Because of formation of several rotational isomer, we could not able to get a resolved ^1^H and ^13^C NMR spectra. ^1^H-NMR spectra of N(I) at variable temperatures were recorded which confirm the presence of rotational isomers (Figure S10). Yield: 182 mg (0.23 mmol, 92%).

**^19^F-NMR** (CDCl_3_, 376 MHz) *δ*: -78.2 ppm.

**EA**: calc. (C_43_H_53_N_6_F_3_O_3_S, 941.06 g/mol) C 65.29 H 6.75 N 10.62; found C 65.26 H 6.81 N 10.61

**MS(ESI)**: m/z = 641.43 [M-OTf^−^]^+^.

**HRMS(ESI)**: calc (C_42_H_53_N_6_) m/z = 641.4326; found m/z = 641.4328

Methylation of **PhMII-TolCDI** for the synthesis of **Tol-N(I)**

The adduct **PhMII-TolCDI** (473 mg, 1 mmol, 1 equiv.) and K_2_CO_3_ (276 mg, 2 mmol, 4 equiv.) were placed into an oven-dried Schlenk flask and dissolved in dry MeCN (20 mL). Methyliodide (456mg, 0.2 mL, 3.2 mmol, 3.2 equiv.) was added, and the solution was heated to 65 °C in the closed flask. The stopcock was briefly opened to release pressure and then closed. The mixture was stirred at 65 °C for 3 days. After cooling to room temperature, volatiles were removed under reduced pressure. The residue was extracted with DCM (10 mL), and volatiles were again removed under vacuum. The pure product as off white solid was washed with Et_2_O, dried under reduced pressure. Yield: 490 mg (0.78 mmol, 78%).

**^1^H-NMR** (CD_2_Cl_2_, 250 MHz) *δ*: 7.83 – 7.59 (m, 10H, Ar-*H*), 6.95 (d, 7.5 Hz, 2H, Tol-*H*), 6.68 (d, 7.5 Hz, 2H, Tol-*H*)), 4.15 (s, 3H, N-C*H_3_*), 2.68 (s, 6H, N-C*H_3_*), 2.23 (s, 6H, Tol-C*H_3_*) ppm.

**^13^C{^1^H}-NMR** (CD_2_Cl_2_, 63 MHz) *δ*: 159.1, 146.1, 141.1, 135.9, 134.8, 131.9, 131.3,130.6, 130.4, 130.1, 129.5, 128.6, 124.4, 123.7, 122.4, 40.4, 39.4, 21.0 ppm.

**EA**: calc. (C_32_H_33_N_6_I, 628.56 g/mol) C 61.15 H 5.29 N 13.37; found C 59.63 H 5.26 N 12.75

**MS(ESI)**: m/z = 501.28 [M-I^−^]^+^.

**HRMS(ESI)**: calc (C_32_H_33_N_6_) m/z = 501.2761; found m/z = 501.2753.

Protonation of **Tol-N(I)** for the synthesis of **Tol-N(I)HOTf**

**Tol-N(I)** (157 mg, 0.25 mmol, 1 equiv.) and DMF.HOTf (167 mg, 0.75 mmol, 3 equiv.) were placed into an oven-dried Schlenk-flask and DCM (10 mL) was added and the solution was stirred for 30 minutes at RT. Volatiles were removed under reduced pressure. The remaining solid was washed several times with Et_2_O (50mL), dried under vacuum to get pure **Tol-N(I)HOTf** as off white solid. Slow diffusion of Et_2_O over a concentrated solution of the product provide single cristals suitable for XRD analysis. Yield: 152 mg (0.19 mmol, 76%).

**^1^H-NMR** (CD_2_Cl_2_, 250 MHz) *δ*: 7.96 – 7.94 (m, 2H, Ar-*H*), 7.83 – 7.69 (m, 8H, Ar-*H*), 7.00 (d, 7.5 Hz, 2H, Tol-*H*), 6.68 (d, 7.5 Hz, 2H, Tol-*H*), 4.35 (s, 3H, N-C*H_3_*), 3.01 (s, 6H, N-C*H_3_*), 2.26 (s, 6H, Tol-C*H_3_*) ppm.

**^13^C{^1^H}-NMR** (CD_2_Cl_2_, 63 MHz) *δ*: 157.4, 140.2, 138.3, 138.1, 134.6, 133.8, 133.3,132.7, 131.5, 130.8, 125.4, 124.4, 120.5, 43.4, 40.8, 21.3 ppm.

**EA**: calc. (C_34_H_34_N_6_F_6_O_6_S_2_, 800.79 g/mol) C 51.00 H 4.28 N 10.49; found C 50.82 H 4.30 N 10.37

**MS(ESI)**: m/z = 501.27 [M-HOTf-OTf^−^]^+^.

**HRMS(ESI)**: calc (C_32_H_33_N_6_) m/z = 501.2761; found m/z = 501.2748.

Reaction of **PhMII-DippCDI** with AlMe_3_ for the synthesis of **PhMII-DippCDIAl**

The adduct **PhMII-DippCDI** (122 mg, 0.2 mmol, 1 equiv.) was placed into an oven-dried Schlenk flask and dissolved in dry Toluene (20 mL). and cooled to –78 °C. AlMe_3_ (2.0 M in Toluene)(0.1 mL, 0.2 mmol, 1 equv.) was added and the solution was stirred for one hour at that temperature and warm it room temperature. The reaction mixture was heated at 90 °C for overnight. After cooling down to room temperature, all volatiles were removed under reduced pressure and the residue was washed with pentane (20 mL). The remaining solid was dried under vacuum to get the desired complex. Because of high sensitivity of the complex towards moisture, we couldn’t able to get a good HRMS, Elemental analysis and ^13^C NMR spectra.

**^1^H-NMR** (Tol-d_8_, 250 MHz) *δ*: 7.73 (d, 7.5 Hz, 2H, Ar-*H*), 7.22 – 6.97 (m, 14H, Ar-*H*, along with residual solvent signal), 3.68 (sept, 2H, C*H*(CH_3_)_2_), 3.49 (sept, 2H, C*H*(CH_3_)_2_), 2.60 (s, 3H, N-C*H_3_*), 1.28 – 0.94 (m, 24H, CH(C*H*_3_)_2_), -0.07 – -0.14 (two singlet, 6H, Al-C*H_3_*) ppm.

Reaction of **PhMII-TolCDI** with (Cp*RhCl_2_)_2_ for the synthesis of **PhMII-TolCDI-Rh**

The adduct **PhMII-TolCDI** (237 mg, 0.5 mmol, 1 equiv.) and KH (60 mg, 0.75 mmol, 1.5 equiv.) were placed into an oven-dried Schlenk flask and dissolved in dry THF (20 mL) and the solution was stirred for one hour at that temperature, afterwards was heated at 60 °C for overnight. After cooling down to room temperature, the light orange solution was transferred to a pre-cooled THF solution of (Cp*RhCl_2_)_2_ (154 mg, 0.25 mmol, 0.5 equiv.) at –78 °C under argon atmosphere. The reaction mixture was slowly warm up to room temperature and stirred againg for 3 hours. All volatiles were removed under reduced pressure The residue was extracted with DCM (10 mL), and volatiles were again removed under vacuum. The crude product **PhMII-TolCDI-Rh** as orange-yellow solid was isolated after washing with Et_2_O (20 mL), and dried under reduced pressure. Yield: 194 mg (0.26 mmol, 52%).

Alternatively, this could be synthesize by without addition of KH also. The adduct **PhMII-TolCDI** (237 mg, 0.5 mmol, 1 equiv.) and (Cp*RhCl_2_)_2_ (77 mg, 0.125 mmol, 0.125 equiv.) were placed into an oven-dried Schlenk flask and dissolved in dry toluene (20 mL) and the solution was stirred at 90 °C for 3 days. After cooling down to room temperature, all the volatiles were evaporated under vaccum. Obtained solid was washed with Et_2_O (20 mL), and the orange-yellow precipitate formed are recrystallized by dissolved again in minium amount of DCM and slow diffusion of Et_2_O over it at 5 °C. After 3 days the pure product was isolated as orange-red crystal, which also use for single crystal XRD measurement. Recrystalization was necessary in this case to get rid off guanidinium chloride formed in this reaction as side product. Yield: 61 mg (0.08 mmol, 32%).

**^1^H-NMR** (CD_2_Cl_2_, 250 MHz) *δ*: 7.87 – 7.84 (m, 2H, Ar-*H*), 7.52 – 7.41 (m, 8H, Ar-*H*), 6.92 – 6.83 (m, 8H, Tol-*H*), 3.73 (s, 3H, N-C*H_3_*), 2.23 (s, 6H, Tol-C*H_3_*), 1.36 (s, 15H, Cp*-C*H_3_*) ppm.

**^13^C{^1^H}-NMR** (CD_2_Cl_2_, 63 MHz) *δ*: 145.9, 136.3, 129.6, 129.5, 129.1, 128.5, 125.1, 124.8, 123.7, 91.0, 90.9, 38.9, 21.1, 9.5 ppm.

**MS(ESI)**: m/z = 709.25 [M-Cl^-^]^+^.

**HRMS(ESI)**: calc (C_40_H_42_N_6_Rh) m/z = 709.2521; found m/z = 709.2517

**3. NMR Spectroscopy**


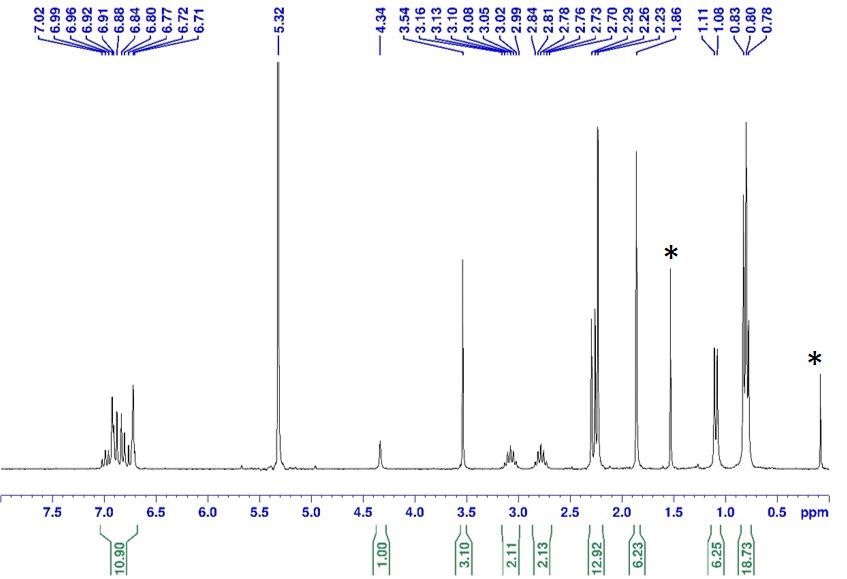


**Figure S1:** ^1^H-NMR-Spectrum of **MesMII-DippCDI** (CD_2_Cl_2_, 250 MHz).


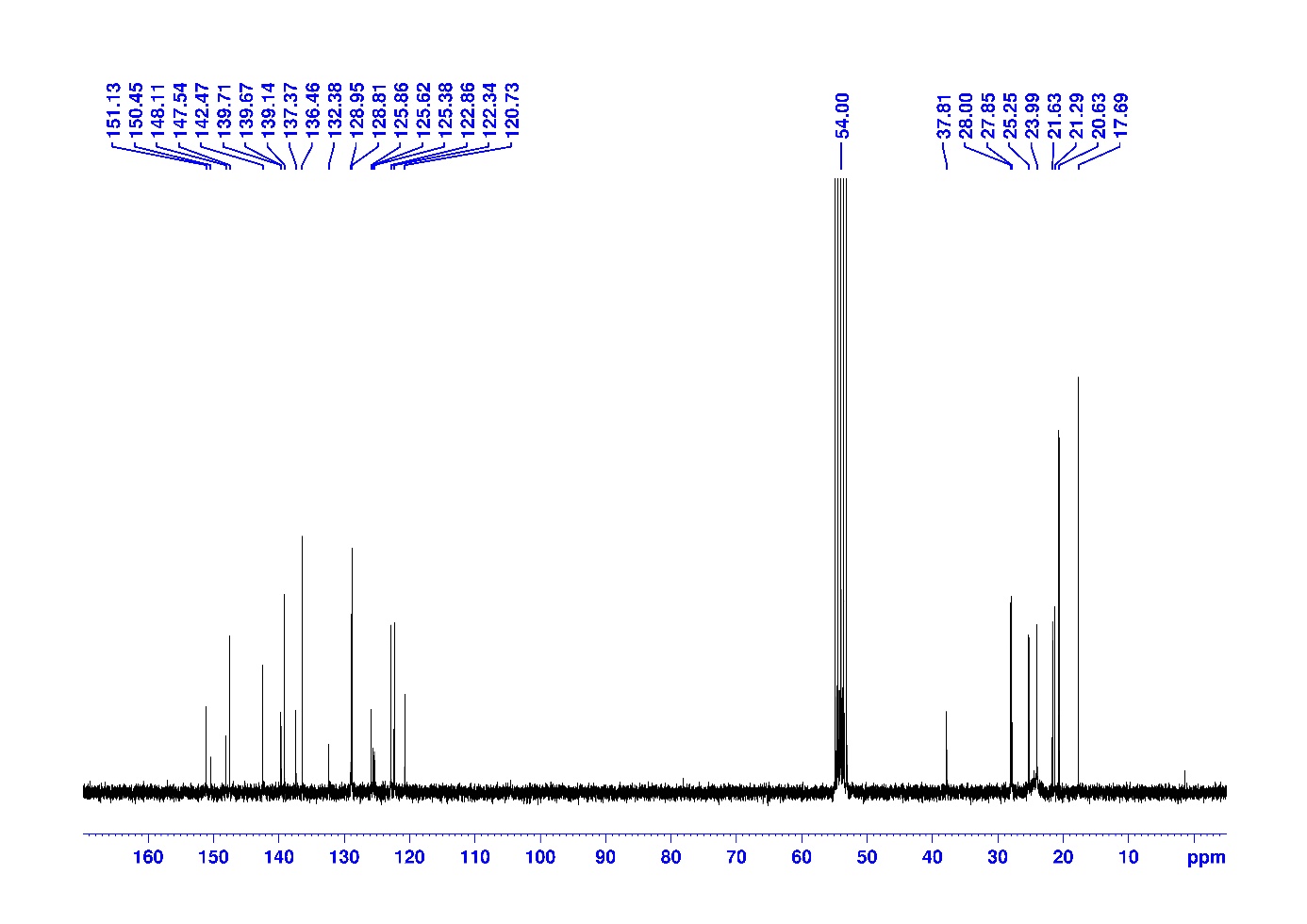


**Figure S2:** ^13^C{^1^H}-NMR-Spectrum of **MesMII-DippCDI** (CD_2_Cl_2_, 63 MHz).


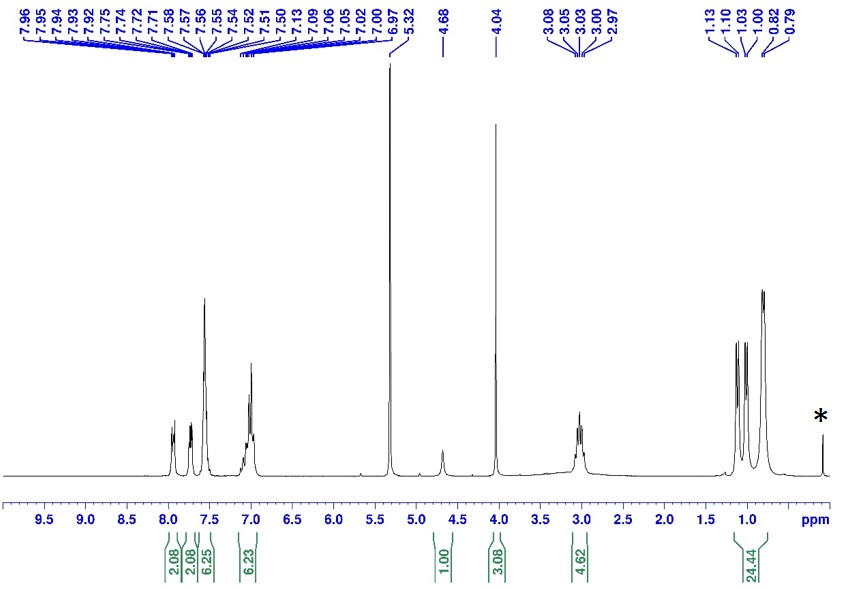


**Figure S3:** ^1^H-NMR-Spectrum of **PhMII-DippCDI** (CD_2_Cl_2_, 250 MHz).


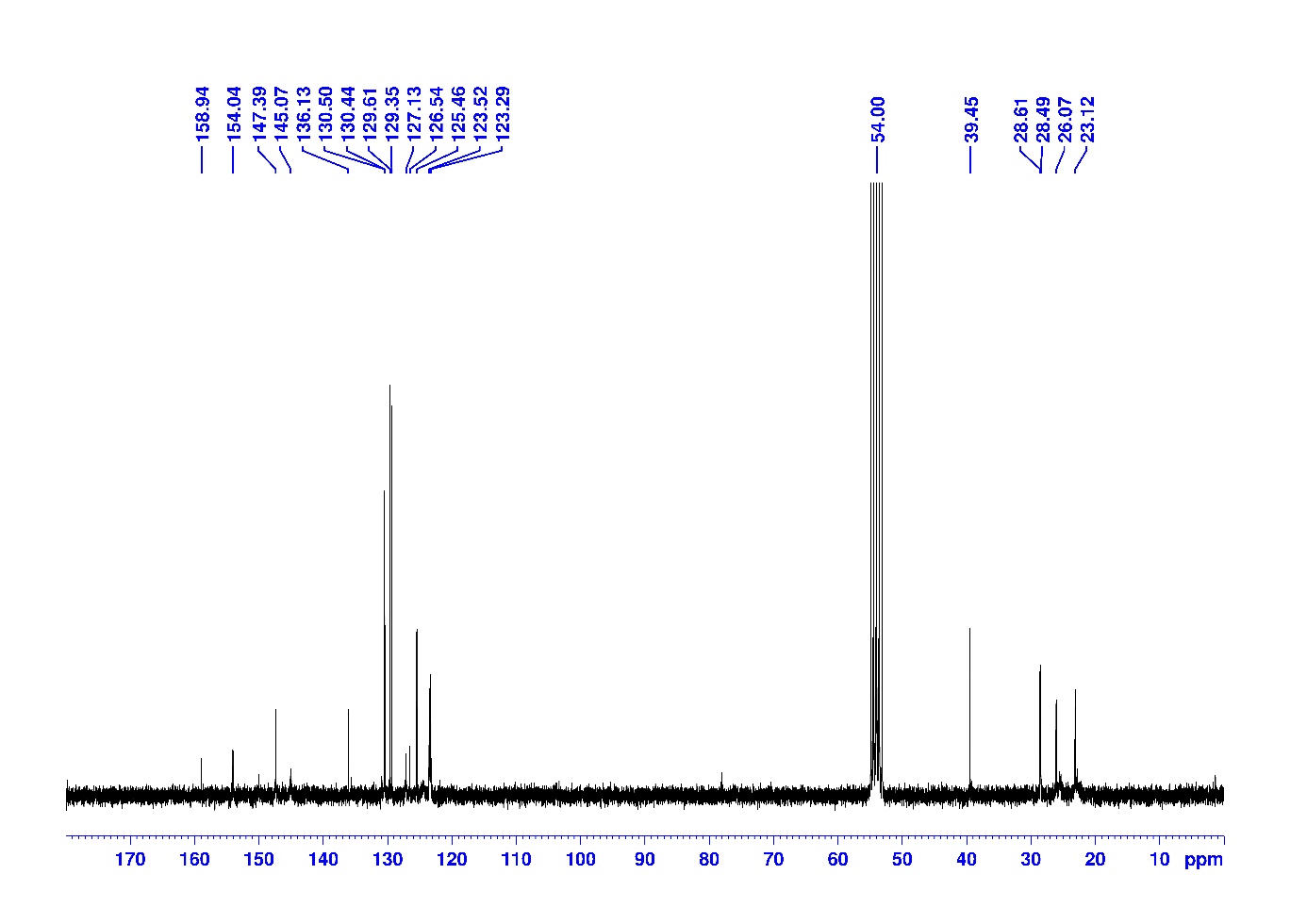


**Figure S4:** ^13^C{^1^H}-NMR-Spectrum of **PhMII-DippCDI** (CD_2_Cl_2_, 63 MHz).


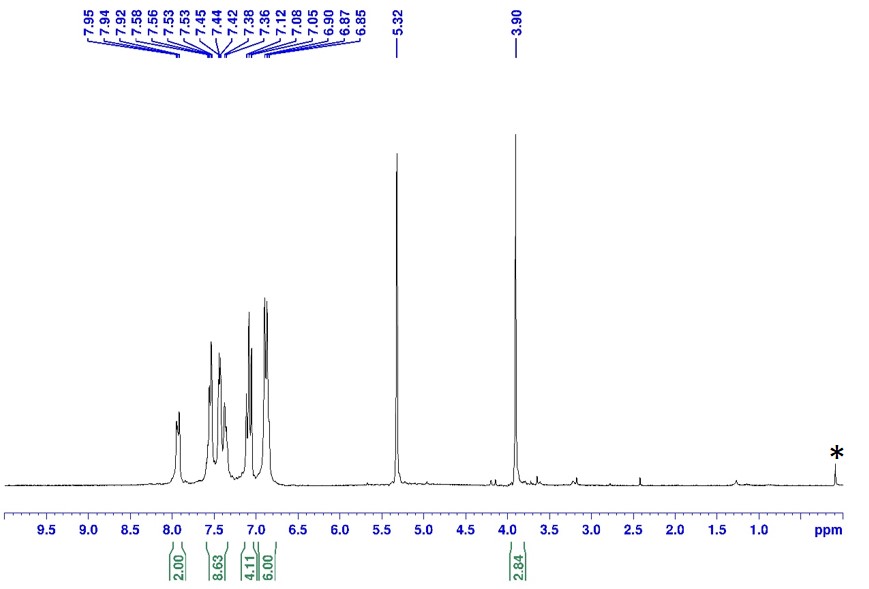


**Figure S5:** ^1^H-NMR-Spectrum of **PhMII-PhCDI** (CD_2_Cl_2_, 250 MHz).


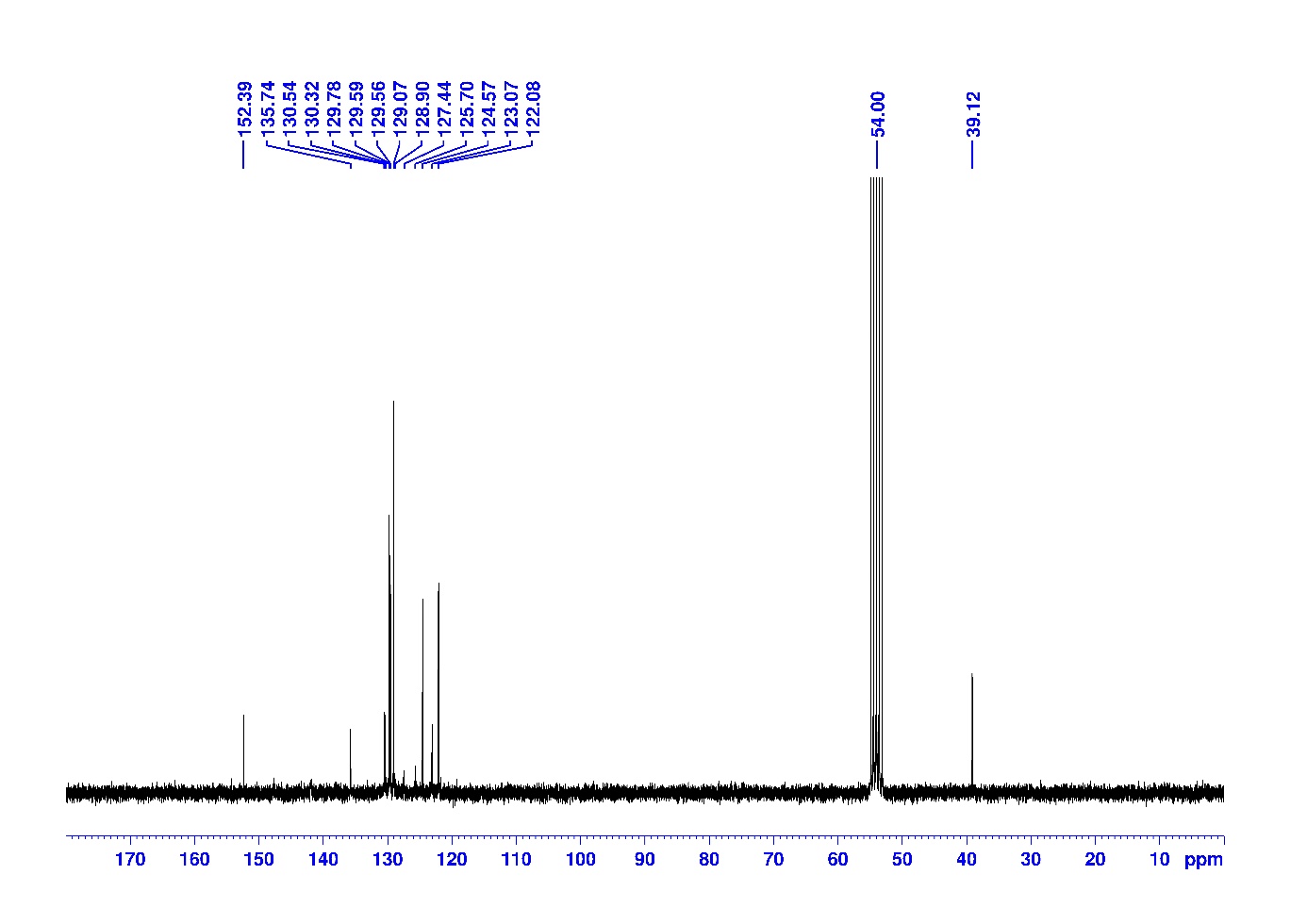


**Figure S6:** ^13^C{^1^H}-NMR-Spectrum of **PhMII-PhCDI** (CD_2_Cl_2_, 63 MHz).


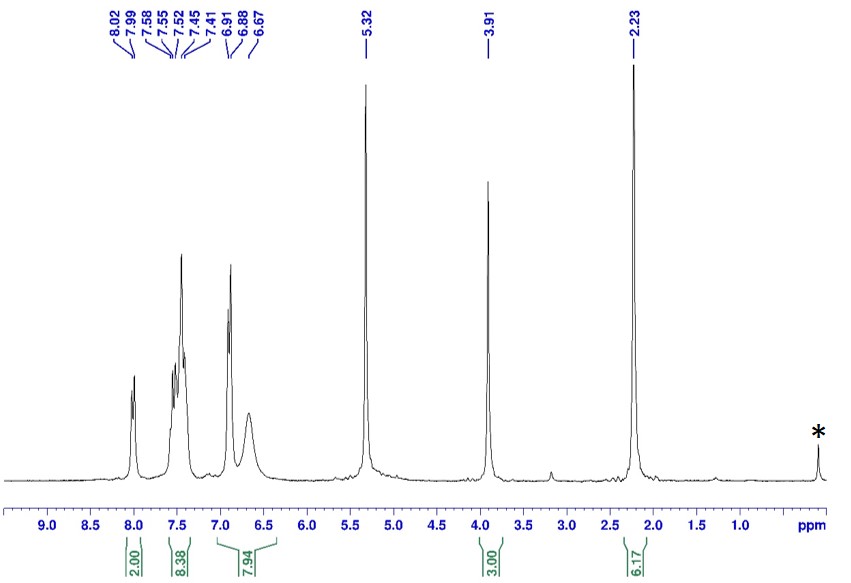


**Figure S7:** ^1^H-NMR-Spectrum of **PhMII-TolCDI** (CD_2_Cl_2_, 250 MHz).


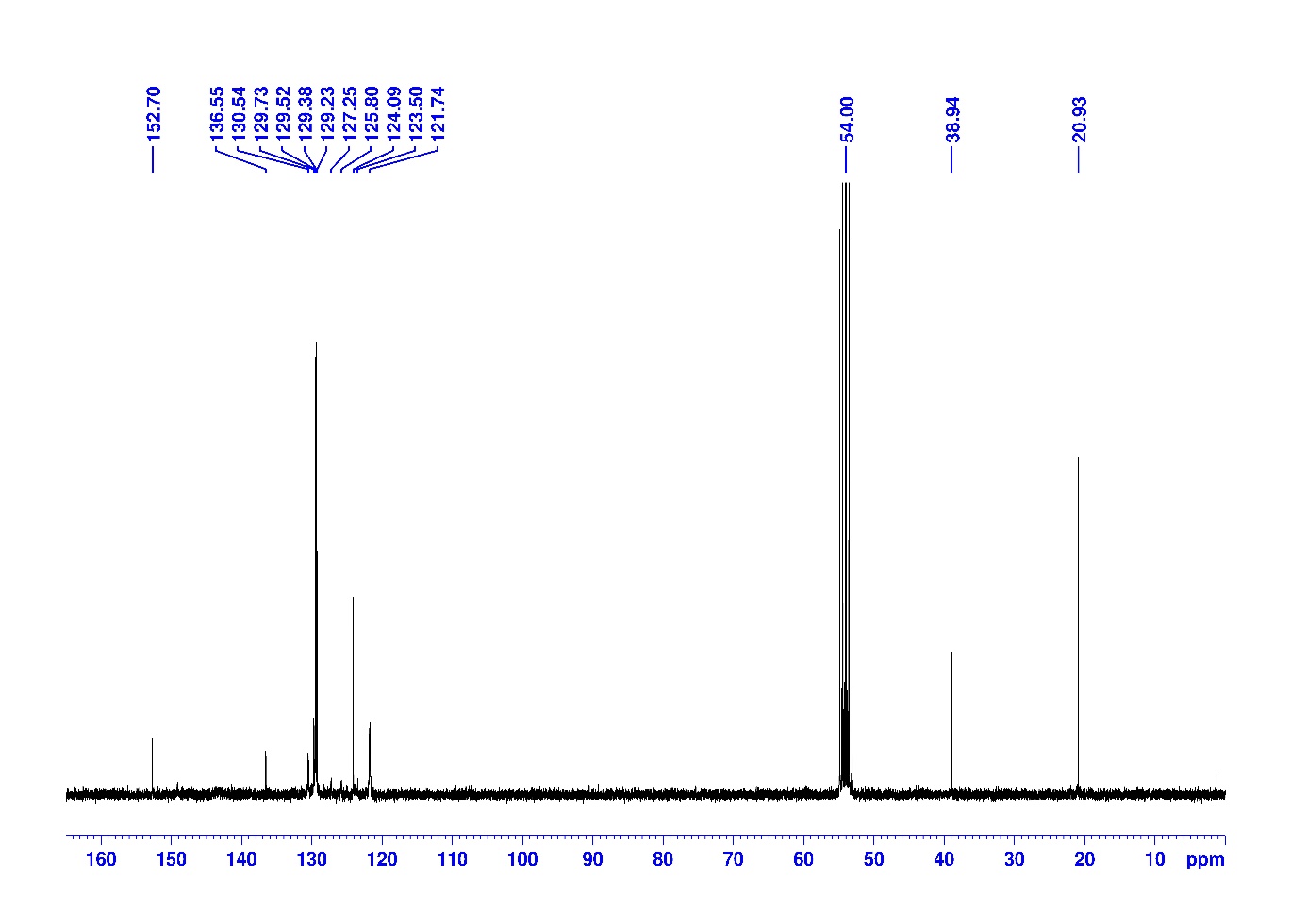


**Figure S8:** ^13^C{^1^H}-NMR-Spectrum of **PhMII-PhCDI** (CD_2_Cl_2_, 63 MHz).


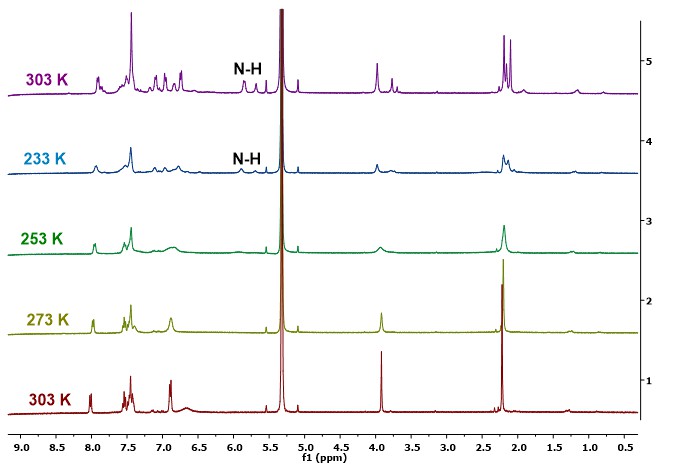


**Figure S9:** Variation temperature ^1^H-NMR-Spectrum of **PhMII-TolCDI** (CD_2_Cl_2_, 400 MHz).


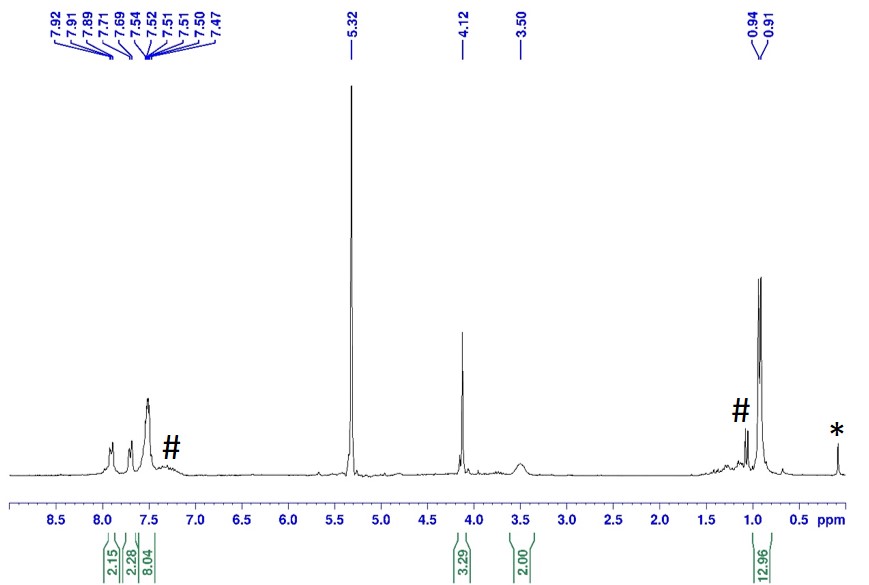


**Figure S10:** ^1^H-NMR-Spectrum of **PhMII-iPrCDI** (CD_2_Cl_2_, 250 MHz). Hashtags (#) annotate some inseparable impurities.


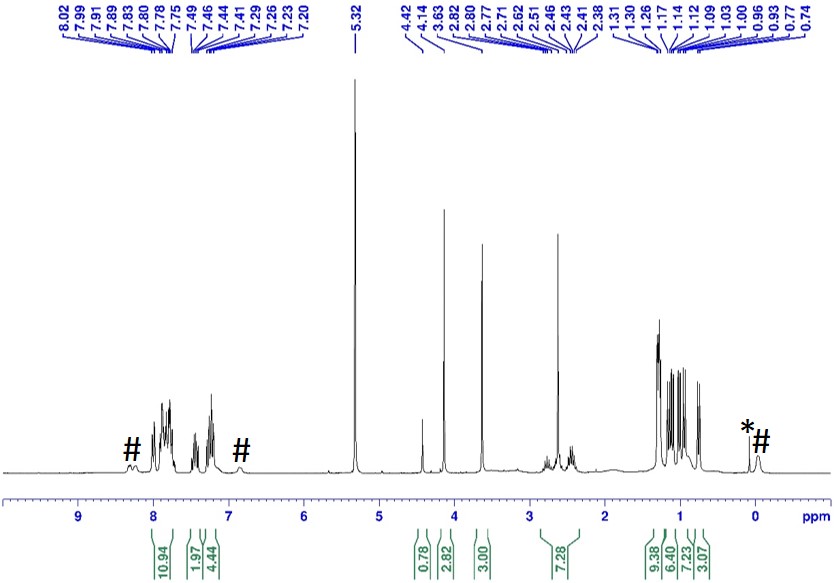


**Figure S11:** ^1^H-NMR-Spectrum of **Dipp-N(I)HOTf** (CD_2_Cl_2_, 250 MHz). Hashtags (#) annotate some inseparable impurities.


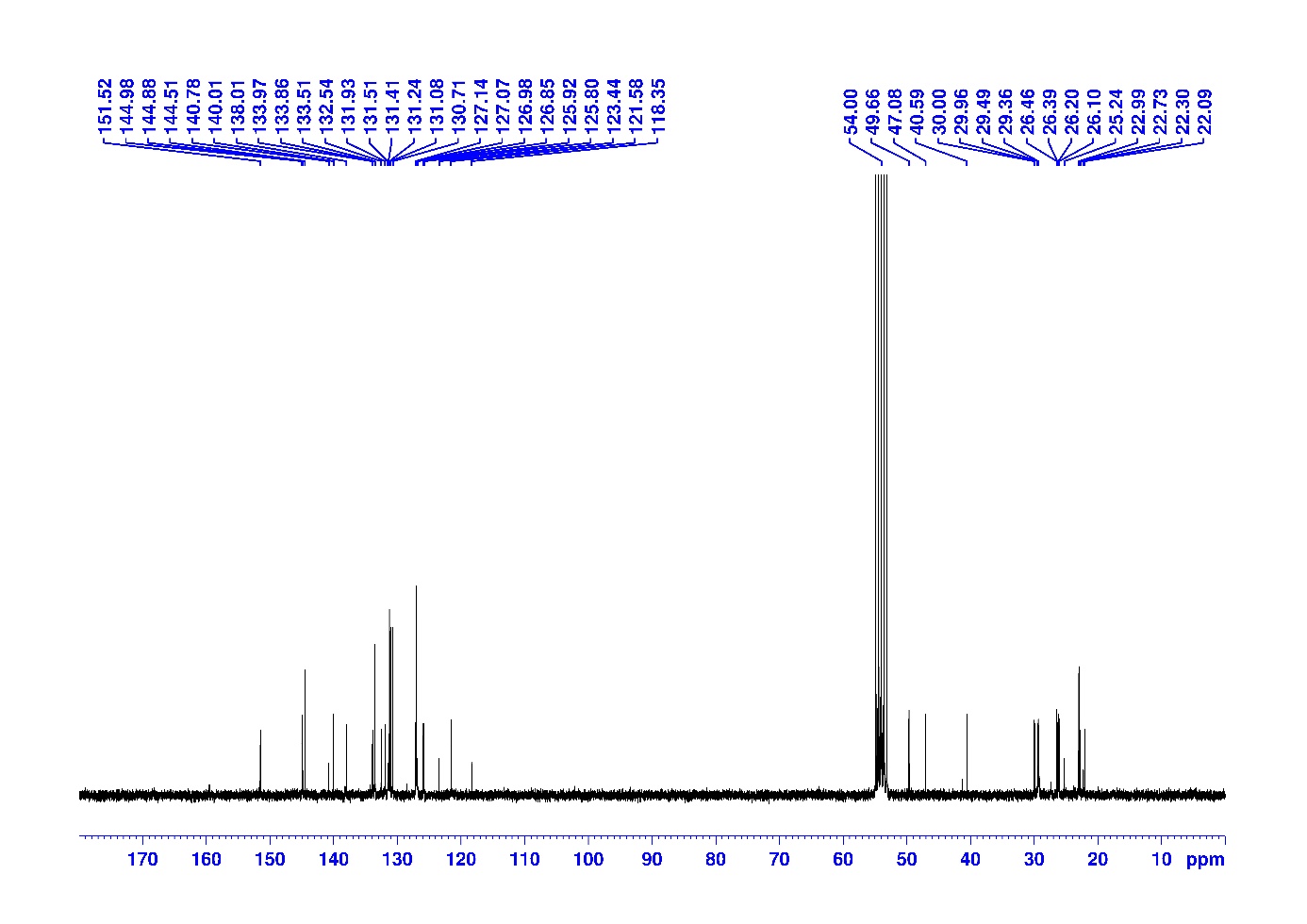


**Figure S12:** ^13^C{^1^H}-NMR-Spectrum **Dipp-N(I)HOTf** (CD_2_Cl_2_, 63 MHz).


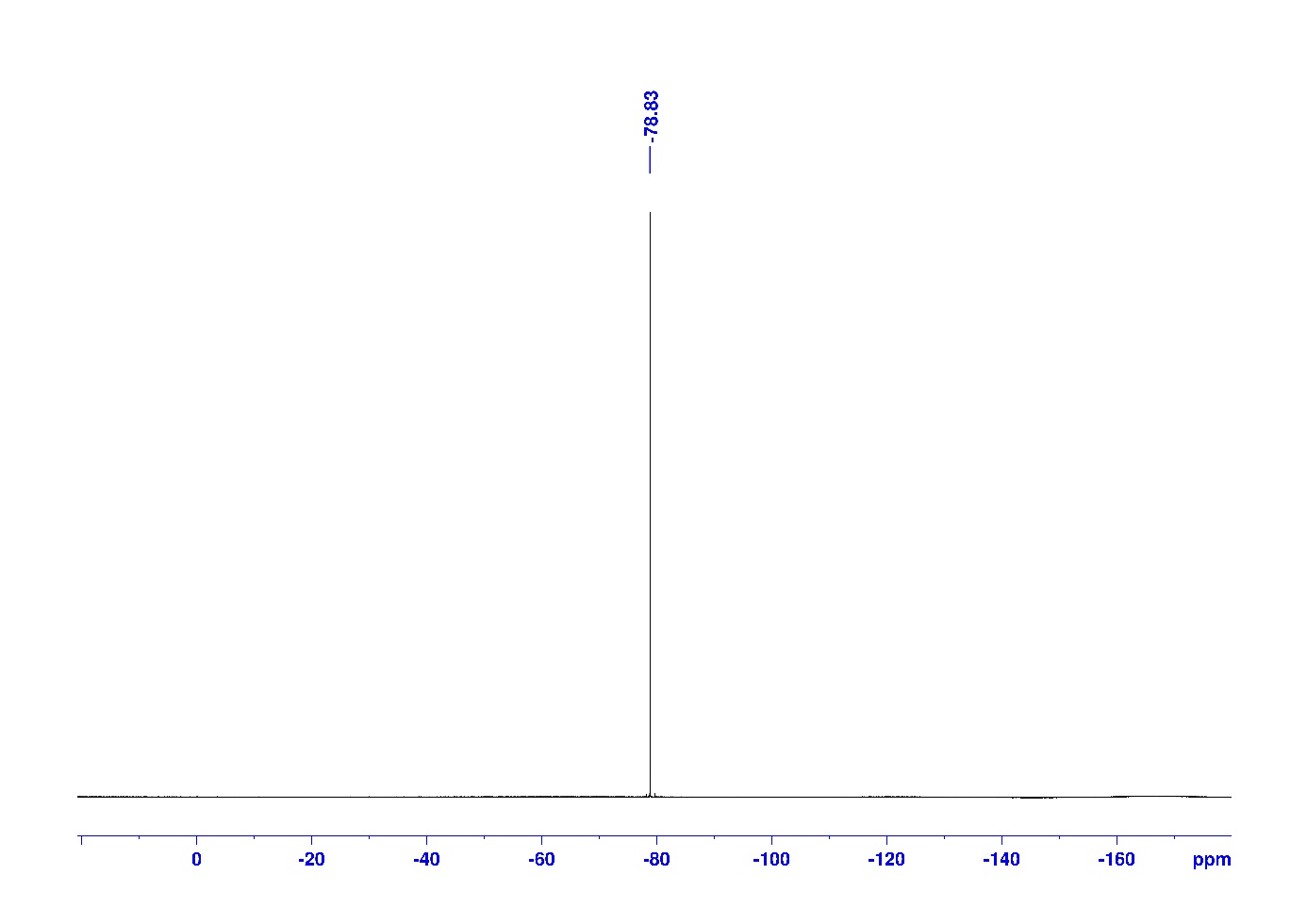


**Figure S13:** ^19^F-NMR-Spectrum of **N(I)HOTf** (CD_2_Cl_2_, 235 MHz).


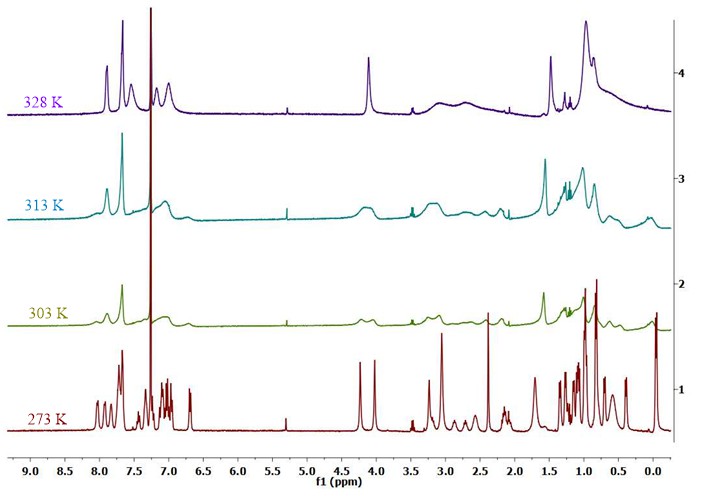


**Figure S14:** Variation temperature ^1^H-NMR-Spectrum of **N(I)** (CDCl_3_, 400 MHz).


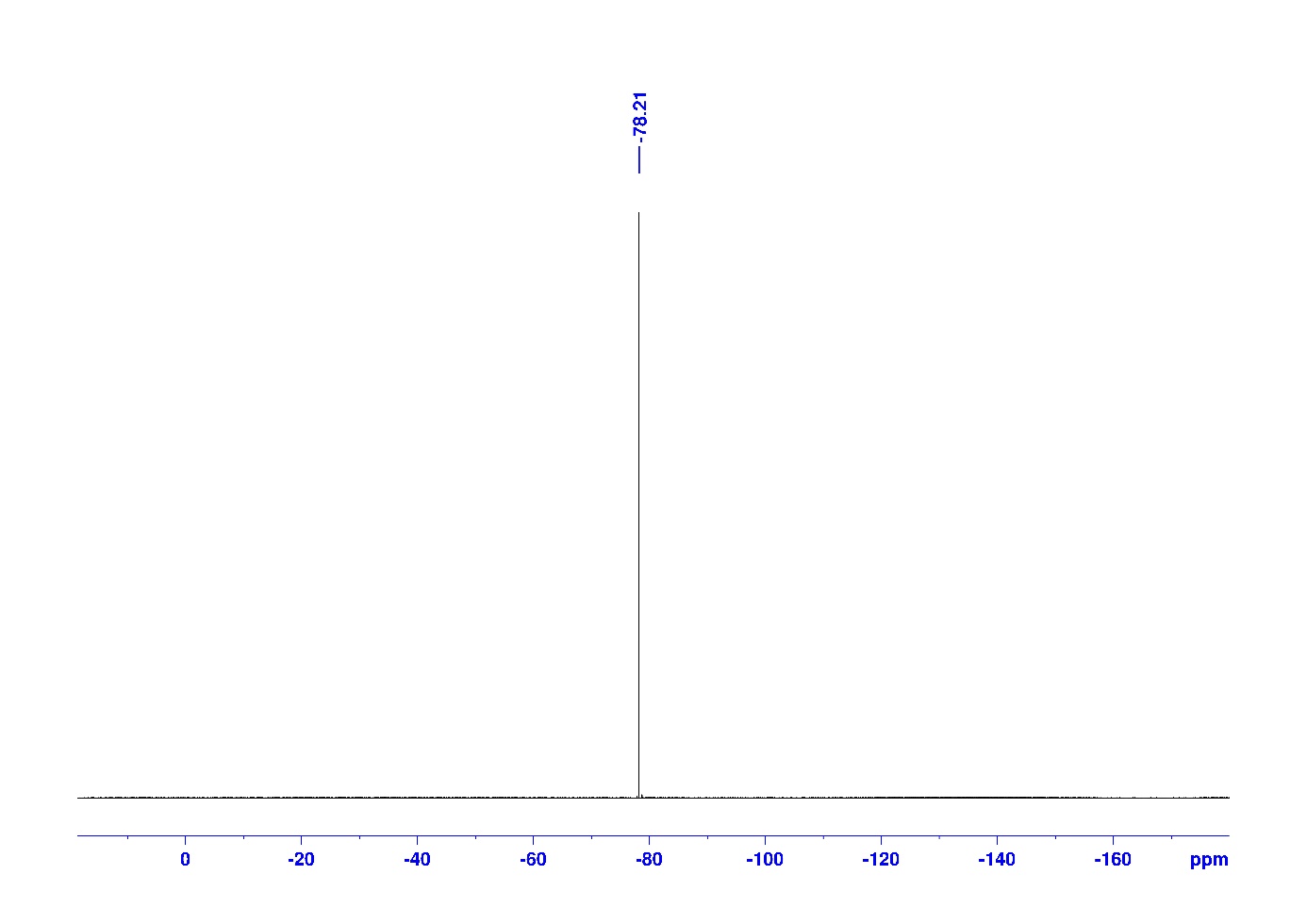


**Figure S15:** ^19^F-NMR-Spectrum of **N(I)** (CDCl_3_, 376 MHz).


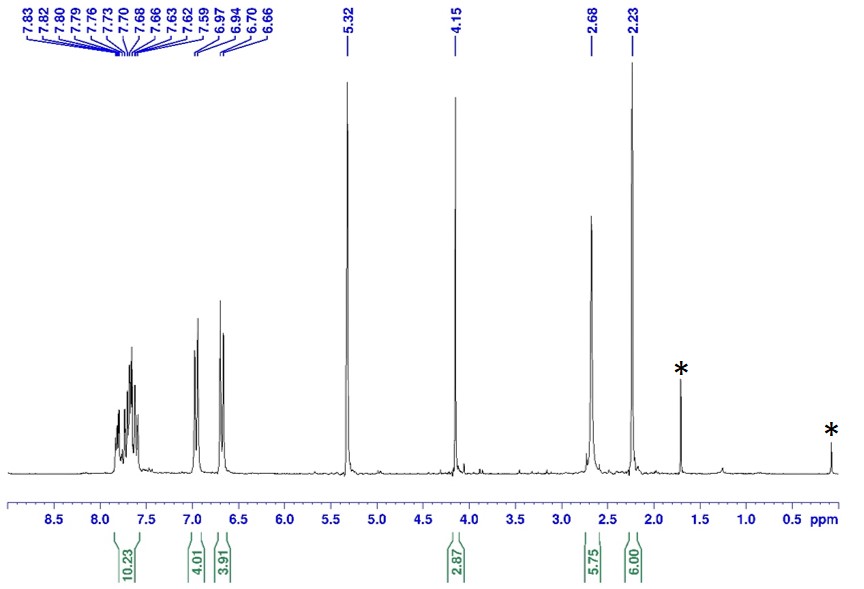


**Figure S16:** ^1^H-NMR-Spectrum of **Tol-N(I)** (CD_2_Cl_2_, 250 MHz).


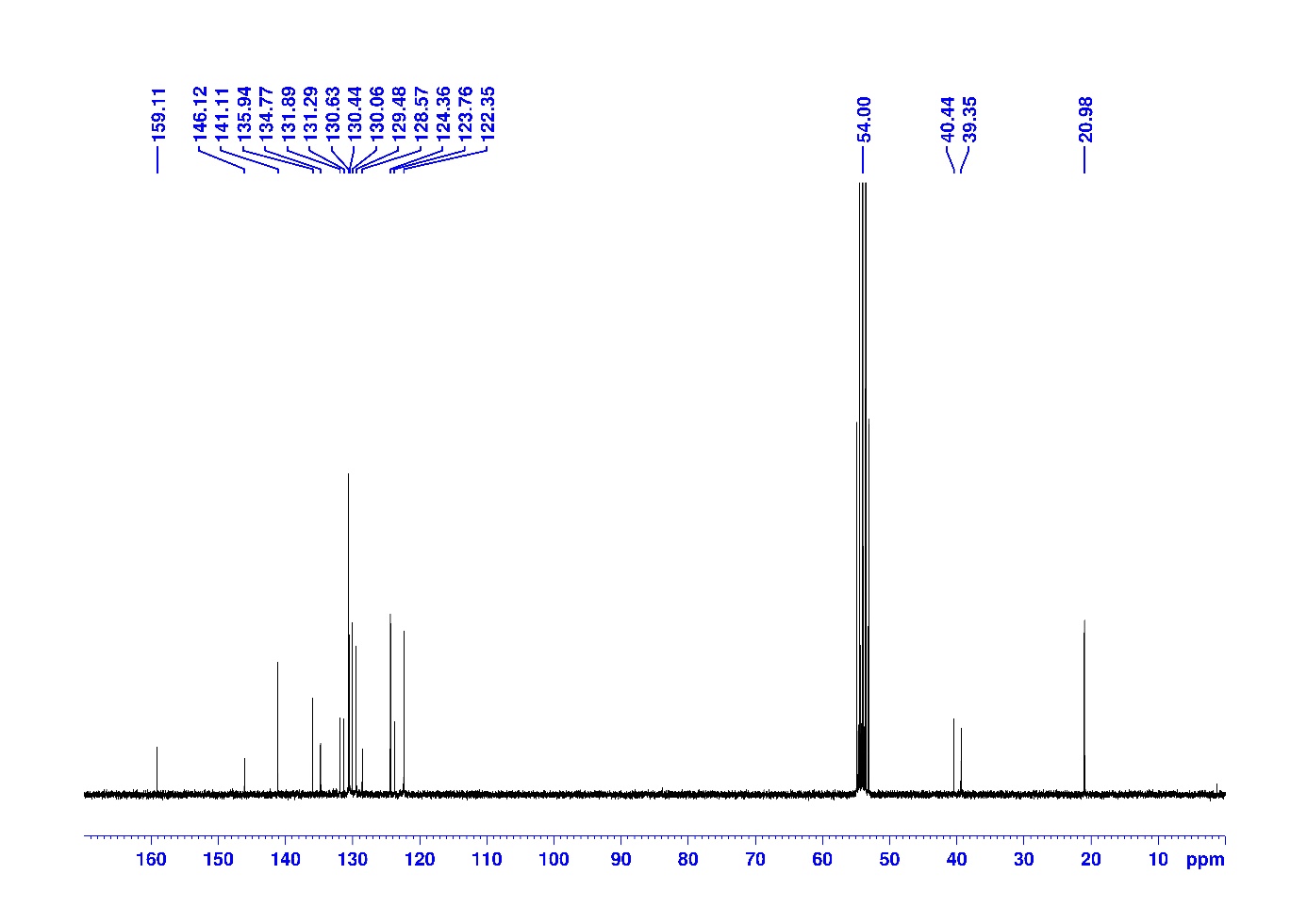


**Figure S17:** ^13^C{^1^H}-NMR-Spectrum **Tol-N(I)** (CD_2_Cl_2_, 63 MHz).


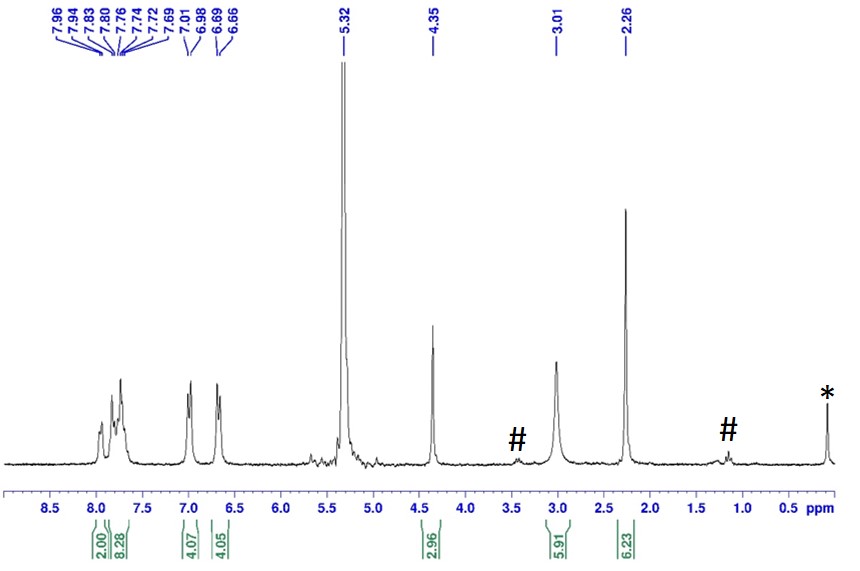


**Figure S18:** ^1^H-NMR-Spectrum of **Tol-N(I)HOTf** (CD_2_Cl_2_, 250 MHz). Hashtags (#) annotate signal of Et_2_O.


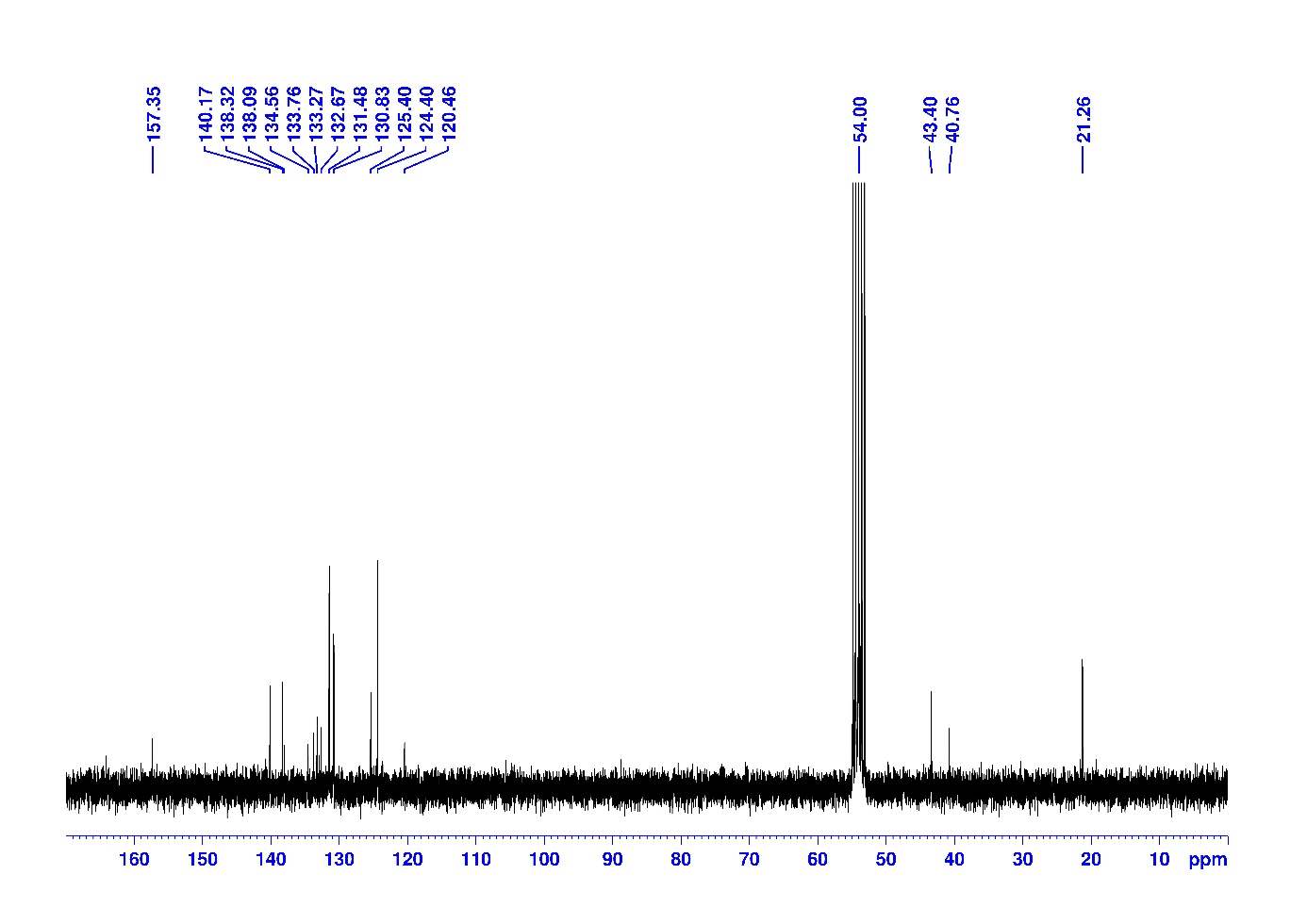


**Figure S19:** ^13^C{^1^H}-NMR-Spectrum **Tol-N(I)** (CD_2_Cl_2_, 63 MHz).


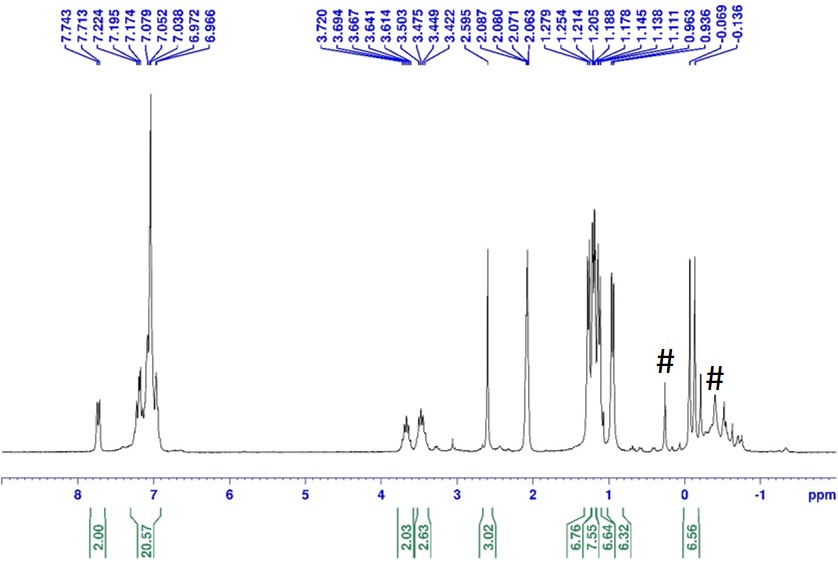


**Figure S20:** ^1^H-NMR-Spectrum of **PhMII-DippCDI-Al** (Tol-d_8_, 250 MHz). Hashtags (#) annotate some inseparable impurities form AlMe_3_ residue.


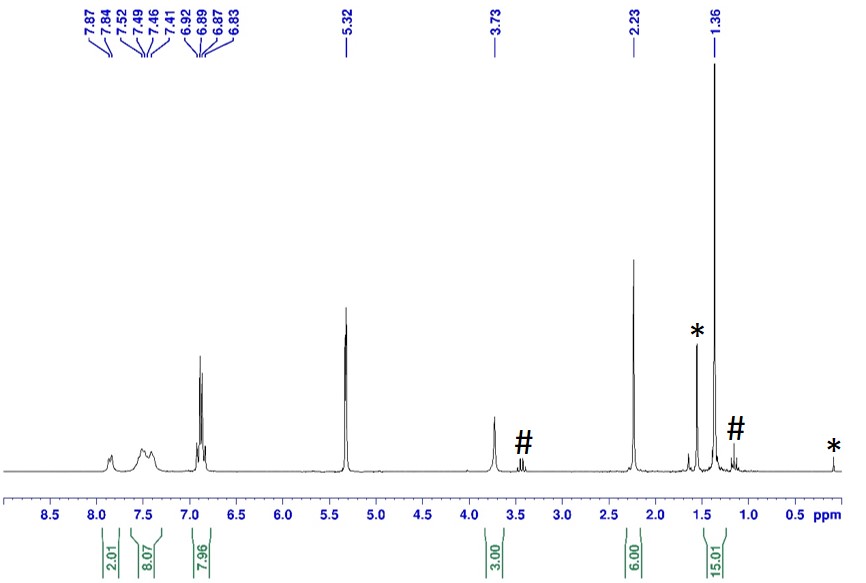


**Figure S21:** ^1^H-NMR-Spectrum of **PhMII-TolCDI-Rh** (CD_2_Cl_2_, 250 MHz). Hashtags (#) annotate signal of Et_2_O.


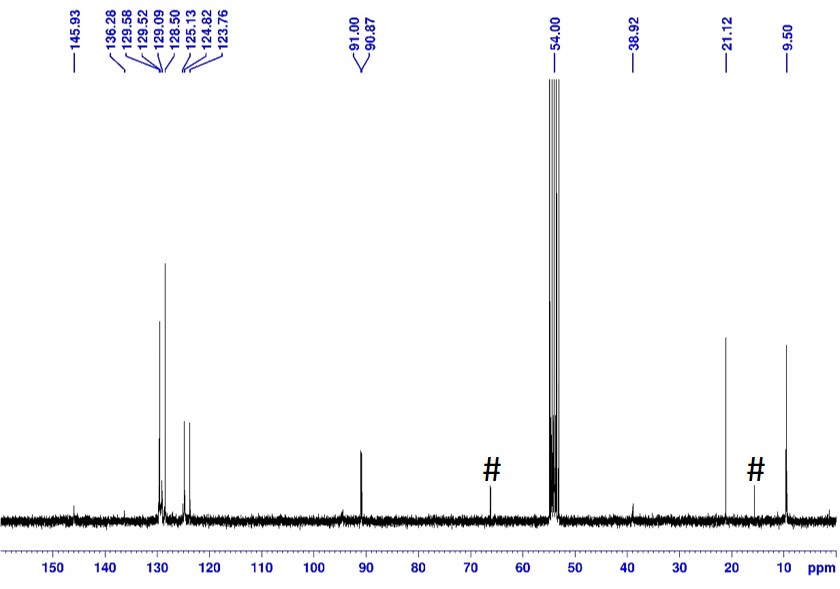


**Figure S22:** ^13^C{^1^H}-NMR-Spectrum **PhMII-TolCDI-Rh** (CD_2_Cl_2_, 63 MHz). Hashtags (#) annotate signal of Et_2_O.

**4. NMR Spectroscopy controlled reactions**

Reaction of **Dipp-N(I)** with AlCl_3_ for the synthesis of **Dipp-N(I)AlCl_3_**

**Dipp-N(I)** (9 mg, 0.01 mmol, 1 equiv.) and AlCl_3_ (5 mg, 0.04 mmol, 4 equiv.) was placed into an J Young NMR tube and 0.5 mL of CD_2_Cl_2_ was added to it. A sharp change in the ^1^H NMR spectra could be observed and after 2 days clean conversion to a new product could be seen. As the trifluoromethsne sulfonate counter anion could also can form adduct with AlCl_3_ we are not sure about the counter anion present in the molecule. This reaction was also performed in bulk scale, and a similar product was obtained.


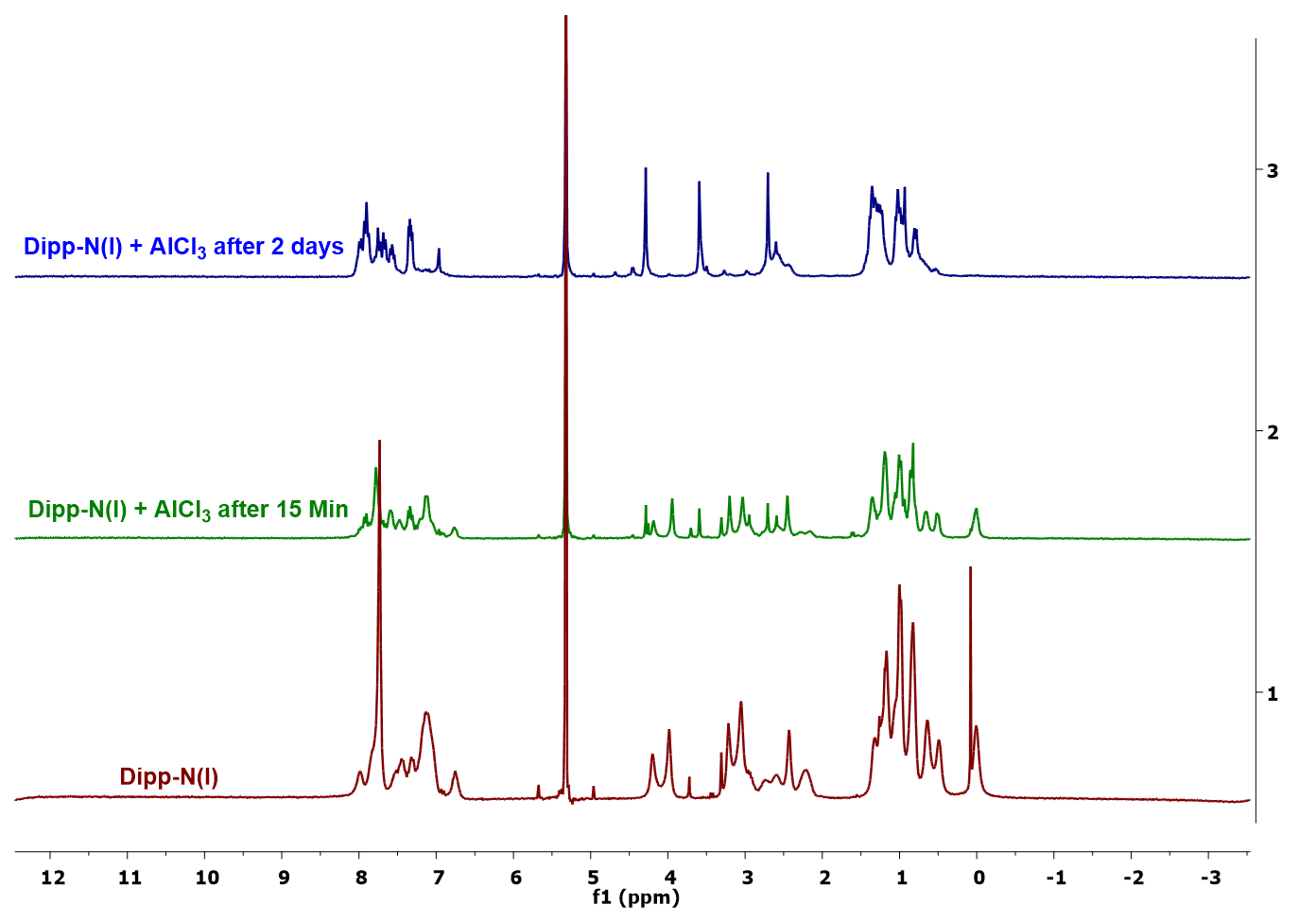


**Figure S23:** change in the ^1^H-NMR-Spectra of **Dipp-N(I)** after reaction with AlCl_3_ (CD_2_Cl_2_, 250 MHz).

Reaction of **Tol-N(I)** with AlCl_3_ for the synthesis of **Tol-N(I)AlCl_3_**

**Tol-N(I)** (6 mg, 0.01 mmol, 1 equiv.) and AlCl_3_ (5 mg, 0.04 mmol, 4 equiv.) was placed into an J Young NMR tube and 0.5 mL of CD_2_Cl_2_ was added to it. In this case also a sharp change in the ^1^H NMR spectra could be observed and after 2 days clean conversion to a new product could be seen. Downfield shift of all the peaks suggests the decrease in electron density in the molecule and hence formation of the complex. As the iodide counter anion could also can form adduct with AlCl_3_ we are not sure about the counter anion present in the molecule. This reaction was also performed in bulk scale, and a similar product was obtained.


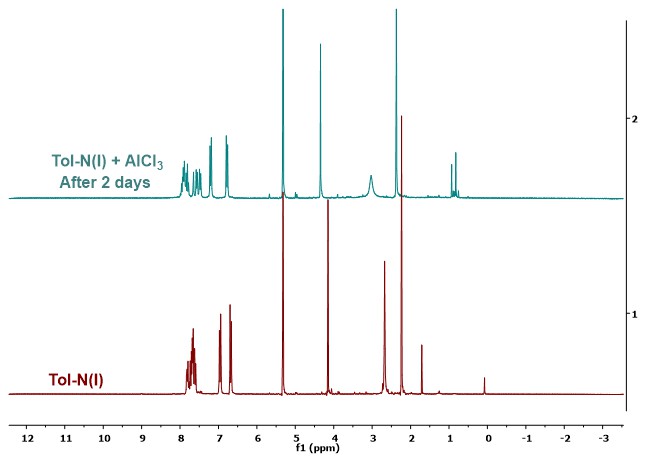


**Figure S24:** change in the ^1^H-NMR-Spectra of **Tol-N(I)** after reaction with AlCl_3_ (CD_2_Cl_2_, 250 MHz).

Deprotation reaction of **PhMII-DippCDI** with KH for the synthesis of **PhMII-DippCDI-K**

The adduct **PhMII-DippCDI** (6 mg, 0.01 mmol, 1 equiv.) and KH (4 mg, 0.05 mmol, 5 equiv.) was placed into a J Young NMR tube and dissolved in dry THF-d_8_ (0.5 mL). The NMR tube was heated at 60 °C for overnight. The color of the solution changed to light orange from yellow and H_2_ gas was formed. The ^1^H NMR spectra shows disappearance of the N-H signal and formation of characterstic H_2_ signal. Upfield shifting of all the aromatic protons suggests formation of negative charge in the molecule.


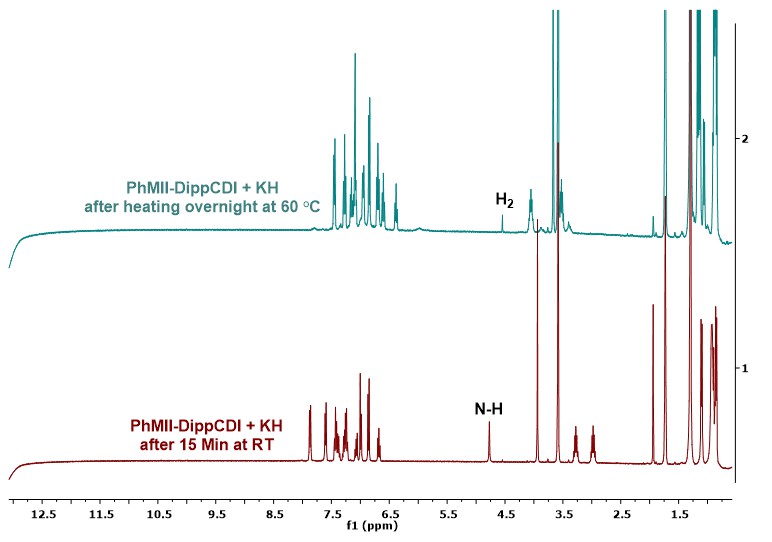


**Figure S25:** change in the ^1^H-NMR-Spectra of **PhMII-DippCDI** after reaction with KH (THF-d_8_, 400 MHz).

Deprotation reaction of **PhMII-TolCDI** with KH for the synthesis of **PhMII-TolCDI-K**

The adduct **PhMII-TolCDI** (5 mg, 0.01 mmol, 1 equiv.) and KH (4 mg, 0.05 mmol, 5 equiv.) was placed into a J Young NMR tube and dissolved in dry THF-d_8_ (0.5 mL). The NMR tube was heated at 60 °C for overnight. The color of the solution changed to light orange from yellow and H_2_ gas was formed. The ^1^H NMR spectra shows formation of characterstic H_2_ signal. In this case also upfield shifting of all the aromatic protons was observed, which suggests formation of negative charge in the molecule.


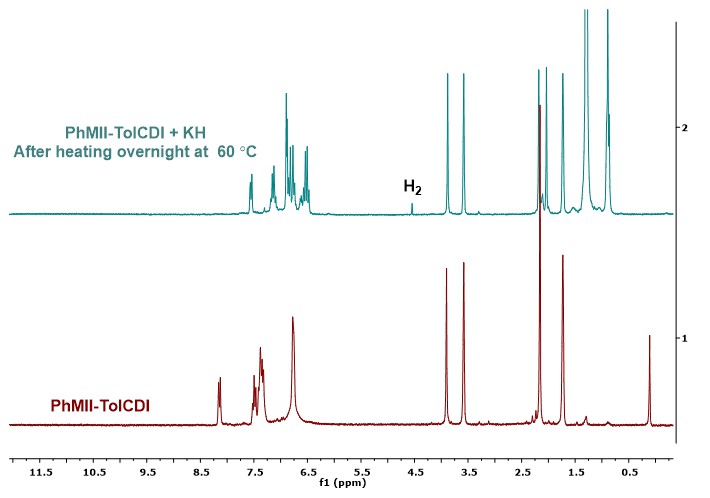


**Figure S26:** change in the ^1^H-NMR-Spectra of **PhMII-TolCDI** after reaction with KH (THF-d_8_, 250 MHz).

Deprotation reaction of **PhMII-TolCDI** with NaHMDS for the synthesis of **PhMII-TolCDI-Na**

The adduct **PhMII-TolCDI** (5 mg, 0.01 mmol, 1 equiv.) and NaHMDS (9 mg, 0.05 mmol, 5 equiv.) was placed into a J Young NMR tube and dissolved in dry THF-d_8_ (0.5 mL). The NMR tube was heated at 60 °C for 3 days. The color of the solution changed to light orange from yellow. In this case also upfield shifting of all the aromatic protons was observed, which suggests formation of negative charge in the molecule and formation of similar type of guanidinate-Na salt.


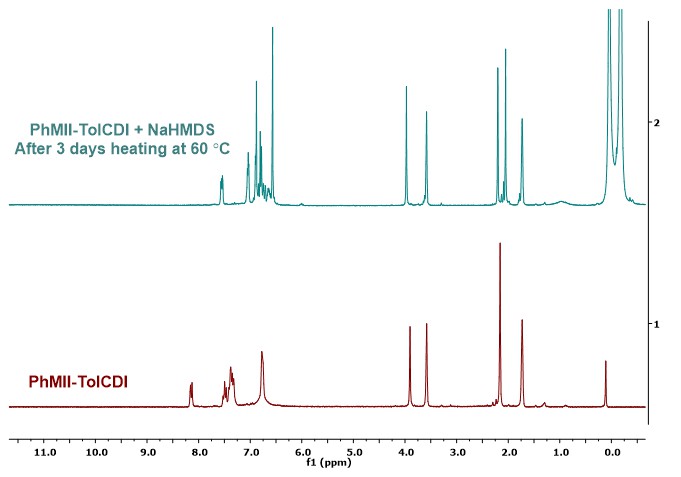


**Figure S27:** change in the ^1^H-NMR-Spectra of **PhMII-TolCDI** after reaction with NaHMDS (THF-d_8_, 250 MHz).

**5. Crystal structures and crystallographic data**

Crystal data and structure refinement of **MesMII-DippCDI**(CCDC #2375078)


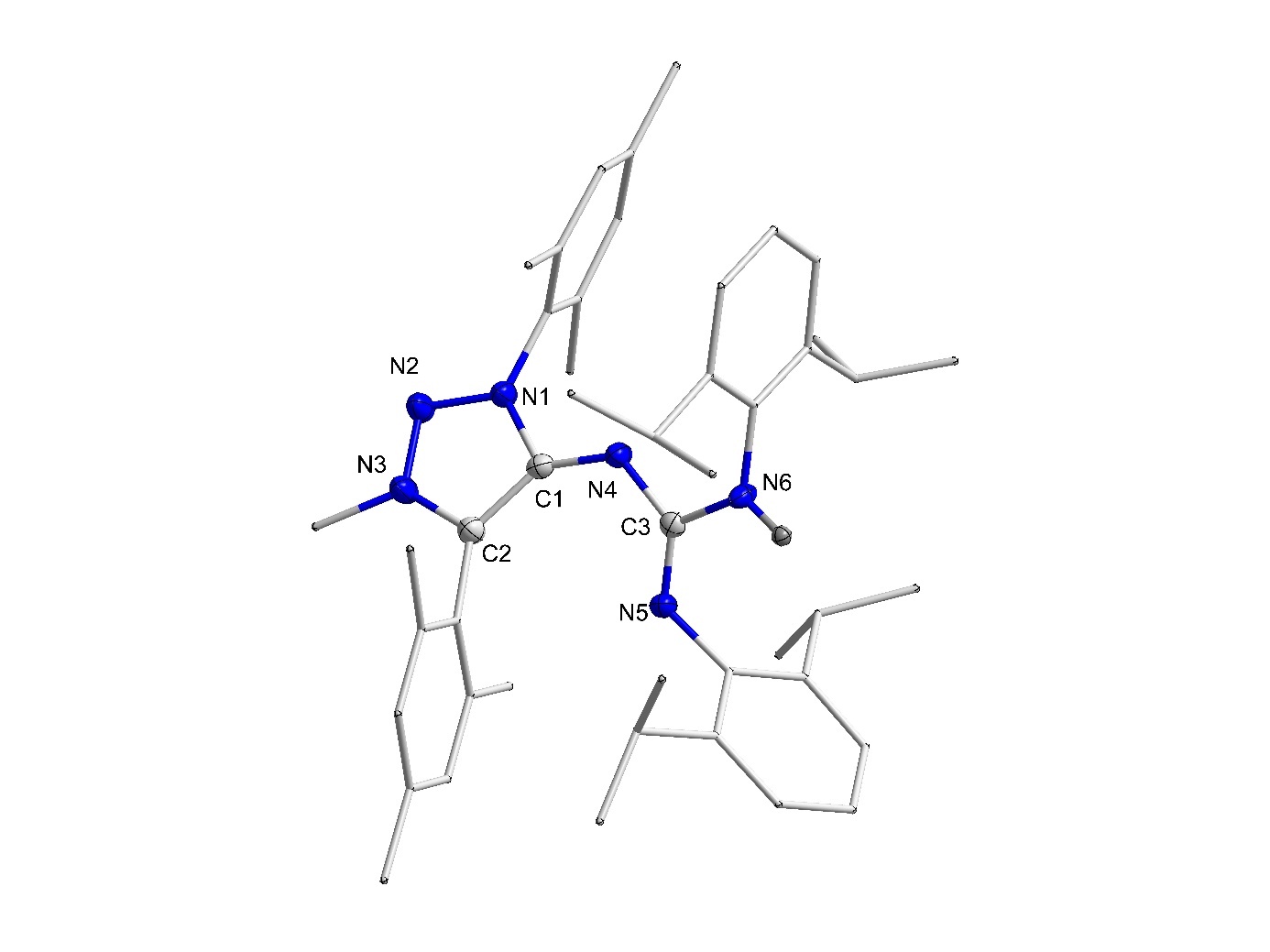


**Figure S28:** XRay solid-state structure of **MesMII-DippCDI** Ellipsoids are all set to 50% probability.

| **Table S1:** Crystal data and structure refinement for **MesMII-DippCDI**. | |
| --- | --- |
| Identification code | **MesMII-DippCDI** |
| Empirical formula | C_46_H_60_N_6_ |
| Formula weight | 697.00 |
| Temperature/K | 149.99 |
| Crystal system | triclinic |
| Space group | P-1 |
| a/Å | 11.3096(4) |
| b/Å | 11.9656(5) |
| c/Å | 17.3025(9) |
| α/° | 96.980(3) |
| β/° | 97.439(3) |
| γ/° | 115.997(2) |
| Volume/Å^3^ | 2044.67(16) |
| Z | 2 |
| ρ_calc_g/cm^3^ | 1.132 |
| μ/mm^‑1^ | 0.067 |
| F(000) | 756.0 |
| Crystal size/mm^3^ | 0.599 × 0.428 × 0.244 |
| Radiation | MoKα (λ = 0.71073) |
| 2Θ range for data collection/° | 3.866 to 56.69 |
| Index ranges | -15 ≤ h ≤ 15, -15 ≤ k ≤ 15, -23 ≤ l ≤ 23 |
| Reflections collected | 35380 |
| Independent reflections | 10149 [R_int_ = 0.0369, R_sigma_ = 0.0467] |
| Data/restraints/parameters | 10149/0/484 |
| Goodness-of-fit on F^2^ | 1.025 |
| Final R indexes [I>=2σ (I)] | R_1_ = 0.0521, wR_2_ = 0.1217 |
| Final R indexes [all data] | R_1_ = 0.0829, wR_2_ = 0.1391 |
| Largest diff. peak/hole / e Å^-3^ | 0.34/-0.34 |

Crystal data and structure refinement of **PhMII-DippCDI** (CCDC #2375077)


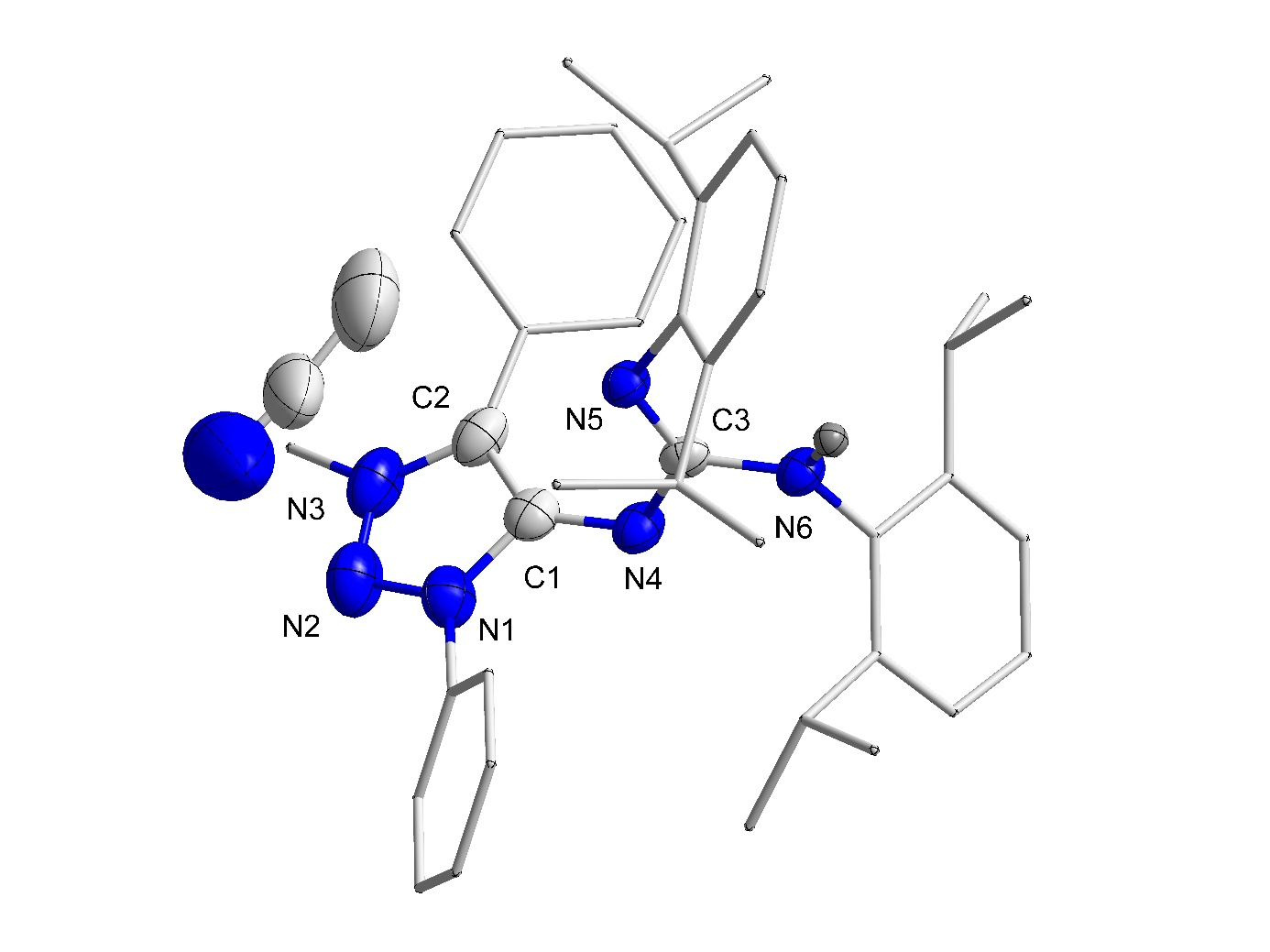


**Figure S29:** XRay solid-state structure of **PhMII-DippCDI**.CH_3_CN Ellipsoids are all set to 50% probability.

| **Table S2:** Crystal data and structure refinement for **PhMII-DippCDI**. | |
| --- | --- |
| Identification code | **PhMII-DippCDI** |
| Empirical formula | C_42_H_51_N_7_ |
| Formula weight | 653.89 |
| Temperature/K | 296.15 |
| Crystal system | triclinic |
| Space group | P-1 |
| a/Å | 10.9815(5) |
| b/Å | 11.6460(5) |
| c/Å | 17.0073(6) |
| α/° | 75.370(2) |
| β/° | 88.863(2) |
| γ/° | 65.649(2) |
| Volume/Å^3^ | 1908.54(14) |
| Z | 2 |
| ρ_calc_g/cm^3^ | 1.138 |
| μ/mm^‑1^ | 0.068 |
| F(000) | 704.0 |
| Crystal size/mm^3^ | 0.454 × 0.431 × 0.344 |
| Radiation | MoKα (λ = 0.71073) |
| 2Θ range for data collection/° | 3.984 to 52.832 |
| Index ranges | -13 ≤ h ≤ 13, -14 ≤ k ≤ 14, -21 ≤ l ≤ 21 |
| Reflections collected | 27471 |
| Independent reflections | 7822 [R_int_ = 0.0176, R_sigma_ = 0.0235] |
| Data/restraints/parameters | 7822/0/452 |
| Goodness-of-fit on F^2^ | 1.044 |
| Final R indexes [I>=2σ (I)] | R_1_ = 0.0520, wR_2_ = 0.1402 |
| Final R indexes [all data] | R_1_ = 0.0789, wR_2_ = 0.1587 |
| Largest diff. peak/hole / e Å^-3^ | 0.38/-0.26 |

Crystal data and structure refinement of **N(I)** (CCDC # 2375076)


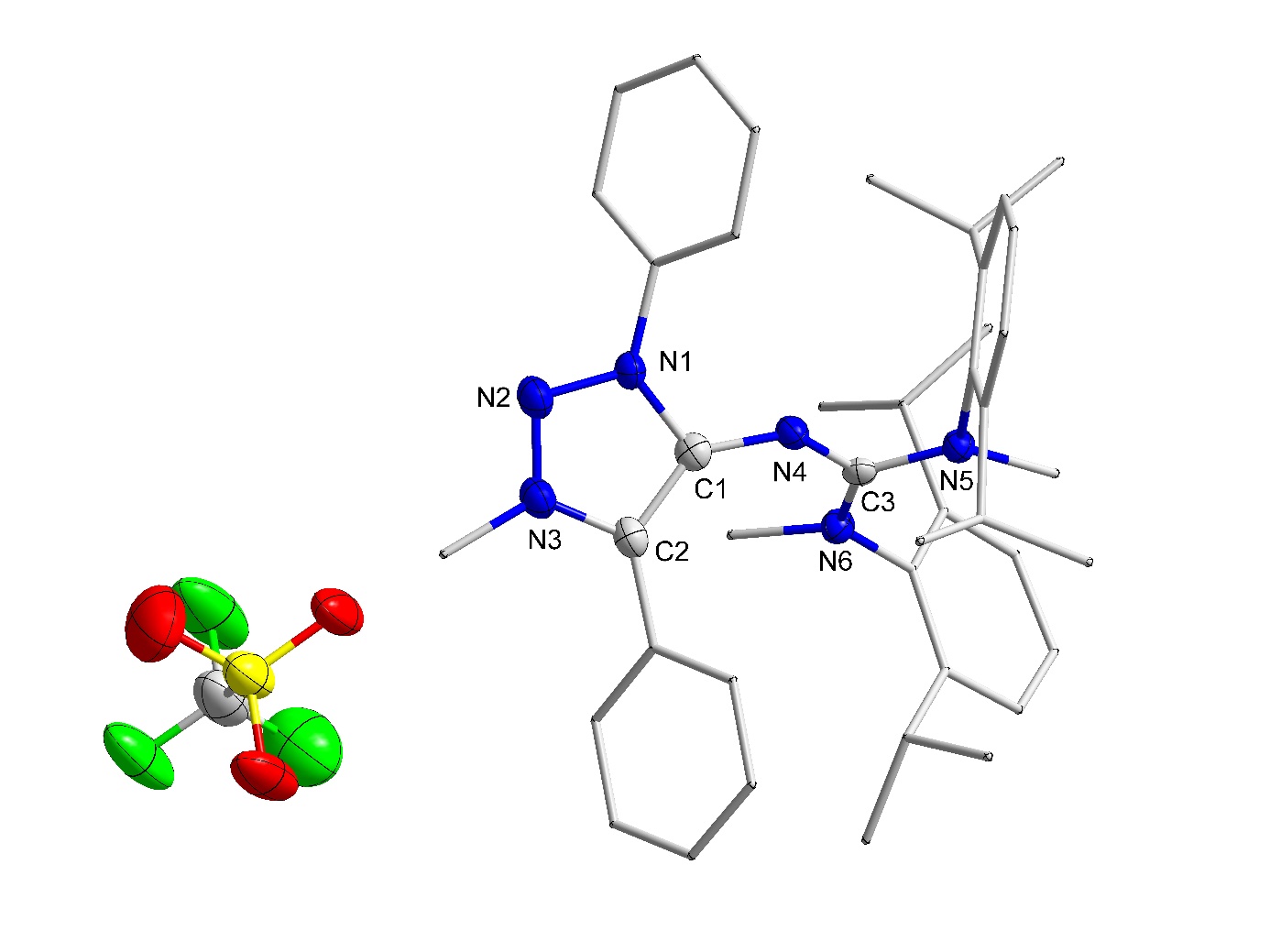


**Figure S30:** XRay solid-state structure of **N(I)**. Ellipsoids are all set to 50% probability.

| **Table S3:** Crystal data and structure refinement for **N(I)**. | |
| --- | --- |
| Identification code | **N(I)** |
| Empirical formula | C_43_H_53_F_3_N_6_O_3_S |
| Formula weight | 790.97 |
| Temperature/K | 150.01 |
| Crystal system | monoclinic |
| Space group | P2_1_/c |
| a/Å | 12.265(2) |
| b/Å | 32.567(5) |
| c/Å | 10.4610(18) |
| α/° | 90 |
| β/° | 100.634(6) |
| γ/° | 90 |
| Volume/Å^3^ | 4106.7(11) |
| Z | 4 |
| ρ_calc_g/cm^3^ | 1.279 |
| μ/mm^‑1^ | 0.139 |
| F(000) | 1680.0 |
| Crystal size/mm^3^ | 0.43 × 0.31 × 0.23 |
| Radiation | MoKα (λ = 0.71073) |
| 2Θ range for data collection/° | 3.378 to 52.81 |
| Index ranges | -14 ≤ h ≤ 15, -40 ≤ k ≤ 32, -13 ≤ l ≤ 12 |
| Reflections collected | 24053 |
| Independent reflections | 8294 [R_int_ = 0.0805, R_sigma_ = 0.0918] |
| Data/restraints/parameters | 8294/0/516 |
| Goodness-of-fit on F^2^ | 1.050 |
| Final R indexes [I>=2σ (I)] | R_1_ = 0.0665, wR_2_ = 0.1649 |
| Final R indexes [all data] | R_1_ = 0.1081, wR_2_ = 0.1882 |
| Largest diff. peak/hole / e Å^-3^ | 0.40/-0.51 |

Crystal data and structure refinement of **N(I)HOTf** (CCDC # 2375065)


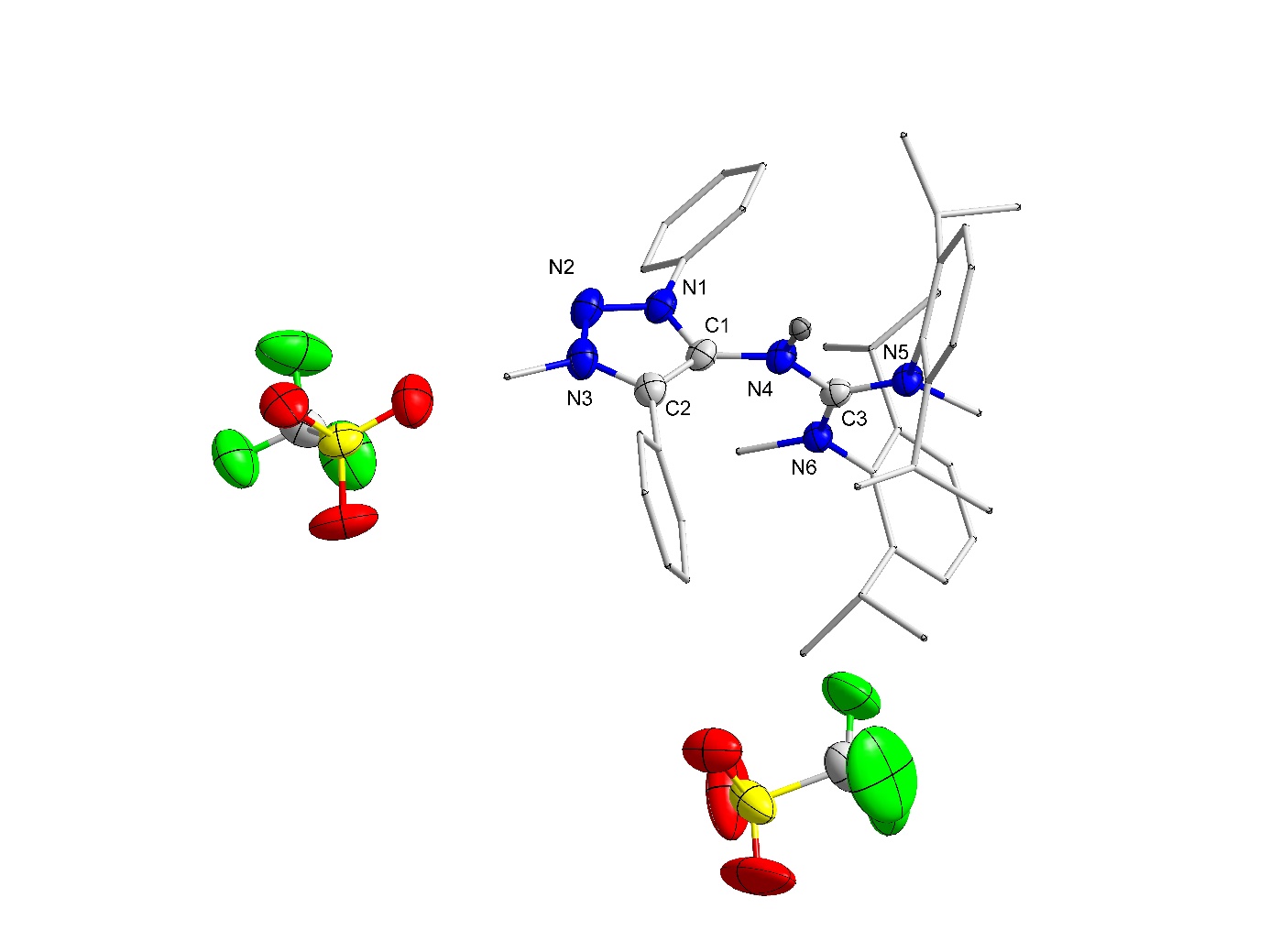


**Figure S31:** XRay solid-state structure of **N(I)HOTf**. Ellipsoids are all set to 50% probability.

| **Table S4:** Crystal data and structure refinement for **N(I)HOTf**. | |
| --- | --- |
| Identification code | **N(I)HOTf** |
| Empirical formula | C_44_H_54_F_6_N_6_O_6_S_2_ |
| Formula weight | 941.05 |
| Temperature/K | 150.00 |
| Crystal system | triclinic |
| Space group | P-1 |
| a/Å | 13.7499(7) |
| b/Å | 13.8457(6) |
| c/Å | 14.0582(7) |
| α/° | 85.903(2) |
| β/° | 74.112(2) |
| γ/° | 82.889(2) |
| Volume/Å^3^ | 2552.3(2) |
| Z | 2 |
| ρ_calc_g/cm^3^ | 1.224 |
| μ/mm^‑1^ | 0.174 |
| F(000) | 988.0 |
| Crystal size/mm^3^ | 0.87 × 0.58 × 0.52 |
| Radiation | MoKα (λ = 0.71073) |
| 2Θ range for data collection/° | 3.7 to 52.83 |
| Index ranges | -17 ≤ h ≤ 17, -16 ≤ k ≤ 17, -17 ≤ l ≤ 17 |
| Reflections collected | 34215 |
| Independent reflections | 10392 [R_int_ = 0.0288, R_sigma_ = 0.0311] |
| Data/restraints/parameters | 10392/0/588 |
| Goodness-of-fit on F^2^ | 1.047 |
| Final R indexes [I>=2σ (I)] | R_1_ = 0.0584, wR_2_ = 0.1601 |
| Final R indexes [all data] | R_1_ = 0.0799, wR_2_ = 0.1796 |
| Largest diff. peak/hole / e Å^-3^ | 0.62/-0.46 |

Crystal data and structure refinement of **PhMII-DippCDI-Al** (CCDC # 2388350)


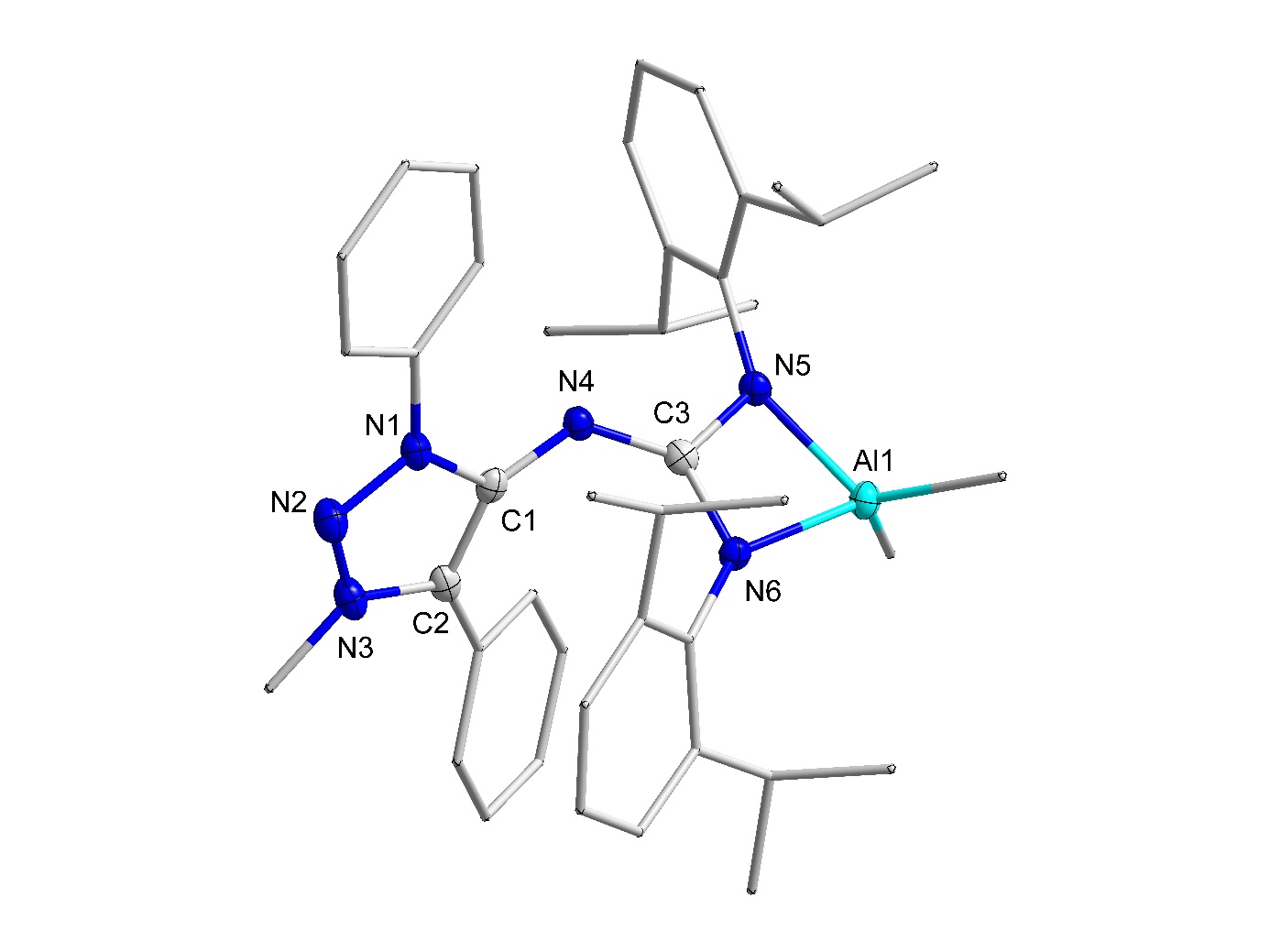


**Figure S32:** XRay solid-state structure of **PhMII-DippCDI-Al** Ellipsoids are all set to 50% probability.

| **Table S5:** Crystal data and structure refinement for **PhMII-DippCDI-Al**. | |
| --- | --- |
| Identification code | **PhMII-DippCDI-Al** |
| Empirical formula | C_42_H_53_AlN_6_ |
| Formula weight | 668.88 |
| Temperature/K | 99.99 |
| Crystal system | monoclinic |
| Space group | P2_1_/n |
| a/Å | 11.5707(5) |
| b/Å | 21.2510(7) |
| c/Å | 15.5709(6) |
| α/° | 90 |
| β/° | 96.2100(10) |
| γ/° | 90 |
| Volume/Å^3^ | 3806.2(3) |
| Z | 4 |
| ρ_calc_g/cm^3^ | 1.167 |
| μ/mm^‑1^ | 0.091 |
| F(000) | 1440.0 |
| Crystal size/mm^3^ | 0.341 × 0.19 × 0.15 |
| Radiation | MoKα (λ = 0.71073) |
| 2Θ range for data collection/° | 3.834 to 52.9 |
| Index ranges | -14 ≤ h ≤ 14, -17 ≤ k ≤ 26, -16 ≤ l ≤ 19 |
| Reflections collected | 27914 |
| Independent reflections | 7793 [R_int_ = 0.0308, R_sigma_ = 0.0408] |
| Data/restraints/parameters | 7793/0/453 |
| Goodness-of-fit on F^2^ | 1.024 |
| Final R indexes [I>=2σ (I)] | R_1_ = 0.0417, wR_2_ = 0.0965 |
| Final R indexes [all data] | R_1_ = 0.0632, wR_2_ = 0.1059 |
| Largest diff. peak/hole / e Å^-3^ | 0.33/-0.26 |

Crystal data and structure refinement of **PhMII-TolCDI-Rh**.2DCM (CCDC # 2449570)


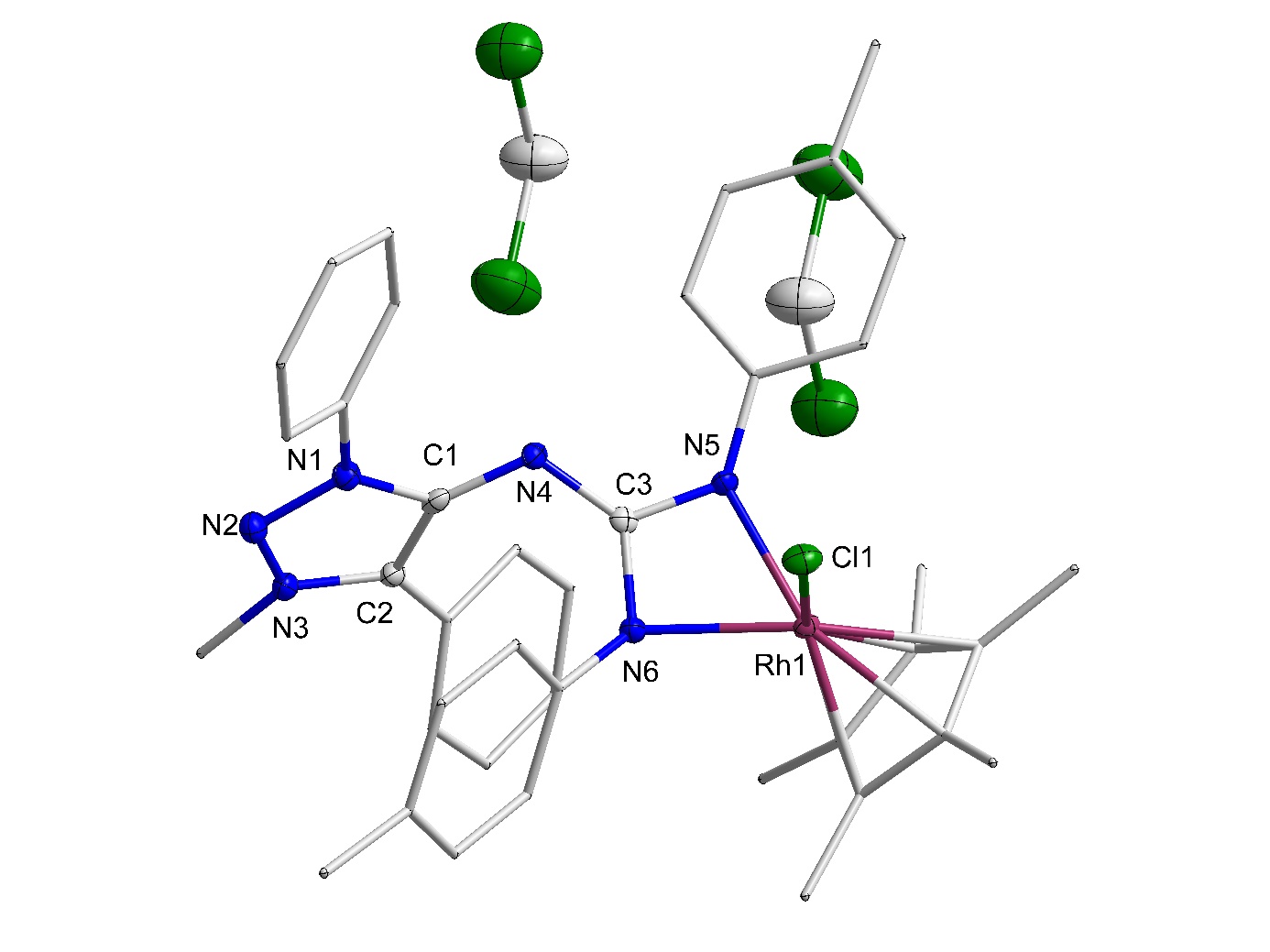


**Figure S33:** XRay solid-state structure of **PhMII-TolCDI-Rh**.2DCM Ellipsoids are all set to 50% probability.

| Table 1 Crystal data and structure refinement for **PhMII-TolCDI-Rh**. | |
| --- | --- |
| Identification code | **PhMII-TolCDI-Rh** |
| Empirical formula | C_42_H_46_Cl_5_N_6_Rh |
| Formula weight | 915.01 |
| Temperature/K | 99.99 |
| Crystal system | monoclinic |
| Space group | P2_1_/n |
| a/Å | 11.4225(9) |
| b/Å | 24.505(2) |
| c/Å | 15.5820(12) |
| α/° | 90 |
| β/° | 105.141(4) |
| γ/° | 90 |
| Volume/Å^3^ | 4210.1(6) |
| Z | 4 |
| ρ_calc_g/cm^3^ | 1.444 |
| μ/mm^‑1^ | 0.761 |
| F(000) | 1880.0 |
| Crystal size/mm^3^ | 0.42 × 0.25 × 0.16 |
| Radiation | MoKα (λ = 0.71073) |
| 2Θ range for data collection/° | 3.178 to 56.79 |
| Index ranges | -15 ≤ h ≤ 14, -32 ≤ k ≤ 32, -20 ≤ l ≤ 20 |
| Reflections collected | 37115 |
| Independent reflections | 10420 [R_int_ = 0.0399, R_sigma_ = 0.0512] |
| Data/restraints/parameters | 10420/0/495 |
| Goodness-of-fit on F^2^ | 1.057 |
| Final R indexes [I>=2σ (I)] | R_1_ = 0.0556, wR_2_ = 0.1395 |
| Final R indexes [all data] | R_1_ = 0.0778, wR_2_ = 0.1504 |
| Largest diff. peak/hole / e Å^-3^ | 1.65/-1.82 |


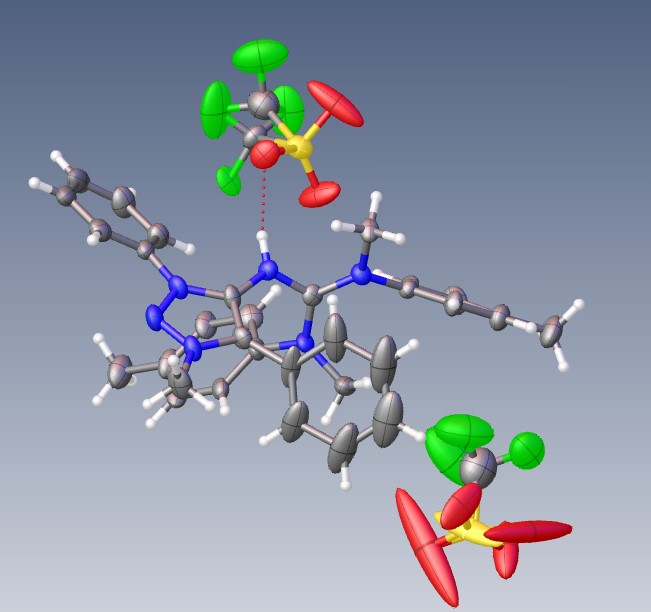


**Figure S34:** XRay solid-state structure of **Tol-N(I)HOTf**. Because of poor crystal quality, it has a lot of disorder. Thus we were unable to compare the bond parameters.

**6. Cyclic voltammetry, UV-Vis-NIR and EPR Spectro-electrochemistry**


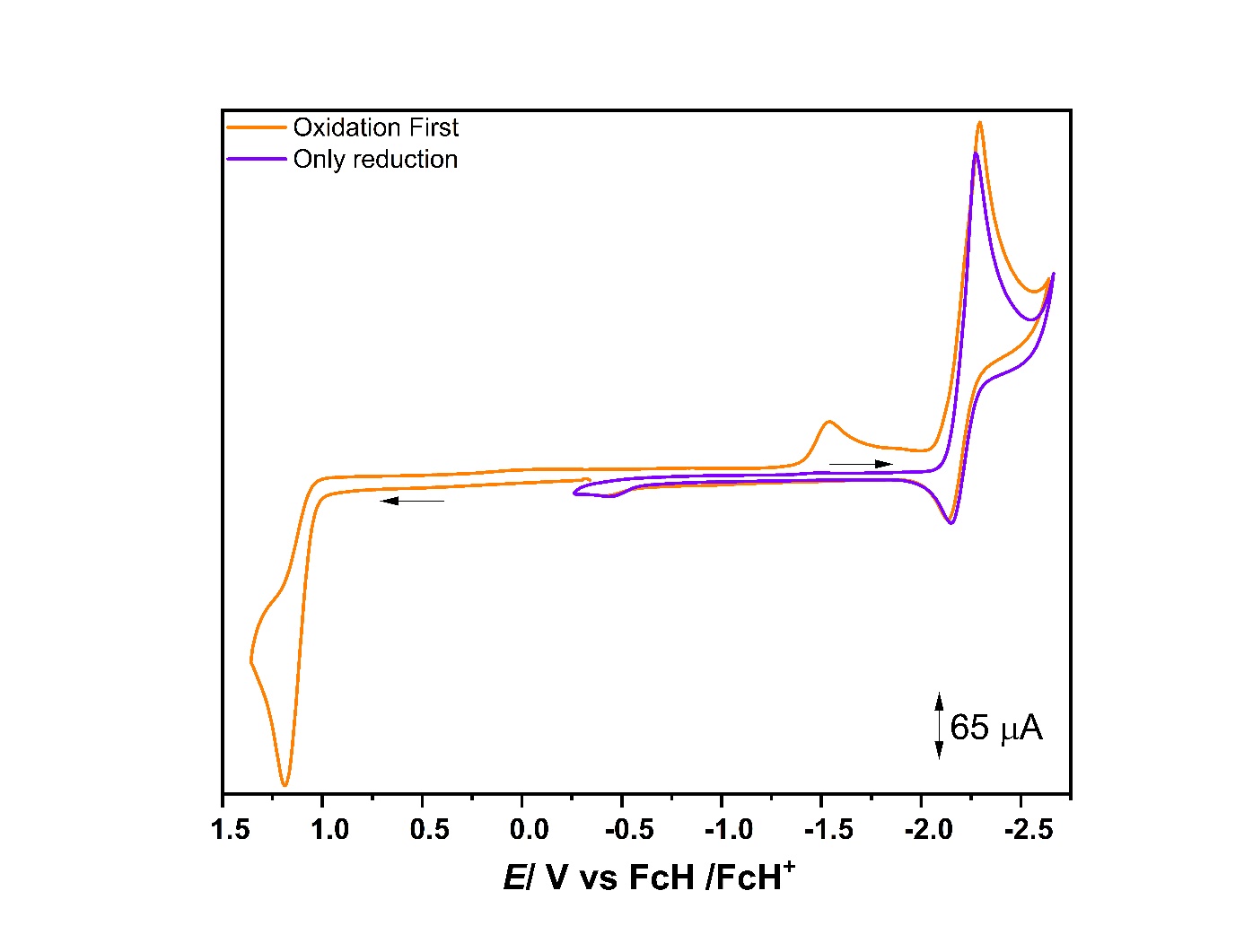


**Figure S35:** Cyclic voltammograms of a 0.1 mM solution of **N(I)** with 0.1 M NBu_4_PF_6_ in o- DFB at 100mV scan rate.


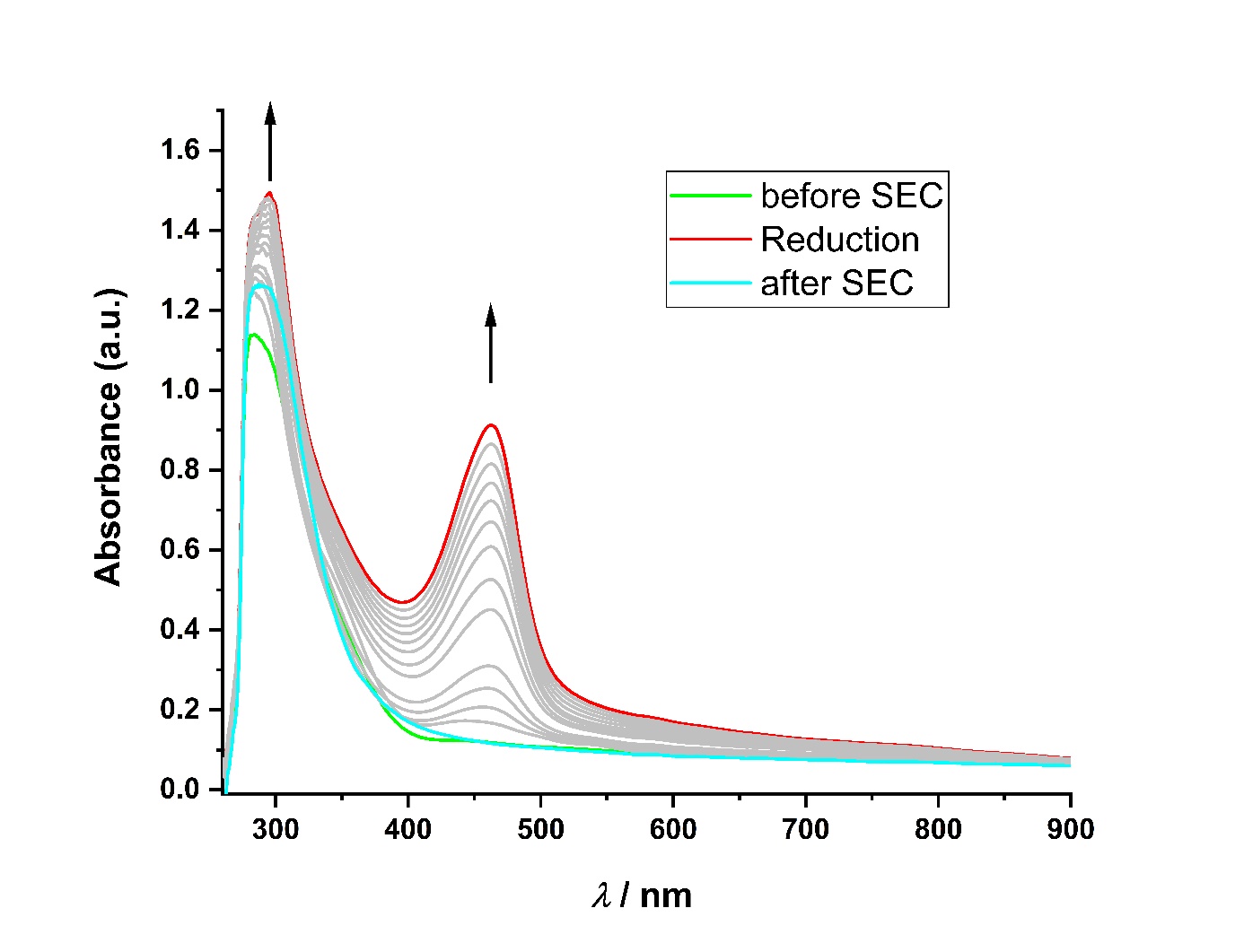


**Figure S36:** Changes in the UV/VIS/NIR spectra of **N(I)** in *o*-DFB/0.1M Bu_4_NPF_6_ during the reduction with an Au working electrode.


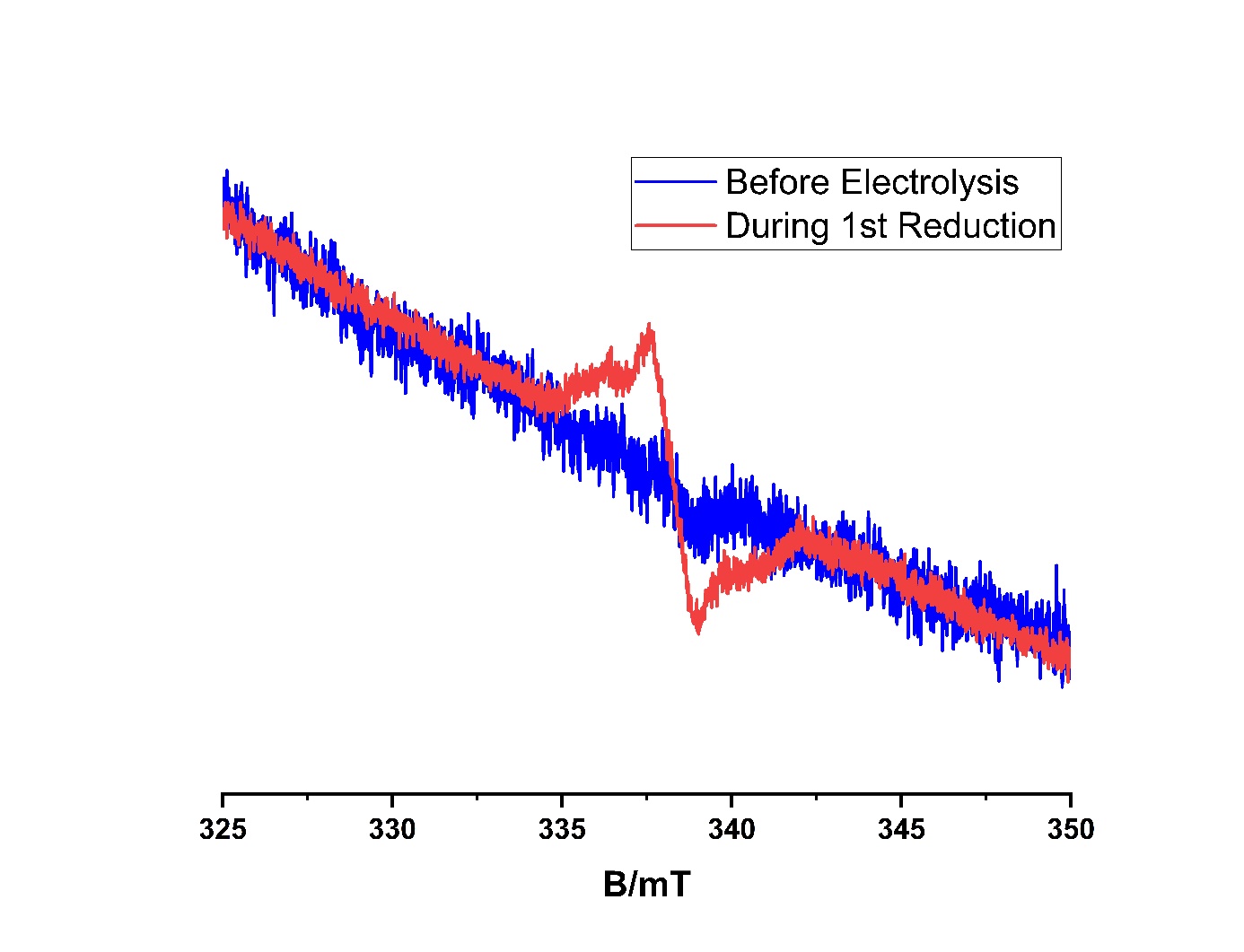


**Figure S37:** Changes in the EPR spectra of **N(I)** in *o*-DFB/0.1M Bu_4_NPF_6_ during the reduction with an Au working electrode.

**7. Computational Details**

Theoretical calculations were performed using ORCA 5.0 and 6.0.^[7]^ Geometry optimisations and frequencies were calculated starting from experimentally determined molecular structures with the PBE0 functional^[8]^ and def2-TZVP^[9]^ basis sets. The optimized geometries were employed for single-point and TD-DFT calculations with the PBE0 functional and def2-TZVP basis sets. Implicit solvation was approximated using the SMD ^[11]^ method together with the CPCM ^[12]^ model. CH_2_Cl_2_ was used as the solvent of choice unless stated otherwise. Population analysis were determined from Löwdin reduced orbital populations. Resolution-of-the-identity (RI) approximations^[13]^ with matching basis-sets (def2/J)^[14]^ were employed to reduce time of calculation. Grimme’s D3 method was used for dispersion correction.^[15]^ The visualization software *Chemcraft* was used to plot orbital and electron density figures.^[16]^ Natural charges and Wiberg bond index were obtained by performing natural bond orbitals (NBO) analysis at the same level using Gaussian 16.^[17]^ Proton affinity, Me Cation affinity, AuCl and BH_3_ complexation energy were calculated following literature reported procedure.^[18]^

Dihedral rotational energy profiles were obtained from appropriate relaxed surface scans using the PBE0 functional with def2-SVP basis sets on all atoms, except for the four atoms involved in the dihedral angle (N3-C7-N1-C8 and C7-N1-C8-C19, numbering starting on 1), for which def2-TZVP basis sets were employed. Full 360º rotations, starting from the initial angles in the optimized structure of **N(I)**, were subdivided in 25 or 15 steps, respectively, as a compromise between detail in the energy surface and calculation time.

Activation energies of the 1,3 H-shift reaction from Zwitterionic intermediate to MII-CDI were obtained in two steps. Firstly, a Nudged Elastic Band (NEB) calculation^[19]^ was performed with pre-optimization of the initial and final structures. The energy profile was used to identify the TS, for which a subsequent single-point plus frequencies calculation was performed, which produced a single imaginary frequency corresponding to the N-H-N asymmetric stretching mode. The initial and final structures were optimized and their frequencies calculated, to verify they were minima.

| 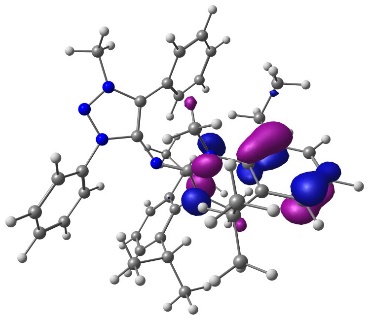 | 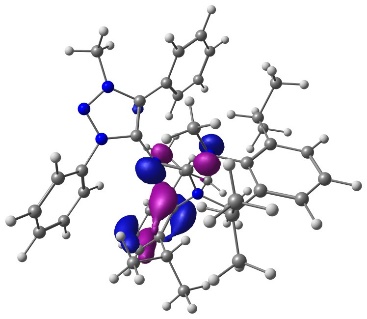 | 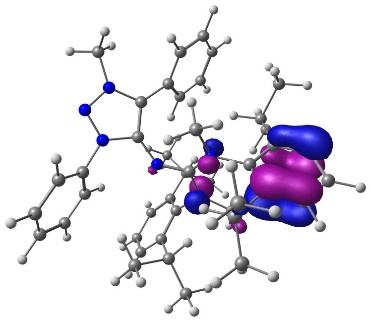 |
| --- | --- | --- |
| HOMO-4 | HOMO-3 | HOMO-2 |
| 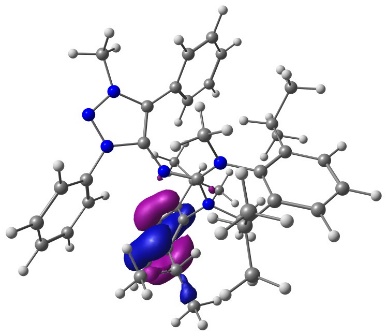 | 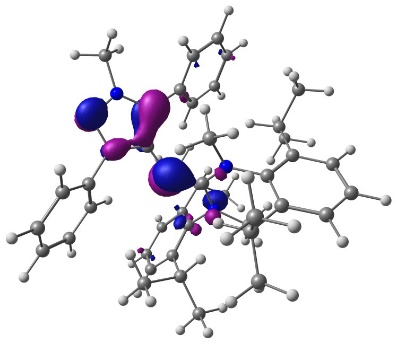 | 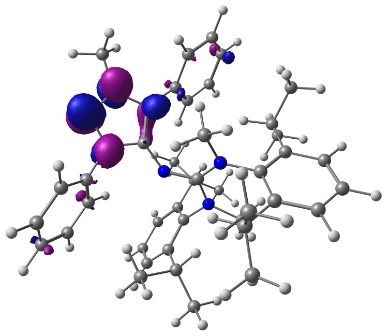 |
| HOMO-1 | HOMO | LUMO |

**Figure S38:** Frontier Molecular Orbitals of Dipp-N(I). Isosurface value = 0.06.

| 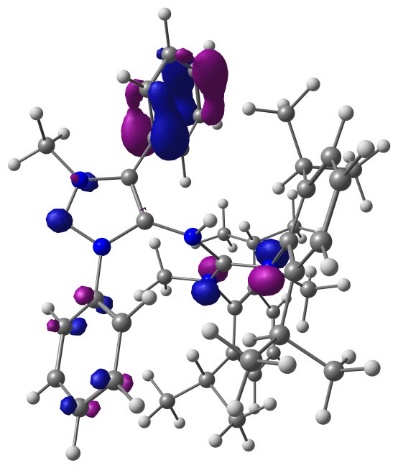 | 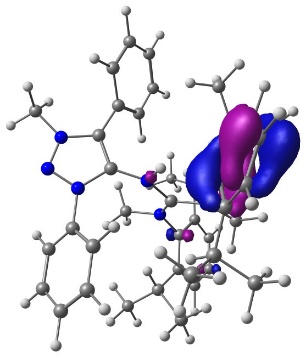 | 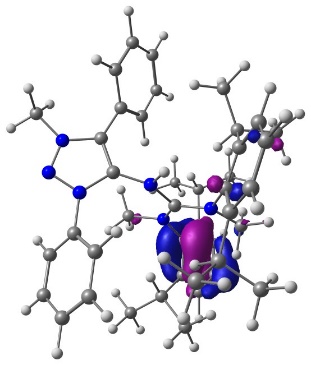 |
| --- | --- | --- |
| HOMO-4 | HOMO-3 | HOMO-2 |
| 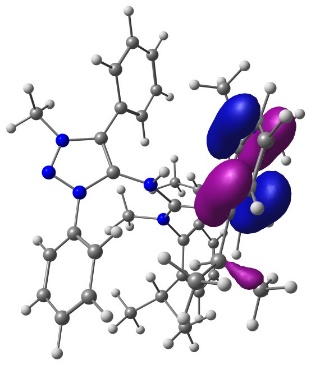 | 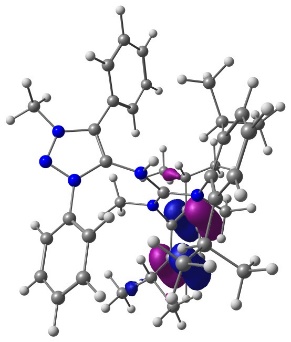 | 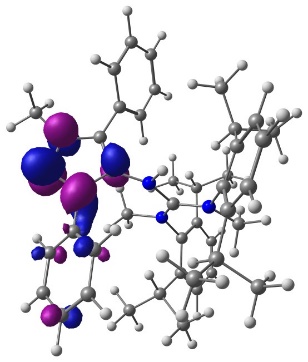 |
| HOMO-1 | HOMO | LUMO |

**Figure S39:** Frontier Molecular Orbitals of **Dipp-N(I)HOTf**. Isosurface value = 0.06.


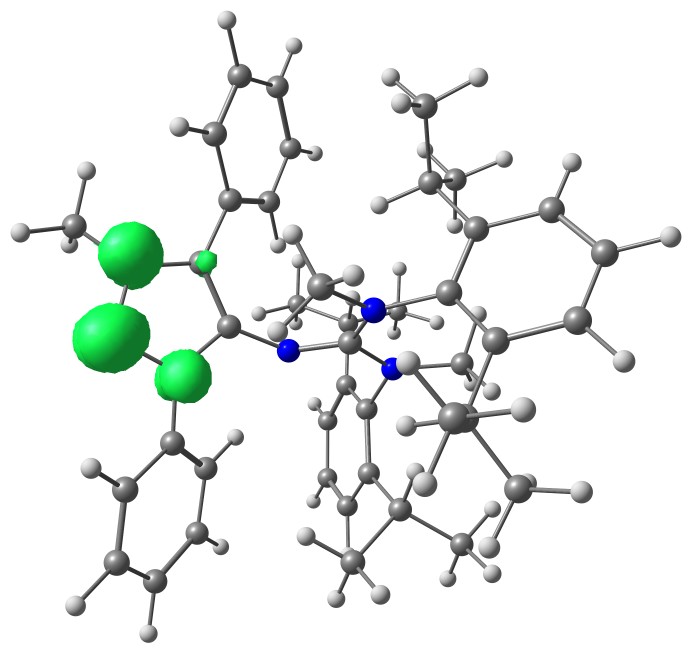


**Figure S40:** Spin Density Plot of the reduced species of **Dipp-N(I)**. Isosurface value = 0.008.

**Table S6:** Optimised molecular structure of **Dipp-N(I)** in XYZ-coordinates.

| **Atom** | **x** | **y** | **z** |
| --- | --- | --- | --- |
| N | 8.94005 | 20.83779 | 3.92365 |
| N | 10.17788 | 19.11908 | 4.66853 |
| N | 7.92586 | 18.71601 | 3.99816 |
| N | 8.06699 | 22.73871 | 2.89082 |
| N | 7.08391 | 23.61439 | 2.96163 |
| N | 6.36405 | 23.21742 | 3.96143 |
| C | 8.96918 | 19.5567 | 4.21748 |
| C | 7.98591 | 21.74476 | 3.84442 |
| C | 11.26964 | 20.05736 | 4.62316 |
| C | 12.06348 | 20.09982 | 3.47186 |
| C | 7.91008 | 17.29687 | 4.22553 |
| C | 11.56056 | 20.83775 | 5.74734 |
| C | 10.39508 | 23.17947 | 2.39771 |
| H | 10.61462 | 23.126 | 3.45642 |
| C | 7.47685 | 16.80996 | 5.46228 |
| C | 8.23849 | 16.43658 | 3.16664 |
| C | 7.46741 | 15.43089 | 5.65505 |
| H | 7.15854 | 15.03119 | 6.61424 |
| C | 6.839 | 22.10219 | 4.57337 |
| C | 9.10571 | 22.95516 | 1.94148 |
| C | 11.38686 | 23.46681 | 1.47492 |
| H | 12.39884 | 23.64218 | 1.81873 |
| C | 6.26716 | 21.57589 | 5.81106 |
| C | 10.75098 | 20.72762 | 7.01701 |
| H | 9.78129 | 20.30061 | 6.7497 |
| C | 10.58249 | 17.75722 | 4.99655 |
| H | 9.95759 | 17.3208 | 5.77072 |
| H | 11.59958 | 17.81986 | 5.3766 |
| H | 10.58663 | 17.09605 | 4.13055 |
| C | 8.19648 | 15.0674 | 3.40222 |
| H | 8.45754 | 14.38205 | 2.60433 |
| C | 7.83282 | 14.56544 | 4.6407 |
| H | 7.81895 | 13.49444 | 4.80886 |
| C | 13.14474 | 20.97518 | 3.45046 |
| H | 13.767 | 21.02784 | 2.56391 |
| C | 11.81132 | 19.19995 | 2.28467 |
| H | 10.91852 | 18.6123 | 2.49601 |
| C | 8.79037 | 22.99322 | 0.59352 |
| H | 7.7731 | 22.80688 | 0.27121 |
| C | 6.9962 | 17.71199 | 6.57466 |
| H | 7.02509 | 18.74243 | 6.21576 |
| C | 12.65209 | 21.69779 | 5.68135 |
| H | 12.89794 | 22.3147 | 6.53748 |
| C | 13.43517 | 21.77453 | 4.54084 |
| H | 14.27976 | 22.45385 | 4.50791 |
| C | 6.74509 | 19.21256 | 3.30388 |
| H | 6.08003 | 19.7634 | 3.96911 |
| H | 6.19859 | 18.35211 | 2.92881 |
| H | 7.02509 | 19.84247 | 2.45851 |
| C | 8.61654 | 16.94104 | 1.79164 |
| H | 8.79438 | 18.01741 | 1.86139 |
| C | 11.08762 | 23.52198 | 0.12036 |
| H | 11.8687 | 23.74649 | -0.5965 |
| C | 7.08458 | 21.47084 | 6.93391 |
| H | 8.12783 | 21.74209 | 6.85427 |
| C | 9.89068 | 16.28844 | 1.26226 |
| H | 9.72766 | 15.23285 | 1.03346 |
| H | 10.71246 | 16.35257 | 1.97691 |
| H | 10.20463 | 16.78182 | 0.33958 |
| C | 4.91639 | 21.24283 | 5.91484 |
| H | 4.27567 | 21.29805 | 5.0423 |
| C | 9.79376 | 23.28028 | -0.32009 |
| H | 9.56133 | 23.31486 | -1.37769 |
| C | 10.511 | 22.07726 | 7.6827 |
| H | 11.43636 | 22.49488 | 8.08582 |
| H | 9.81624 | 21.96449 | 8.51876 |
| H | 10.09365 | 22.80448 | 6.98176 |
| C | 7.88522 | 17.62559 | 7.81181 |
| H | 7.91737 | 16.60566 | 8.2035 |
| H | 7.49489 | 18.27602 | 8.59759 |
| H | 8.9087 | 17.9394 | 7.59726 |
| C | 11.419 | 19.76723 | 8.00096 |
| H | 11.55672 | 18.77541 | 7.56682 |
| H | 10.80977 | 19.66198 | 8.90244 |
| H | 12.4013 | 20.14656 | 8.29595 |
| C | 4.39277 | 20.83794 | 7.13175 |
| H | 3.34221 | 20.58254 | 7.20478 |
| C | 6.55961 | 21.05831 | 8.14713 |
| H | 7.20506 | 20.98847 | 9.01493 |
| C | 5.19723 | 23.99838 | 4.32432 |
| H | 5.29703 | 24.97042 | 3.84929 |
| H | 5.16692 | 24.10417 | 5.40671 |
| H | 4.29455 | 23.49982 | 3.97489 |
| C | 11.54053 | 19.97516 | 1.00129 |
| H | 12.40007 | 20.58561 | 0.71504 |
| H | 10.68049 | 20.63702 | 1.1108 |
| H | 11.33733 | 19.27776 | 0.18437 |
| C | 5.21059 | 20.75004 | 8.25026 |
| H | 4.79676 | 20.43302 | 9.20046 |
| C | 5.54796 | 17.37982 | 6.92766 |
| H | 4.89601 | 17.48547 | 6.05733 |
| H | 5.18745 | 18.05249 | 7.70702 |
| H | 5.45805 | 16.35517 | 7.29658 |
| C | 7.47752 | 16.71605 | 0.79651 |
| H | 7.7501 | 17.1166 | -0.18302 |
| H | 6.55098 | 17.19652 | 1.1141 |
| H | 7.28024 | 15.64688 | 0.68215 |
| C | 12.9742 | 18.22835 | 2.09529 |
| H | 12.77169 | 17.55056 | 1.26282 |
| H | 13.14248 | 17.62912 | 2.99322 |
| H | 13.89813 | 18.76814 | 1.87208 |

**Table S7:** Optimised molecular structure of **Dipp-N(I)HOTf** in XYZ-coordinates.

| **Atom** | **x** | **y** | **z** |
| --- | --- | --- | --- |
| N | 8.68342 | 5.90883 | 11.73617 |
| N | 7.80654 | 3.85114 | 10.99772 |
| H | 6.93909 | 3.32013 | 10.97939 |
| N | 6.45229 | 5.24224 | 12.1701 |
| N | 9.83105 | 2.52114 | 10.75267 |
| N | 10.5791 | 2.02461 | 9.80965 |
| N | 10.06345 | 2.44569 | 8.69314 |
| C | 7.67283 | 5.04679 | 11.6493 |
| C | 8.82236 | 3.29588 | 10.24384 |
| C | 8.65272 | 7.13734 | 12.49514 |
| C | 5.40895 | 4.2598 | 11.96762 |
| C | 8.33407 | 8.3244 | 11.82781 |
| C | 8.97209 | 3.23248 | 8.87444 |
| C | 9.06611 | 7.09974 | 13.82839 |
| C | 10.01211 | 5.64938 | 11.17437 |
| H | 10.50743 | 4.8283 | 11.69163 |
| H | 10.60212 | 6.5446 | 11.34421 |
| H | 9.98062 | 5.47319 | 10.1025 |
| C | 5.15994 | 3.31402 | 12.97486 |
| C | 10.21546 | 2.30816 | 12.11114 |
| C | 9.48683 | 5.82313 | 14.52035 |
| H | 9.27822 | 4.98073 | 13.85621 |
| C | 9.26499 | 1.93544 | 13.04464 |
| H | 8.23672 | 1.78085 | 12.74832 |
| C | 5.97913 | 6.45941 | 12.83876 |
| H | 6.51955 | 6.65452 | 13.7605 |
| H | 4.93337 | 6.28769 | 13.0759 |
| H | 6.04347 | 7.32265 | 12.1831 |
| C | 8.39499 | 9.50395 | 12.56022 |
| H | 8.1445 | 10.44253 | 12.08061 |
| C | 11.55194 | 2.48224 | 12.44178 |
| H | 12.26634 | 2.78074 | 11.68482 |
| C | 8.77008 | 9.49633 | 13.894 |
| H | 8.80812 | 10.42691 | 14.4486 |
| C | 9.10648 | 8.30804 | 14.51816 |
| H | 9.41187 | 8.31634 | 15.55799 |
| C | 8.2492 | 3.86383 | 7.77214 |
| C | 4.61415 | 4.35608 | 10.81475 |
| C | 8.18662 | 5.25335 | 7.69353 |
| H | 8.63618 | 5.86434 | 8.46589 |
| C | 5.92434 | 3.28852 | 14.27814 |
| H | 6.90447 | 3.74483 | 14.11043 |
| C | 11.00274 | 1.91023 | 14.70896 |
| H | 11.31028 | 1.75534 | 15.73616 |
| C | 11.9412 | 2.27622 | 13.75383 |
| H | 12.97948 | 2.41212 | 14.0301 |
| C | 7.95098 | 8.34922 | 10.36517 |
| H | 7.7047 | 7.32793 | 10.06457 |
| C | 9.67215 | 1.73955 | 14.35483 |
| H | 8.94681 | 1.43975 | 15.09933 |
| C | 4.79725 | 5.44489 | 9.78343 |
| H | 5.79534 | 5.87498 | 9.90233 |
| C | 4.11039 | 2.42365 | 12.77338 |
| H | 3.8922 | 1.68008 | 13.52918 |
| C | 3.57698 | 3.44023 | 10.66836 |
| H | 2.94421 | 3.4901 | 9.79113 |
| C | 10.99011 | 5.83823 | 14.79203 |
| H | 11.56374 | 5.95993 | 13.8707 |
| H | 11.29603 | 4.9022 | 15.26362 |
| H | 11.25045 | 6.65985 | 15.46405 |
| C | 8.70993 | 5.58543 | 15.81105 |
| H | 8.94081 | 6.34697 | 16.55936 |
| H | 8.97736 | 4.61286 | 16.23091 |
| H | 7.63251 | 5.59608 | 15.63914 |
| C | 3.33073 | 2.48018 | 11.63204 |
| H | 2.51645 | 1.77706 | 11.49914 |
| C | 7.57381 | 5.85645 | 6.60854 |
| H | 7.53434 | 6.93759 | 6.54868 |
| C | 6.13886 | 1.87846 | 14.81519 |
| H | 5.19913 | 1.43418 | 15.1496 |
| H | 6.80035 | 1.91588 | 15.6826 |
| H | 6.5751 | 1.21066 | 14.07016 |
| C | 10.68899 | 2.06025 | 7.43945 |
| H | 11.71003 | 1.76499 | 7.66422 |
| H | 10.67473 | 2.9164 | 6.76887 |
| H | 10.14153 | 1.22835 | 6.99962 |
| C | 3.77523 | 6.5598 | 10.01256 |
| H | 3.93417 | 7.36616 | 9.29305 |
| H | 3.84322 | 6.98043 | 11.01672 |
| H | 2.76122 | 6.17524 | 9.87532 |
| C | 7.6799 | 3.08209 | 6.76838 |
| H | 7.71293 | 2.00082 | 6.83643 |
| C | 6.72422 | 9.21104 | 10.08585 |
| H | 5.86989 | 8.919 | 10.69977 |
| H | 6.43584 | 9.10818 | 9.03731 |
| H | 6.92749 | 10.26812 | 10.26996 |
| C | 4.68756 | 4.92273 | 8.35769 |
| H | 3.68042 | 4.56147 | 8.13998 |
| H | 5.39169 | 4.11043 | 8.17153 |
| H | 4.90529 | 5.72756 | 7.65489 |
| C | 9.12896 | 8.81386 | 9.50902 |
| H | 9.40057 | 9.84198 | 9.76175 |
| H | 8.85916 | 8.78399 | 8.4504 |
| H | 10.01102 | 8.18754 | 9.65408 |
| C | 5.1854 | 4.1136 | 15.33502 |
| H | 5.0503 | 5.15252 | 15.03523 |
| H | 5.74104 | 4.10033 | 16.27535 |
| H | 4.19726 | 3.68378 | 15.51842 |
| C | 7.01542 | 5.07768 | 5.60503 |
| H | 6.5363 | 5.55119 | 4.7562 |
| C | 7.06183 | 3.69283 | 5.68977 |
| H | 6.61568 | 3.08403 | 4.91269 |

**Table S8:** Optimised molecular structure of **Dipp-N(I)AuCl** in XYZ-coordinates.

| **Atom** | **x** | **y** | **z** |
| --- | --- | --- | --- |
| N | 8.65272 | 5.86267 | 11.67515 |
| N | 7.85392 | 3.76283 | 10.93955 |
| N | 6.44745 | 5.14807 | 12.11315 |
| N | 10.01072 | 2.63081 | 10.74966 |
| N | 10.83475 | 2.22161 | 9.81207 |
| N | 10.32459 | 2.65127 | 8.69879 |
| C | 7.67049 | 4.93269 | 11.57593 |
| C | 8.95493 | 3.34165 | 10.23611 |
| C | 8.61217 | 7.07551 | 12.45781 |
| C | 5.33158 | 4.24398 | 11.96245 |
| C | 8.28415 | 8.27694 | 11.81844 |
| C | 9.17308 | 3.34248 | 8.86714 |
| C | 9.05137 | 7.02884 | 13.7835 |
| C | 10.00307 | 5.66685 | 11.14352 |
| H | 10.5385 | 4.8805 | 11.67484 |
| H | 10.54283 | 6.59345 | 11.31392 |
| H | 10.0107 | 5.48081 | 10.07337 |
| C | 4.99398 | 3.40865 | 13.03971 |
| C | 10.36342 | 2.37639 | 12.10718 |
| C | 9.48162 | 5.74579 | 14.45637 |
| H | 9.26865 | 4.9132 | 13.78219 |
| C | 9.39432 | 2.00392 | 13.02339 |
| H | 8.36288 | 1.89442 | 12.71747 |
| C | 6.00332 | 6.37194 | 12.79236 |
| H | 6.5598 | 6.56533 | 13.70448 |
| H | 4.96166 | 6.21377 | 13.05597 |
| H | 6.05486 | 7.2375 | 12.13836 |
| C | 8.36207 | 9.44765 | 12.56305 |
| H | 8.10312 | 10.39208 | 12.09956 |
| C | 11.69843 | 2.50496 | 12.46738 |
| H | 12.43218 | 2.80723 | 11.73131 |
| C | 8.76283 | 9.42511 | 13.88923 |
| H | 8.81388 | 10.34877 | 14.45449 |
| C | 9.10751 | 8.2274 | 14.48941 |
| H | 9.43282 | 8.21978 | 15.52342 |
| C | 8.40552 | 3.92359 | 7.76823 |
| C | 4.47831 | 4.40477 | 10.85844 |
| C | 8.20471 | 5.30061 | 7.70763 |
| H | 8.59533 | 5.94131 | 8.48762 |
| C | 5.86854 | 3.26249 | 14.26222 |
| H | 6.8392 | 3.71406 | 14.04046 |
| C | 11.10732 | 1.87467 | 14.70968 |
| H | 11.39732 | 1.67982 | 15.73528 |
| C | 12.06567 | 2.24576 | 13.77642 |
| H | 13.10391 | 2.34593 | 14.06845 |
| C | 7.86239 | 8.32278 | 10.36709 |
| H | 7.57261 | 7.3106 | 10.07438 |
| C | 9.77801 | 1.75585 | 14.33197 |
| H | 9.03088 | 1.45812 | 15.05617 |
| C | 4.79467 | 5.3351 | 9.71178 |
| H | 5.8329 | 5.66105 | 9.80928 |
| C | 3.78545 | 2.72291 | 12.97992 |
| H | 3.50383 | 2.07297 | 13.79974 |
| C | 3.28262 | 3.69441 | 10.84942 |
| H | 2.60823 | 3.80308 | 10.00846 |
| C | 10.98605 | 5.76149 | 14.72064 |
| H | 11.55501 | 5.89952 | 13.79882 |
| H | 11.29796 | 4.8193 | 15.17558 |
| H | 11.24687 | 6.57363 | 15.4042 |
| C | 8.71513 | 5.49051 | 15.74986 |
| H | 8.95421 | 6.24083 | 16.50721 |
| H | 8.9828 | 4.51102 | 16.15343 |
| H | 7.63674 | 5.50632 | 15.58691 |
| C | 2.93379 | 2.86435 | 11.89938 |
| H | 1.99327 | 2.32605 | 11.87581 |
| C | 7.52982 | 5.85668 | 6.63384 |
| H | 7.38374 | 6.92959 | 6.59016 |
| C | 6.10037 | 1.79881 | 14.62252 |
| H | 5.17296 | 1.31618 | 14.93834 |
| H | 6.80375 | 1.72777 | 15.45485 |
| H | 6.49739 | 1.2333 | 13.77678 |
| C | 11.01133 | 2.3611 | 7.45345 |
| H | 12.04119 | 2.11654 | 7.69792 |
| H | 10.96566 | 3.24327 | 6.81866 |
| H | 10.52959 | 1.51937 | 6.95855 |
| C | 3.90582 | 6.57656 | 9.76101 |
| H | 4.17359 | 7.26409 | 8.95511 |
| H | 4.0048 | 7.10773 | 10.70941 |
| H | 2.85559 | 6.2997 | 9.63655 |
| C | 7.90288 | 3.10519 | 6.75797 |
| H | 8.03601 | 2.03099 | 6.81619 |
| C | 6.66073 | 9.23008 | 10.12581 |
| H | 5.82068 | 8.97648 | 10.77545 |
| H | 6.32795 | 9.13026 | 9.09022 |
| H | 6.9121 | 10.28006 | 10.2914 |
| C | 4.65855 | 4.63358 | 8.36597 |
| H | 3.63064 | 4.31119 | 8.18628 |
| H | 5.30607 | 3.75588 | 8.3112 |
| H | 4.93947 | 5.31374 | 7.56124 |
| C | 9.02911 | 8.75121 | 9.47723 |
| H | 9.34738 | 9.7658 | 9.73095 |
| H | 8.72519 | 8.74331 | 8.42729 |
| H | 9.89119 | 8.09092 | 9.58653 |
| C | 5.25672 | 4.00084 | 15.45243 |
| H | 5.1183 | 5.06359 | 15.24584 |
| H | 5.90034 | 3.90465 | 16.33001 |
| H | 4.28059 | 3.57582 | 15.70052 |
| C | 7.04511 | 5.04138 | 5.62081 |
| H | 6.51764 | 5.47743 | 4.78047 |
| C | 7.22434 | 3.66642 | 5.68846 |
| H | 6.83284 | 3.02674 | 4.90655 |
| Au | 6.56889 | 2.12357 | 10.65618 |
| Cl | 5.44101 | 0.20799 | 10.21737 |

**Table S9:** Optimised molecular structure of **Dipp-N(I)BH_3_** in XYZ-coordinates.

| **Atom** | **x** | **y** | **z** |
| --- | --- | --- | --- |
| N | 8.62047 | 5.81278 | 11.67968 |
| N | 7.85114 | 3.67645 | 10.99246 |
| N | 6.4119 | 5.12763 | 12.10592 |
| N | 10.04874 | 2.67137 | 10.69318 |
| N | 10.86616 | 2.33621 | 9.7256 |
| N | 10.32064 | 2.7962 | 8.6418 |
| C | 7.63469 | 4.87462 | 11.59218 |
| C | 8.96013 | 3.36441 | 10.23065 |
| C | 8.58751 | 7.03023 | 12.46341 |
| C | 5.25875 | 4.25889 | 12.00569 |
| C | 8.29097 | 8.23875 | 11.82202 |
| C | 9.14415 | 3.43552 | 8.8631 |
| C | 9.0221 | 6.9784 | 13.79103 |
| C | 9.99814 | 5.6146 | 11.2138 |
| H | 10.50347 | 4.82237 | 11.76339 |
| H | 10.53016 | 6.53714 | 11.42482 |
| H | 10.06551 | 5.44618 | 10.14316 |
| C | 4.87585 | 3.54648 | 13.14781 |
| C | 10.42691 | 2.36654 | 12.03442 |
| C | 9.41614 | 5.689 | 14.4742 |
| H | 9.15875 | 4.85565 | 13.81647 |
| C | 9.46983 | 2.02049 | 12.97618 |
| H | 8.42605 | 1.94966 | 12.70412 |
| C | 6.00232 | 6.37319 | 12.77233 |
| H | 6.53407 | 6.54422 | 13.70354 |
| H | 4.94645 | 6.26013 | 12.99732 |
| H | 6.11168 | 7.23332 | 12.12067 |
| C | 8.40105 | 9.4098 | 12.56171 |
| H | 8.16588 | 10.35847 | 12.09445 |
| C | 11.77627 | 2.43616 | 12.35899 |
| H | 12.50081 | 2.72464 | 11.60862 |
| C | 8.802 | 9.38279 | 13.88759 |
| H | 8.87923 | 10.30701 | 14.4489 |
| C | 9.11062 | 8.17794 | 14.49218 |
| H | 9.43349 | 8.16456 | 15.52683 |
| C | 8.35296 | 4.0397 | 7.79129 |
| C | 4.45655 | 4.32203 | 10.86505 |
| C | 8.13124 | 5.41406 | 7.77465 |
| H | 8.51604 | 6.03406 | 8.57313 |
| C | 5.71652 | 3.51025 | 14.40338 |
| H | 6.67643 | 3.98077 | 14.17797 |
| C | 11.2265 | 1.79742 | 14.61011 |
| H | 11.53914 | 1.577 | 15.62389 |
| C | 12.17107 | 2.14156 | 13.65218 |
| H | 13.22089 | 2.19552 | 13.91351 |
| C | 7.85939 | 8.29103 | 10.37397 |
| H | 7.50793 | 7.29314 | 10.09905 |
| C | 9.88329 | 1.7413 | 14.27017 |
| H | 9.1426 | 1.47019 | 15.01257 |
| C | 4.82725 | 5.14832 | 9.65616 |
| H | 5.90153 | 5.34239 | 9.69287 |
| C | 3.67069 | 2.85785 | 13.11272 |
| H | 3.35746 | 2.28971 | 13.98122 |
| C | 3.25744 | 3.61496 | 10.87723 |
| H | 2.61741 | 3.639 | 10.00377 |
| C | 10.92464 | 5.64889 | 14.71414 |
| H | 11.48659 | 5.76291 | 13.78527 |
| H | 11.20782 | 4.69703 | 15.16805 |
| H | 11.22453 | 6.45302 | 15.39119 |
| C | 8.6693 | 5.48175 | 15.78807 |
| H | 8.97395 | 6.21673 | 16.53678 |
| H | 8.8892 | 4.48871 | 16.18745 |
| H | 7.58971 | 5.56306 | 15.65606 |
| C | 2.86605 | 2.8868 | 11.98598 |
| H | 1.92652 | 2.34557 | 11.97523 |
| C | 7.44729 | 5.99589 | 6.71987 |
| H | 7.28397 | 7.06721 | 6.71346 |
| C | 6.00293 | 2.08562 | 14.86949 |
| H | 5.08806 | 1.58244 | 15.1915 |
| H | 6.68664 | 2.10582 | 15.72195 |
| H | 6.45674 | 1.48933 | 14.07662 |
| C | 10.99734 | 2.59522 | 7.37389 |
| H | 12.03055 | 2.34107 | 7.59304 |
| H | 10.94157 | 3.51784 | 6.80013 |
| H | 10.51645 | 1.78622 | 6.82663 |
| C | 4.1062 | 6.49535 | 9.68127 |
| H | 4.4211 | 7.10829 | 8.83276 |
| H | 4.31079 | 7.05239 | 10.59657 |
| H | 3.02499 | 6.3473 | 9.61166 |
| C | 7.86895 | 3.24938 | 6.74999 |
| H | 8.02181 | 2.17648 | 6.77037 |
| C | 6.70853 | 9.26135 | 10.1308 |
| H | 5.86619 | 9.07062 | 10.79899 |
| H | 6.35401 | 9.15891 | 9.10277 |
| H | 7.02276 | 10.29825 | 10.26903 |
| C | 4.54042 | 4.42872 | 8.34461 |
| H | 3.4685 | 4.284 | 8.19223 |
| H | 5.02885 | 3.45371 | 8.31318 |
| H | 4.91277 | 5.02394 | 7.51009 |
| C | 9.04034 | 8.64432 | 9.46994 |
| H | 9.41614 | 9.64183 | 9.71224 |
| H | 8.72854 | 8.64472 | 8.42228 |
| H | 9.86626 | 7.93929 | 9.57963 |
| C | 5.04584 | 4.2989 | 15.52753 |
| H | 4.86958 | 5.33929 | 15.2483 |
| H | 5.66963 | 4.28682 | 16.4247 |
| H | 4.08181 | 3.85103 | 15.78264 |
| C | 6.98207 | 5.20876 | 5.67611 |
| H | 6.45087 | 5.66454 | 4.84868 |
| C | 7.18737 | 3.83574 | 5.69629 |
| H | 6.81242 | 3.21706 | 4.88973 |
| H | 6.48888 | 2.0162 | 11.95703 |
| H | 6.11212 | 2.44094 | 10.01767 |
| H | 7.74802 | 1.45622 | 10.55133 |
| B | 6.94364 | 2.30701 | 10.88333 |

**Table S10:** Optimised molecular structure of **Dipp-N(I)Me^+^** in XYZ-coordinates.

| **Atom** | **x** | **y** | **z** |
| --- | --- | --- | --- |
| N | 8.6307 | 5.77408 | 11.73209 |
| N | 7.87463 | 3.65654 | 11.02193 |
| N | 6.40225 | 5.13508 | 12.11224 |
| N | 10.0466 | 2.61689 | 10.65905 |
| N | 10.8342 | 2.28364 | 9.67475 |
| N | 10.28189 | 2.76464 | 8.60322 |
| C | 7.62894 | 4.87724 | 11.63073 |
| C | 8.97112 | 3.34944 | 10.21791 |
| C | 8.59058 | 7.0182 | 12.4803 |
| C | 5.23443 | 4.28014 | 12.00917 |
| C | 8.32075 | 8.2081 | 11.79816 |
| C | 9.11823 | 3.42149 | 8.84966 |
| C | 8.99423 | 6.98963 | 13.81816 |
| C | 10.01835 | 5.54298 | 11.30639 |
| H | 10.47278 | 4.72068 | 11.85391 |
| H | 10.56979 | 6.44097 | 11.56555 |
| H | 10.11459 | 5.40886 | 10.23321 |
| C | 4.86667 | 3.53617 | 13.1374 |
| C | 10.47151 | 2.32243 | 11.99306 |
| C | 9.36181 | 5.71344 | 14.54129 |
| H | 9.1105 | 4.86413 | 13.9009 |
| C | 9.56168 | 1.91557 | 12.95519 |
| H | 8.5211 | 1.76247 | 12.71863 |
| C | 5.99527 | 6.38665 | 12.78178 |
| H | 6.52756 | 6.54827 | 13.71316 |
| H | 4.93846 | 6.2754 | 13.00208 |
| H | 6.11434 | 7.24279 | 12.12714 |
| C | 8.41834 | 9.39565 | 12.51361 |
| H | 8.20276 | 10.33419 | 12.01748 |
| C | 11.82084 | 2.47773 | 12.28517 |
| H | 12.50809 | 2.81162 | 11.51857 |
| C | 8.78475 | 9.39685 | 13.84916 |
| H | 8.85307 | 10.3335 | 14.39037 |
| C | 9.07248 | 8.20551 | 14.49081 |
| H | 9.36958 | 8.21649 | 15.53298 |
| C | 8.32341 | 4.03413 | 7.785 |
| C | 4.43266 | 4.36212 | 10.8689 |
| C | 8.16238 | 5.4158 | 7.73787 |
| H | 8.58863 | 6.03798 | 8.51333 |
| C | 5.6987 | 3.49645 | 14.39967 |
| H | 6.65011 | 3.99411 | 14.19741 |
| C | 11.36033 | 1.8183 | 14.5504 |
| H | 11.70795 | 1.62498 | 15.55809 |
| C | 12.26036 | 2.21576 | 13.57079 |
| H | 13.30985 | 2.33739 | 13.80885 |
| C | 7.94062 | 8.22965 | 10.33582 |
| H | 7.62734 | 7.21978 | 10.06035 |
| C | 10.01665 | 1.67019 | 14.24107 |
| H | 9.31104 | 1.35218 | 14.99864 |
| C | 4.77911 | 5.23228 | 9.6835 |
| H | 5.83456 | 5.50792 | 9.75037 |
| C | 3.67951 | 2.81698 | 13.07661 |
| H | 3.37559 | 2.22178 | 13.92974 |
| C | 3.25207 | 3.62373 | 10.86055 |
| H | 2.61085 | 3.65883 | 9.98845 |
| C | 10.86486 | 5.66429 | 14.81308 |
| H | 11.44799 | 5.74329 | 13.89376 |
| H | 11.1268 | 4.72379 | 15.30227 |
| H | 11.15989 | 6.48485 | 15.47194 |
| C | 8.58667 | 5.54712 | 15.84491 |
| H | 8.88473 | 6.29765 | 16.58037 |
| H | 8.78927 | 4.56275 | 16.27346 |
| H | 7.51032 | 5.63585 | 15.69165 |
| C | 2.87934 | 2.85419 | 11.94742 |
| H | 1.95538 | 2.28819 | 11.91876 |
| C | 7.4879 | 5.99732 | 6.67711 |
| H | 7.37132 | 7.07405 | 6.64256 |
| C | 6.01303 | 2.07078 | 14.84601 |
| H | 5.10792 | 1.54352 | 15.15538 |
| H | 6.69225 | 2.09421 | 15.70125 |
| H | 6.48615 | 1.485 | 14.05515 |
| C | 10.93404 | 2.56363 | 7.32025 |
| H | 11.96906 | 2.30177 | 7.52093 |
| H | 10.87252 | 3.48996 | 6.75347 |
| H | 10.43589 | 1.75955 | 6.78147 |
| C | 3.95824 | 6.52112 | 9.71462 |
| H | 4.24404 | 7.16964 | 8.88318 |
| H | 4.10378 | 7.07385 | 10.64417 |
| H | 2.89317 | 6.29388 | 9.61959 |
| C | 7.7959 | 3.2354 | 6.77149 |
| H | 7.91175 | 2.15841 | 6.814 |
| C | 6.77155 | 9.16229 | 10.03802 |
| H | 5.90693 | 8.94696 | 10.66902 |
| H | 6.46612 | 9.04519 | 8.9959 |
| H | 7.04686 | 10.20876 | 10.18528 |
| C | 4.57869 | 4.51149 | 8.35644 |
| H | 3.52669 | 4.27611 | 8.18308 |
| H | 5.1486 | 3.58147 | 8.30934 |
| H | 4.91192 | 5.15002 | 7.53811 |
| C | 9.14686 | 8.60409 | 9.47494 |
| H | 9.48578 | 9.61425 | 9.71822 |
| H | 8.87767 | 8.58344 | 8.41581 |
| H | 9.98609 | 7.92285 | 9.62857 |
| C | 4.99602 | 4.25222 | 15.52683 |
| H | 4.79886 | 5.29172 | 15.25885 |
| H | 5.6118 | 4.24213 | 16.42898 |
| H | 4.04075 | 3.77754 | 15.76461 |
| C | 6.97413 | 5.2027 | 5.66219 |
| H | 6.45103 | 5.65907 | 4.83005 |
| C | 7.12224 | 3.82305 | 5.714 |
| H | 6.71176 | 3.201 | 4.92781 |
| C | 6.95591 | 2.49221 | 10.98563 |
| H | 6.53334 | 2.28733 | 11.96 |
| H | 6.1675 | 2.62903 | 10.2511 |
| H | 7.5553 | 1.63441 | 10.68801 |

**Table S11:** Optimised molecular structure of **Dipp-N(I) reduced** in XYZ-coordinates.

| **Atom** | **x** | **y** | **z** |
| --- | --- | --- | --- |
| N | 8.91699 | 20.90826 | 4.17533 |
| N | 10.21084 | 19.14137 | 4.68469 |
| N | 7.96873 | 18.74214 | 3.97698 |
| N | 7.86841 | 22.51731 | 2.77296 |
| N | 6.74327 | 23.29269 | 2.68562 |
| N | 6.00179 | 22.93176 | 3.78438 |
| C | 8.9799 | 19.6124 | 4.28469 |
| C | 7.8843 | 21.74146 | 3.93011 |
| C | 11.31061 | 20.06267 | 4.62807 |
| C | 12.1207 | 20.06851 | 3.48637 |
| C | 7.95636 | 17.32358 | 4.18909 |
| C | 11.60803 | 20.86528 | 5.73618 |
| C | 10.23758 | 22.83738 | 2.31916 |
| H | 10.48031 | 22.56637 | 3.33688 |
| C | 7.52186 | 16.81993 | 5.42038 |
| C | 8.27018 | 16.46893 | 3.12075 |
| C | 7.48588 | 15.43915 | 5.59406 |
| H | 7.16902 | 15.03243 | 6.54797 |
| C | 6.73587 | 22.06358 | 4.60693 |
| C | 8.9107 | 22.79718 | 1.88772 |
| C | 11.23312 | 23.21703 | 1.43481 |
| H | 12.25954 | 23.24151 | 1.7823 |
| C | 6.2141 | 21.57416 | 5.86684 |
| C | 10.7903 | 20.78766 | 7.00176 |
| H | 9.84171 | 20.31432 | 6.74117 |
| C | 10.59533 | 17.78203 | 5.03068 |
| H | 9.95572 | 17.35823 | 5.80035 |
| H | 11.60625 | 17.83709 | 5.43049 |
| H | 10.61022 | 17.10389 | 4.17608 |
| C | 8.20498 | 15.09681 | 3.33592 |
| H | 8.45393 | 14.41926 | 2.52733 |
| C | 7.83221 | 14.58149 | 4.56611 |
| H | 7.79746 | 13.50829 | 4.71739 |
| C | 13.21248 | 20.93205 | 3.45353 |
| H | 13.84634 | 20.9524 | 2.57368 |
| C | 11.89 | 19.1205 | 2.33223 |
| H | 10.97166 | 18.57172 | 2.53788 |
| C | 8.60127 | 23.14072 | 0.56975 |
| H | 7.56885 | 23.11708 | 0.24526 |
| C | 7.05513 | 17.71562 | 6.54252 |
| H | 7.16234 | 18.75189 | 6.21943 |
| C | 12.70727 | 21.71321 | 5.66085 |
| H | 12.95161 | 22.34684 | 6.50558 |
| C | 13.50027 | 21.75732 | 4.52462 |
| H | 14.35114 | 22.42841 | 4.48196 |
| C | 6.75503 | 19.2286 | 3.33408 |
| H | 6.06164 | 19.6784 | 4.04616 |
| H | 6.26457 | 18.37217 | 2.87878 |
| H | 6.98584 | 19.94774 | 2.54824 |
| C | 8.66334 | 16.98556 | 1.75526 |
| H | 8.85433 | 18.05796 | 1.84602 |
| C | 10.93172 | 23.55932 | 0.12322 |
| H | 11.71794 | 23.85716 | -0.5606 |
| C | 7.07203 | 21.35767 | 6.94966 |
| H | 8.1303 | 21.5292 | 6.81579 |
| C | 9.9329 | 16.31888 | 1.2325 |
| H | 9.75324 | 15.27068 | 0.98202 |
| H | 10.74182 | 16.35418 | 1.96352 |
| H | 10.27239 | 16.82198 | 0.32422 |
| C | 4.84267 | 21.35943 | 6.05943 |
| H | 4.15978 | 21.48895 | 5.22756 |
| C | 9.60936 | 23.51575 | -0.30237 |
| H | 9.35717 | 23.77716 | -1.32412 |
| C | 10.49358 | 22.15979 | 7.59417 |
| H | 11.40256 | 22.64299 | 7.96029 |
| H | 9.81175 | 22.06413 | 8.44299 |
| H | 10.03346 | 22.82202 | 6.85679 |
| C | 7.87853 | 17.53964 | 7.81457 |
| H | 7.82505 | 16.51216 | 8.18365 |
| H | 7.49609 | 18.1981 | 8.59753 |
| H | 8.92986 | 17.78838 | 7.6543 |
| C | 11.4803 | 19.89755 | 8.03472 |
| H | 11.66183 | 18.89533 | 7.64111 |
| H | 10.86188 | 19.80428 | 8.93151 |
| H | 12.44335 | 20.32488 | 8.32834 |
| C | 4.35257 | 20.97621 | 7.29756 |
| H | 3.28719 | 20.81819 | 7.42427 |
| C | 6.58074 | 20.96798 | 8.18252 |
| H | 7.26801 | 20.81995 | 9.00827 |
| C | 5.23956 | 24.0419 | 4.31769 |
| H | 5.89734 | 24.79556 | 4.76762 |
| H | 4.53673 | 23.68543 | 5.06696 |
| H | 4.68332 | 24.49787 | 3.49919 |
| C | 11.69932 | 19.82549 | 0.99544 |
| H | 12.57658 | 20.41911 | 0.72621 |
| H | 10.83886 | 20.49458 | 1.01855 |
| H | 11.54238 | 19.08436 | 0.20734 |
| C | 5.21526 | 20.7856 | 8.36886 |
| H | 4.8285 | 20.49091 | 9.33754 |
| C | 5.57404 | 17.46764 | 6.82163 |
| H | 4.9731 | 17.62482 | 5.9228 |
| H | 5.21776 | 18.1522 | 7.59297 |
| H | 5.40721 | 16.44365 | 7.16654 |
| C | 7.53265 | 16.78969 | 0.74498 |
| H | 7.82269 | 17.19623 | -0.22719 |
| H | 6.6104 | 17.28231 | 1.05633 |
| H | 7.31911 | 15.72504 | 0.61628 |
| C | 13.03453 | 18.11179 | 2.24948 |
| H | 12.8532 | 17.39229 | 1.44751 |
| H | 13.1487 | 17.56111 | 3.18614 |
| H | 13.98043 | 18.61819 | 2.03929 |


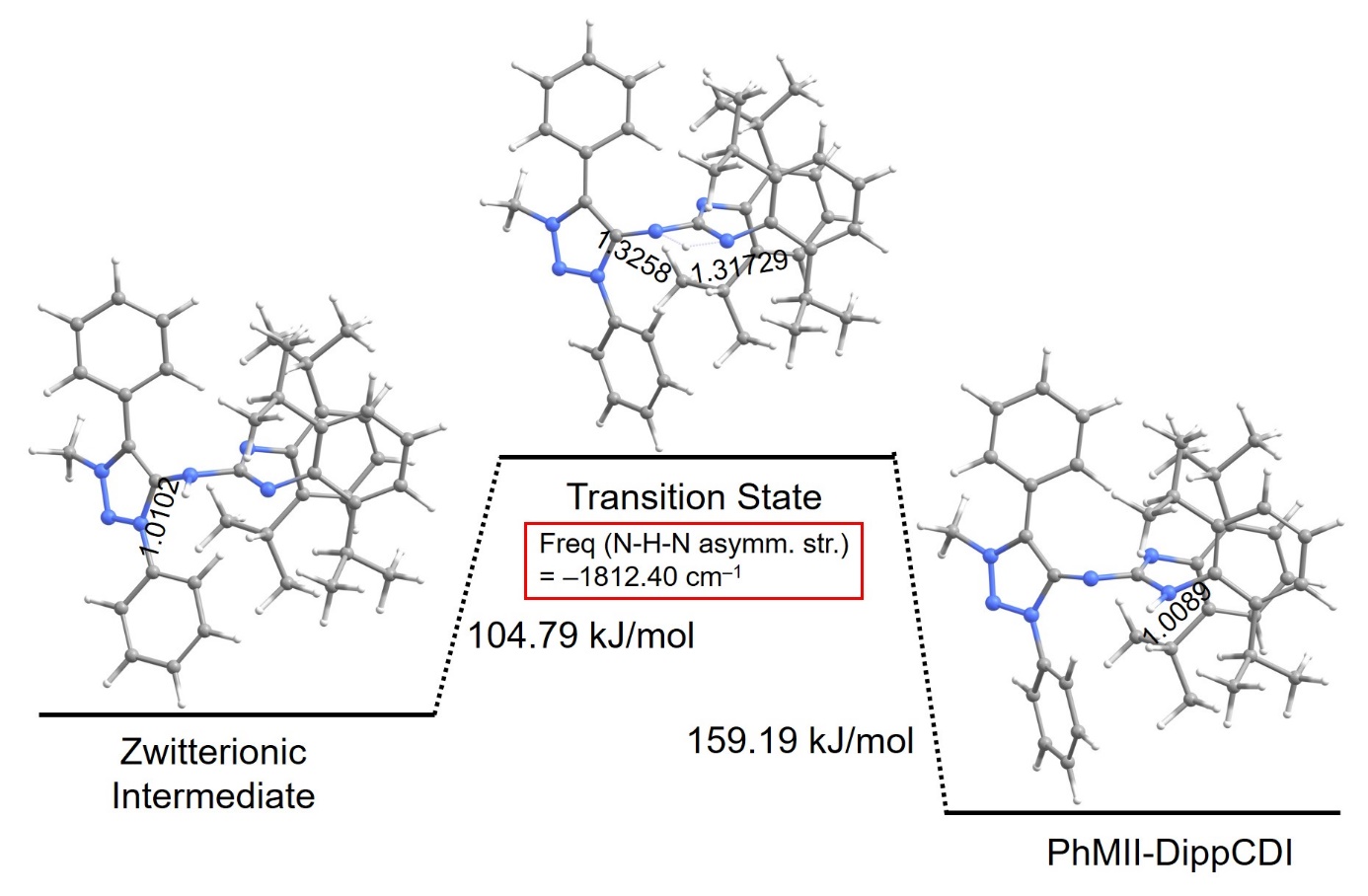


**Figure S41.** Energy profile for the 1,3 H-shift reaction. The activation Gibbs free energy for the reaction is 104.79 kJ/mol, and the reaction is exergonic by 54.4 kJ/mol. The TS has a unique imaginary frequency (–1812.40 cm^–1^) associated with an N-H-N assymetric stretching mode. The corresponding N-H bond distances in Å are given for each structure.

**Table S12:** Coordinates from optimised molecular structures of the Reactant, Transition State and Product in the scheme shown in Figure S24.

| **Atom** | **x** | **y** | **z** |
| --- | --- | --- | --- |
| **Zwitterionic Intermediate** | | | |
| N | 0.406599 | 0.430806 | -0.477088 |
| N | -0.517190 | -1.623364 | -0.977671 |
| H | -1.175885 | -2.316129 | -0.651148 |
| N | -1.356612 | -0.564614 | 0.840805 |
| N | 1.510519 | -2.982556 | -1.042792 |
| N | 2.521501 | -3.194469 | -1.844396 |
| N | 2.293859 | -2.466008 | -2.897951 |
| C | -0.478217 | -0.468094 | -0.128390 |
| C | 0.619589 | -2.085548 | -1.577200 |
| C | 0.831119 | 1.418389 | 0.407382 |
| C | -1.928132 | 0.561252 | 1.414089 |
| C | 0.495196 | 2.772768 | 0.198232 |
| C | 1.129801 | -1.778247 | -2.824502 |
| C | 1.739353 | 1.092934 | 1.443343 |
| C | -2.846341 | 1.333980 | 0.661881 |
| C | 1.446589 | -3.740905 | 0.169470 |
| C | 2.299140 | -0.307001 | 1.535852 |
| H | 1.479165 | -0.992911 | 1.325137 |
| C | 0.633077 | -3.353547 | 1.226755 |
| H | 0.008777 | -2.470464 | 1.189584 |
| C | 0.953642 | 3.736568 | 1.089058 |
| H | 0.669887 | 4.772567 | 0.943797 |
| C | 2.233700 | -4.884033 | 0.254300 |
| H | 2.846349 | -5.184307 | -0.584203 |
| C | 1.768333 | 3.403257 | 2.158459 |
| H | 2.104051 | 4.165854 | 2.852695 |
| C | 2.175913 | 2.087560 | 2.308937 |
| H | 2.850934 | 1.830654 | 3.117190 |
| C | 0.529481 | -0.999722 | -3.900999 |
| C | -1.724217 | 0.860142 | 2.776880 |
| C | -0.160806 | 0.174113 | -3.602820 |
| H | -0.162878 | 0.531524 | -2.577665 |
| C | -3.223336 | 0.898609 | -0.738993 |
| H | -2.308600 | 0.588190 | -1.251268 |
| C | 1.421569 | -5.253881 | 2.486712 |
| H | 1.412456 | -5.842990 | 3.396040 |
| C | 2.221277 | -5.632747 | 1.417188 |
| H | 2.836989 | -6.521805 | 1.481144 |
| C | -0.277909 | 3.172292 | -1.034067 |
| H | -0.951506 | 2.345322 | -1.270333 |
| C | 0.630822 | -4.121271 | 2.382123 |
| H | 0.001126 | -3.815199 | 3.208507 |
| C | -0.856327 | -0.052564 | 3.607832 |
| H | -0.045768 | -0.385550 | 2.954514 |
| C | -3.455279 | 2.430291 | 1.258863 |
| H | -4.144589 | 3.037007 | 0.682830 |
| C | -2.356223 | 1.966093 | 3.332683 |
| H | -2.179912 | 2.214525 | 4.373076 |
| C | 3.362597 | -0.513945 | 0.457007 |
| H | 2.960782 | -0.292869 | -0.533420 |
| H | 3.727101 | -1.545542 | 0.464054 |
| H | 4.216391 | 0.145518 | 0.638082 |
| C | 2.862683 | -0.685089 | 2.896411 |
| H | 3.768558 | -0.119418 | 3.131387 |
| H | 3.129834 | -1.745220 | 2.899967 |
| H | 2.141631 | -0.514978 | 3.698113 |
| C | -3.204004 | 2.764494 | 2.581105 |
| H | -3.679514 | 3.631311 | 3.027022 |
| C | -0.799380 | 0.873843 | -4.613930 |
| H | -1.331310 | 1.787602 | -4.375345 |
| C | -3.867808 | 1.983975 | -1.587984 |
| H | -4.849895 | 2.270019 | -1.200931 |
| H | -4.014722 | 1.613984 | -2.606059 |
| H | -3.251261 | 2.882762 | -1.639846 |
| C | 3.295976 | -2.452051 | -3.947270 |
| H | 4.261126 | -2.631378 | -3.480999 |
| H | 3.281871 | -1.476279 | -4.426294 |
| H | 3.082191 | -3.232453 | -4.676215 |
| C | -1.645734 | -1.293592 | 4.024498 |
| H | -1.007567 | -1.992957 | 4.573229 |
| H | -2.049733 | -1.808627 | 3.151006 |
| H | -2.479142 | -1.015073 | 4.676669 |
| C | 0.564662 | -1.466721 | -5.216781 |
| H | 1.059751 | -2.399495 | -5.456813 |
| C | -1.110199 | 4.434918 | -0.870043 |
| H | -1.794514 | 4.354887 | -0.022804 |
| H | -1.702448 | 4.611176 | -1.771903 |
| H | -0.482058 | 5.317447 | -0.722124 |
| C | -0.239522 | 0.614348 | 4.827156 |
| H | -0.998424 | 0.900608 | 5.560761 |
| H | 0.323506 | 1.508834 | 4.550913 |
| H | 0.444676 | -0.077907 | 5.325133 |
| C | 0.693090 | 3.333674 | -2.204793 |
| H | 1.381556 | 4.161927 | -2.011391 |
| H | 0.155422 | 3.548553 | -3.132781 |
| H | 1.283790 | 2.427977 | -2.353492 |
| C | -4.145616 | -0.319931 | -0.674280 |
| H | -3.690735 | -1.120321 | -0.089120 |
| H | -4.355548 | -0.699609 | -1.678632 |
| H | -5.097981 | -0.047461 | -0.209461 |
| C | -0.754304 | 0.414726 | -5.922525 |
| H | -1.254204 | 0.966041 | -6.710749 |
| C | -0.070716 | -0.756830 | -6.221298 |
| H | -0.041944 | -1.127652 | -7.239103 |
| **Transition State** | | | |
| N | 0.635967 | 0.481902 | -0.415715 |
| N | -0.441427 | -1.582490 | -0.910552 |
| H | -1.380210 | -1.643319 | 0.023683 |
| N | -1.238438 | -0.567319 | 0.770268 |
| N | 1.439883 | -3.111128 | -1.200578 |
| N | 2.289888 | -3.437587 | -2.144740 |
| N | 1.965697 | -2.720638 | -3.178508 |
| C | -0.258783 | -0.419187 | -0.171661 |
| C | 0.526999 | -2.158525 | -1.631842 |
| C | 0.981053 | 1.433605 | 0.540203 |
| C | -1.939623 | 0.530626 | 1.308586 |
| C | 0.650459 | 2.796408 | 0.345175 |
| C | 0.883865 | -1.928943 | -2.962026 |
| C | 1.767790 | 1.044207 | 1.656491 |
| C | -2.793825 | 1.302790 | 0.480266 |
| C | 1.612831 | -3.699383 | 0.090994 |
| C | 2.267941 | -0.386912 | 1.744361 |
| H | 1.426196 | -1.041963 | 1.469891 |
| C | 0.588130 | -3.683431 | 1.039262 |
| H | -0.369163 | -3.216903 | 0.821814 |
| C | 1.020702 | 3.728640 | 1.318107 |
| H | 0.743368 | 4.777890 | 1.191259 |
| C | 2.844532 | -4.294897 | 0.381552 |
| H | 3.639039 | -4.289922 | -0.363942 |
| C | 1.735495 | 3.342885 | 2.451349 |
| H | 2.004748 | 4.082200 | 3.210351 |
| C | 2.121600 | 2.011195 | 2.601126 |
| H | 2.707045 | 1.720009 | 3.476303 |
| C | 0.229881 | -1.068272 | -3.946321 |
| C | -1.814564 | 0.839861 | 2.684423 |
| C | -0.238519 | 0.195182 | -3.553364 |
| H | -0.037504 | 0.544000 | -2.536189 |
| C | -3.077293 | 0.857492 | -0.943723 |
| H | -2.127630 | 0.519102 | -1.386953 |
| C | 2.030691 | -4.870311 | 2.583781 |
| H | 2.193805 | -5.331044 | 3.560573 |
| C | 3.044699 | -4.883399 | 1.625184 |
| H | 4.007600 | -5.349206 | 1.845801 |
| C | -0.041762 | 3.199982 | -0.939549 |
| H | -0.730585 | 2.377147 | -1.187299 |
| C | 0.810792 | -4.266569 | 2.286410 |
| H | 0.011416 | -4.246668 | 3.030608 |
| C | -1.013029 | -0.073164 | 3.587429 |
| H | -0.164087 | -0.427129 | 2.984027 |
| C | -3.426048 | 2.425431 | 1.024332 |
| H | -4.072286 | 3.040752 | 0.395849 |
| C | -2.464600 | 1.972527 | 3.185914 |
| H | -2.354584 | 2.238825 | 4.239244 |
| C | 3.376555 | -0.616198 | 0.712309 |
| H | 3.014294 | -0.393380 | -0.301594 |
| H | 3.719943 | -1.662096 | 0.733935 |
| H | 4.243010 | 0.030939 | 0.924807 |
| C | 2.728573 | -0.822996 | 3.128539 |
| H | 3.631188 | -0.282011 | 3.454929 |
| H | 2.974989 | -1.896038 | 3.112853 |
| H | 1.947815 | -0.667663 | 3.887679 |
| C | -3.247633 | 2.774771 | 2.360479 |
| H | -3.733768 | 3.667368 | 2.763164 |
| C | -0.913749 | 1.000941 | -4.467373 |
| H | -1.275546 | 1.982786 | -4.152885 |
| C | -3.631590 | 1.955038 | -1.844013 |
| H | -4.639019 | 2.270902 | -1.529093 |
| H | -3.713584 | 1.588754 | -2.878698 |
| H | -2.984971 | 2.844586 | -1.850024 |
| C | 2.796305 | -2.795591 | -4.364340 |
| H | 3.805060 | -3.074848 | -4.042839 |
| H | 2.809720 | -1.807331 | -4.837815 |
| H | 2.398689 | -3.542111 | -5.063009 |
| C | -1.840586 | -1.303652 | 3.970247 |
| H | -1.243414 | -2.000691 | 4.580923 |
| H | -2.188712 | -1.843899 | 3.077270 |
| H | -2.724810 | -1.010263 | 4.560303 |
| C | 0.012473 | -1.509034 | -5.261446 |
| H | 0.340070 | -2.503133 | -5.571114 |
| C | -0.860927 | 4.479895 | -0.846766 |
| H | -1.607697 | 4.421361 | -0.040450 |
| H | -1.397290 | 4.651189 | -1.793425 |
| H | -0.229932 | 5.365338 | -0.668015 |
| C | -0.469189 | 0.616180 | 4.831745 |
| H | -1.277419 | 0.919024 | 5.516661 |
| H | 0.113146 | 1.512331 | 4.570370 |
| H | 0.189135 | -0.066689 | 5.390953 |
| C | 0.984997 | 3.288084 | -2.072683 |
| H | 1.710297 | 4.094840 | -1.877680 |
| H | 0.494140 | 3.496832 | -3.036844 |
| H | 1.540195 | 2.343878 | -2.170556 |
| C | -4.023273 | -0.348119 | -0.937701 |
| H | -3.629789 | -1.166465 | -0.317512 |
| H | -4.169833 | -0.733443 | -1.959603 |
| H | -5.009790 | -0.061482 | -0.538012 |
| C | -1.117186 | 0.564366 | -5.776707 |
| H | -1.639378 | 1.203417 | -6.493019 |
| C | -0.650085 | -0.690287 | -6.171524 |
| H | -0.809573 | -1.039746 | -7.194080 |
| **PhMII-DippCDI** | | | |
| N | 0.601285 | 0.359805 | -0.360101 |
| N | -0.381977 | -1.698748 | -0.878289 |
| H | -1.738024 | -1.411933 | 0.912561 |
| N | -1.220121 | -0.546974 | 0.873026 |
| N | 1.537417 | -3.074628 | -1.302414 |
| N | 2.332546 | -3.419272 | -2.293960 |
| N | 1.932764 | -2.710000 | -3.305779 |
| C | -0.279647 | -0.574480 | -0.146666 |
| C | 0.603514 | -2.118353 | -1.664877 |
| C | 0.931269 | 1.316152 | 0.594701 |
| C | -1.922381 | 0.604092 | 1.322737 |
| C | 0.635448 | 2.677633 | 0.372170 |
| C | 0.885610 | -1.902752 | -3.017641 |
| C | 1.707134 | 0.954182 | 1.719841 |
| C | -2.751327 | 1.315997 | 0.440860 |
| C | 1.737089 | -3.688898 | -0.036009 |
| C | 2.251668 | -0.452071 | 1.836200 |
| H | 1.479658 | -1.145931 | 1.494884 |
| C | 0.661225 | -3.896215 | 0.815906 |
| H | -0.327004 | -3.578482 | 0.518044 |
| C | 1.005300 | 3.619860 | 1.323787 |
| H | 0.751958 | 4.662166 | 1.167702 |
| C | 3.018855 | -4.088509 | 0.317846 |
| H | 3.845257 | -3.915189 | -0.358462 |
| C | 1.694457 | 3.254717 | 2.469086 |
| H | 1.964605 | 4.000578 | 3.208667 |
| C | 2.061346 | 1.930025 | 2.643027 |
| H | 2.638398 | 1.651364 | 3.517175 |
| C | 0.190281 | -1.042379 | -3.964426 |
| C | -1.838828 | 0.950733 | 2.678849 |
| C | -0.211357 | 0.227132 | -3.549449 |
| H | 0.042939 | 0.553723 | -2.545488 |
| C | -3.001430 | 0.812347 | -0.963853 |
| H | -2.056815 | 0.435043 | -1.362280 |
| C | 2.160267 | -4.895091 | 2.415258 |
| H | 2.327324 | -5.359314 | 3.380071 |
| C | 3.223458 | -4.695854 | 1.545341 |
| H | 4.223939 | -5.002794 | 1.826068 |
| C | -0.003468 | 3.097079 | -0.928484 |
| H | -0.681501 | 2.292143 | -1.222920 |
| C | 0.884120 | -4.496233 | 2.045520 |
| H | 0.047660 | -4.654072 | 2.716148 |
| C | -1.066396 | 0.076321 | 3.634809 |
| H | -0.204958 | -0.298309 | 3.078220 |
| C | -3.412129 | 2.441297 | 0.922402 |
| H | -4.040904 | 3.019801 | 0.257171 |
| C | -2.524004 | 2.077573 | 3.120200 |
| H | -2.457392 | 2.374143 | 4.159831 |
| C | 3.451544 | -0.590901 | 0.897180 |
| H | 3.164562 | -0.375302 | -0.133743 |
| H | 3.863598 | -1.601529 | 0.937933 |
| H | 4.238801 | 0.109953 | 1.190331 |
| C | 2.645790 | -0.869100 | 3.245532 |
| H | 3.503511 | -0.298682 | 3.612474 |
| H | 2.930180 | -1.924105 | 3.248229 |
| H | 1.826216 | -0.737908 | 3.955382 |
| C | -3.288513 | 2.830288 | 2.245487 |
| H | -3.806031 | 3.715027 | 2.600313 |
| C | -0.902660 | 1.054801 | -4.419378 |
| H | -1.204233 | 2.042078 | -4.090199 |
| C | -3.511437 | 1.874366 | -1.926372 |
| H | -4.515829 | 2.212965 | -1.658579 |
| H | -3.566410 | 1.456143 | -2.933697 |
| H | -2.855938 | 2.746539 | -1.957238 |
| C | 2.645670 | -2.851701 | -4.559596 |
| H | 3.650984 | -3.193831 | -4.328626 |
| H | 2.679375 | -1.883963 | -5.054783 |
| H | 2.142091 | -3.578133 | -5.195983 |
| C | -1.910239 | -1.130937 | 4.046597 |
| H | -1.330147 | -1.800679 | 4.687647 |
| H | -2.250272 | -1.702316 | 3.180466 |
| H | -2.793927 | -0.807841 | 4.604844 |
| C | -0.126423 | -1.475401 | -5.253588 |
| H | 0.142296 | -2.473485 | -5.577329 |
| C | -0.805061 | 4.386938 | -0.847725 |
| H | -1.561272 | 4.339628 | -0.061373 |
| H | -1.312921 | 4.569682 | -1.798490 |
| H | -0.163274 | 5.250854 | -0.654604 |
| C | -0.559231 | 0.805527 | 4.868786 |
| H | -1.379083 | 1.116554 | 5.521993 |
| H | 0.021729 | 1.689293 | 4.598560 |
| H | 0.084313 | 0.141518 | 5.451456 |
| C | 1.074082 | 3.214108 | -2.007609 |
| H | 1.777532 | 4.012097 | -1.751679 |
| H | 0.630765 | 3.451189 | -2.978905 |
| H | 1.634893 | 2.282802 | -2.104845 |
| C | -3.984259 | -0.359547 | -0.928628 |
| H | -3.633382 | -1.168447 | -0.285853 |
| H | -4.126299 | -0.765169 | -1.933886 |
| H | -4.956836 | -0.026795 | -0.554645 |
| C | -1.203485 | 0.626785 | -5.704898 |
| H | -1.743323 | 1.277334 | -6.383548 |
| C | -0.815645 | -0.640993 | -6.118272 |
| H | -1.058933 | -0.987137 | -7.116146 |


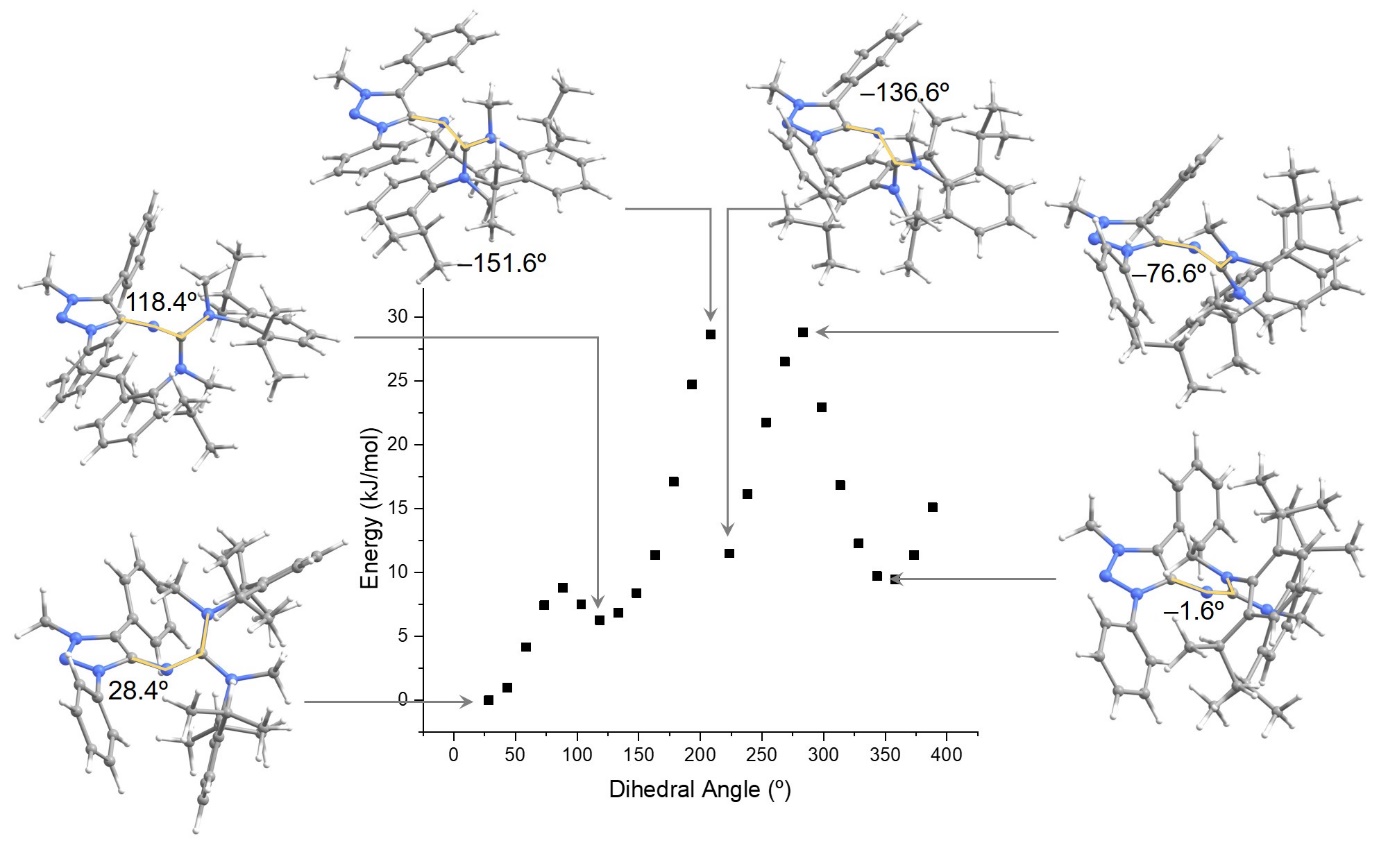


**Figure S42**. Energy profile for the relaxed surface scan of the N3-C7-N1-C8 dihedral angle of **N(I)**, starting from the optimized geometry (The atom numbering in this and subsequent figures corresponds to the coordinates shown in Table S13). The geometry convergence settings were not set up as Tight, and therefore the structure corresponding to nearly 360º from the starting structure is not exactly a minimum.


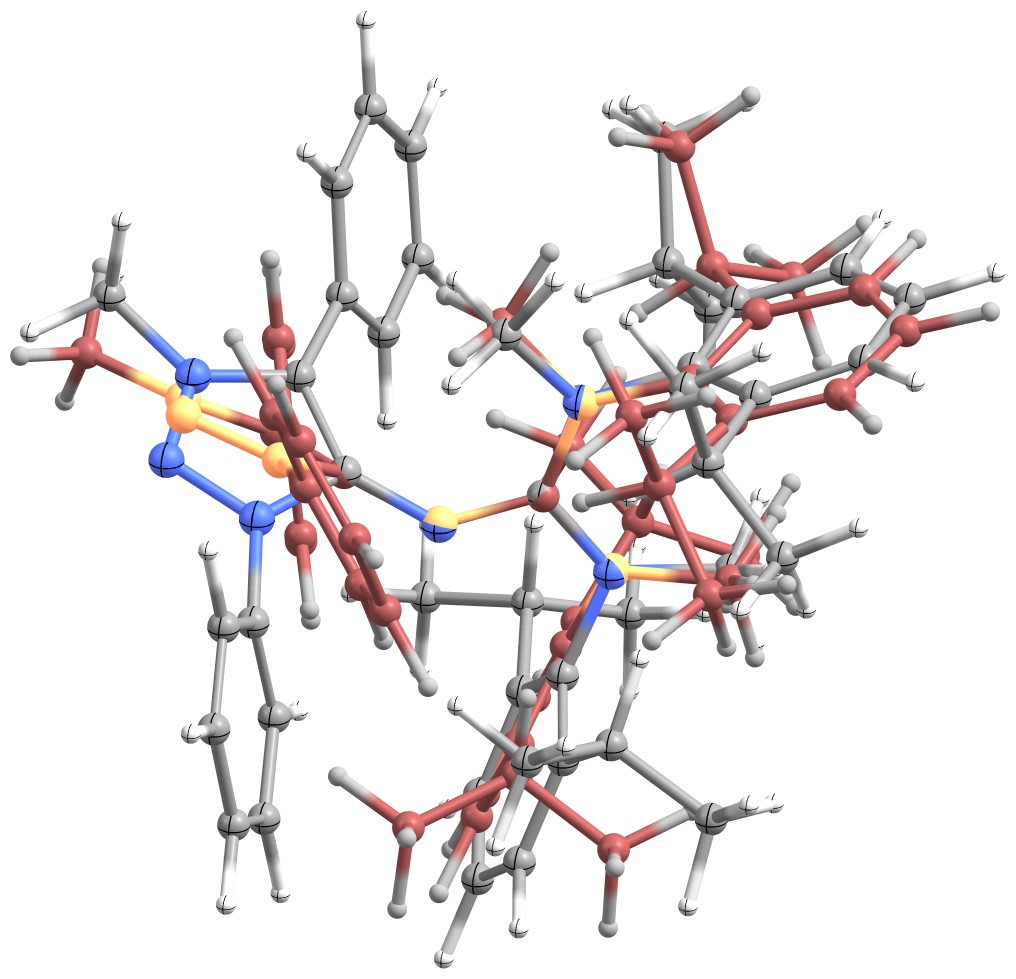


**Figure S43**. Superimposed initial (normal coloring) and final (contrasting coloring) structures from the N3-C7-N1-C8 dihedral angle scan of **N(I)**, showing the discrepancies in in side-group orientations leading to the energy difference. The four atoms which dihedral angle was scan are coincident in both structures.


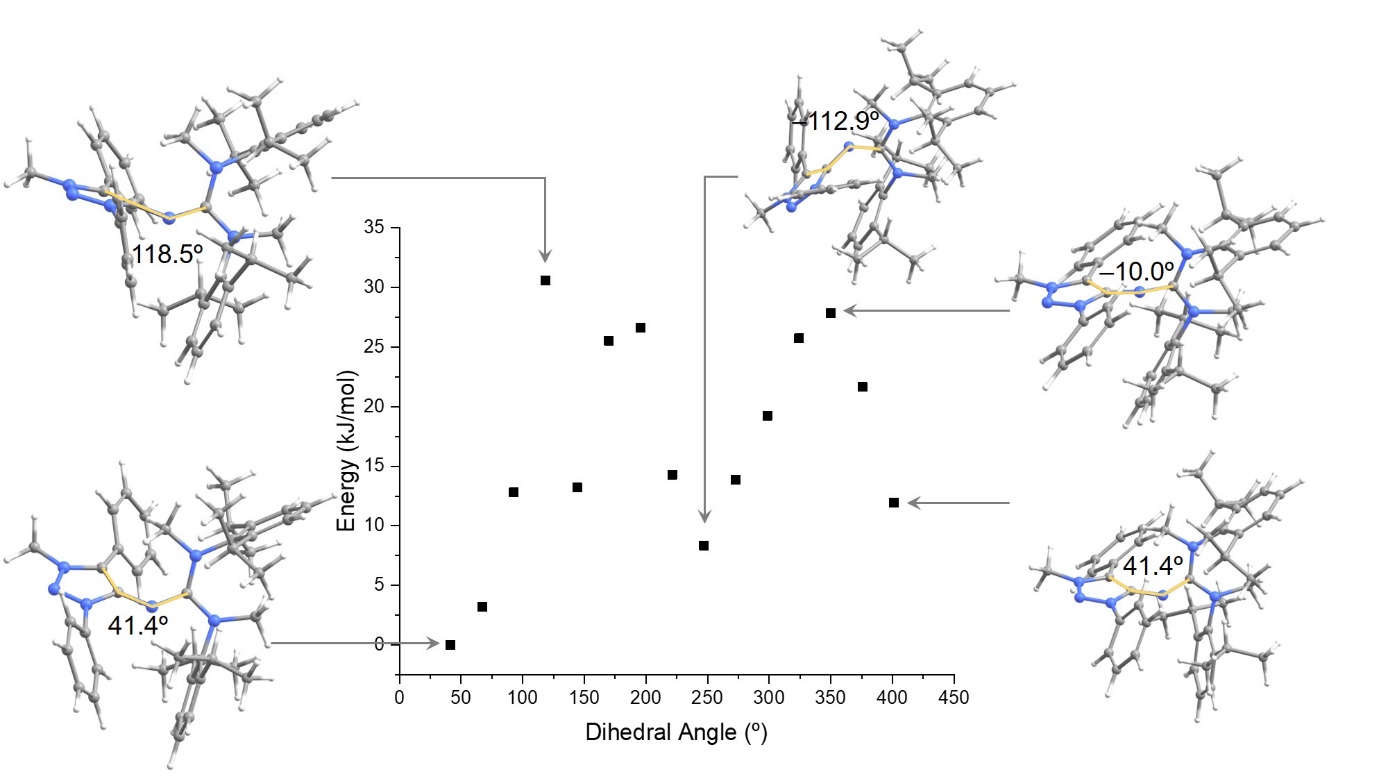


**Figure S44**. Energy profile for the relaxed surface scan of the C7-N1-C8-C19 dihedral angle of **N(I)**, starting from the optimized geometry. The geometry convergence settings were not set up as Tight, and therefore the structure corresponding to nearly 360º from the starting structure is not exactly a minimum.


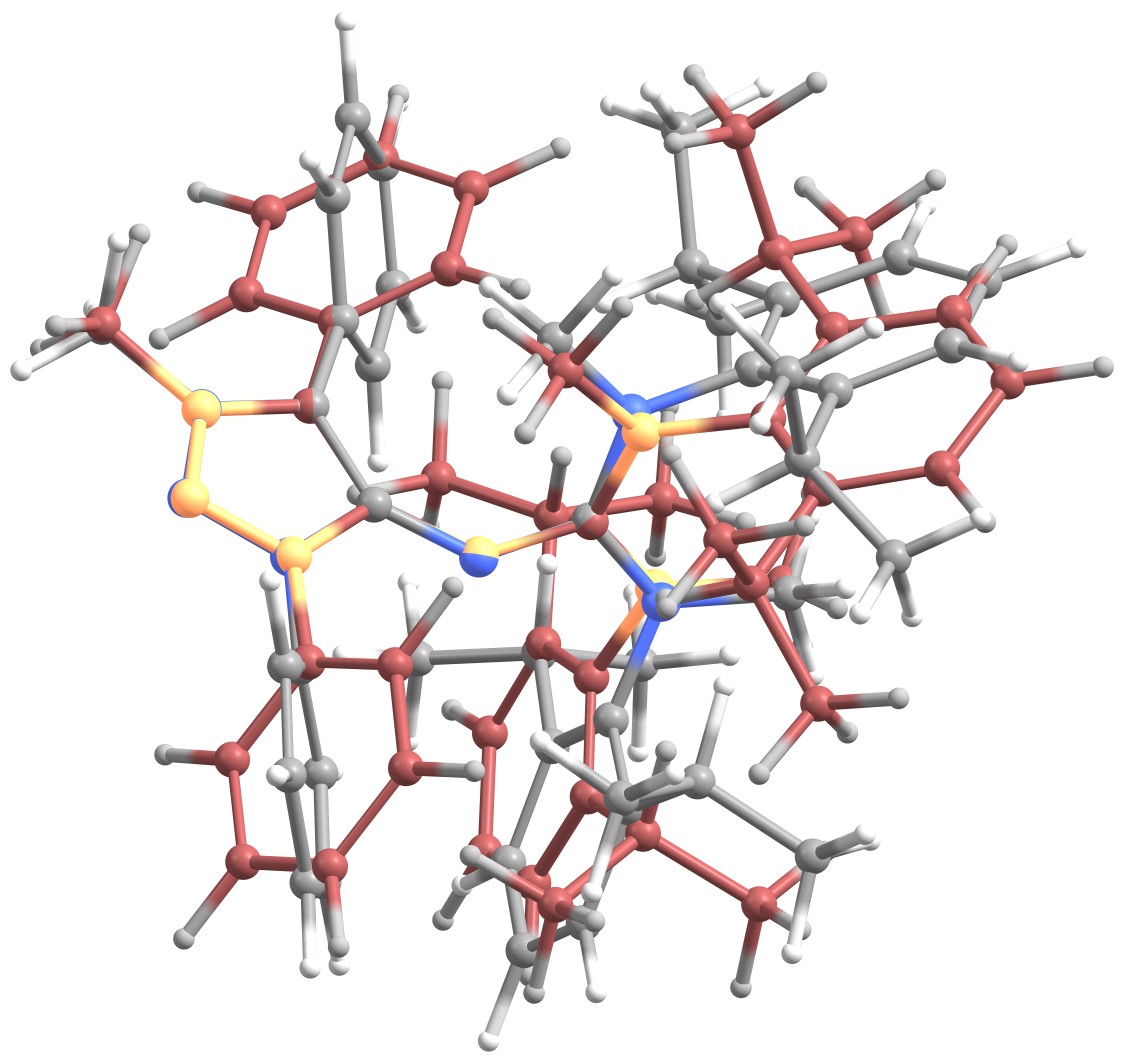


**Figure S45**. Superimposed initial (normal coloring) and final (contrasting coloring) structures from the C7-N1-C8-C19 dihedral angle scan of **N(I)**, showing the discrepancies in in side-group orientations leading to the energy difference. The four atoms which dihedral angle was scan are coincident in both structures.


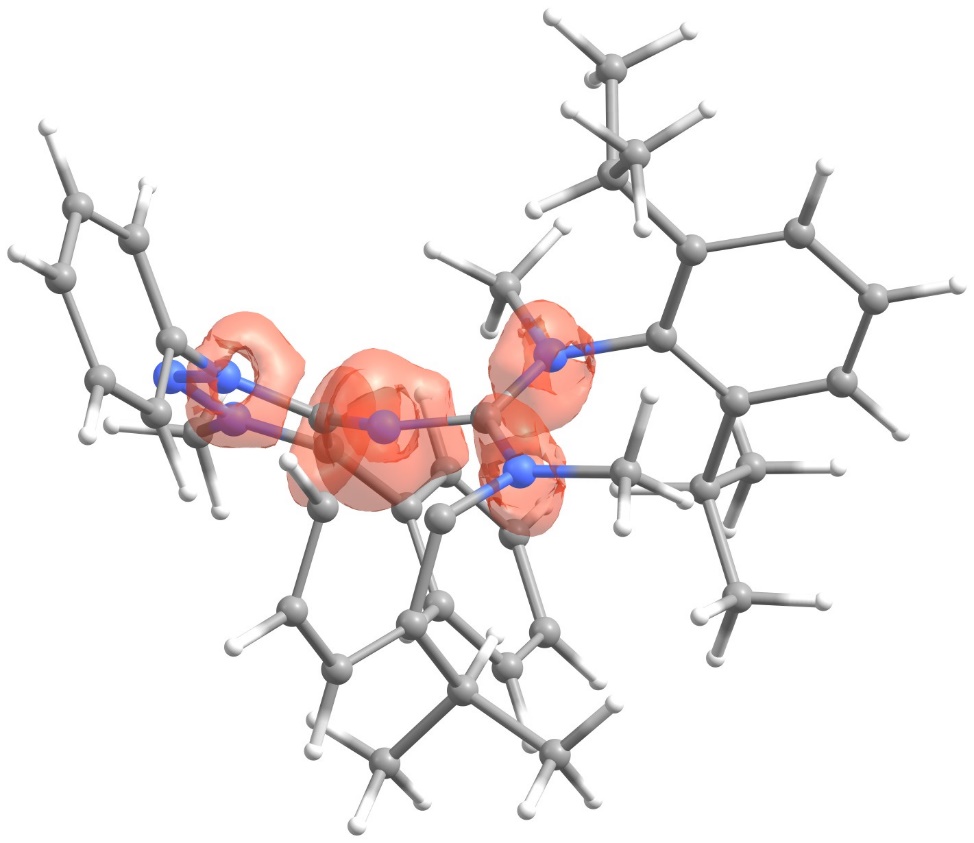


**Figure S46**. Electron localization function (ELF), calculated using MultiWFN, weighted by Hirshfeld weights of selected atoms (1,2,3,4,7,8,19). Isovalue = 0.7, Grid of 140 by 140 by 140 points. Some atoms in a dipp substituent were removed from the image to allow a clearer view of the isosurface. The coordinates employed in ELF calculations are given in Table S6.


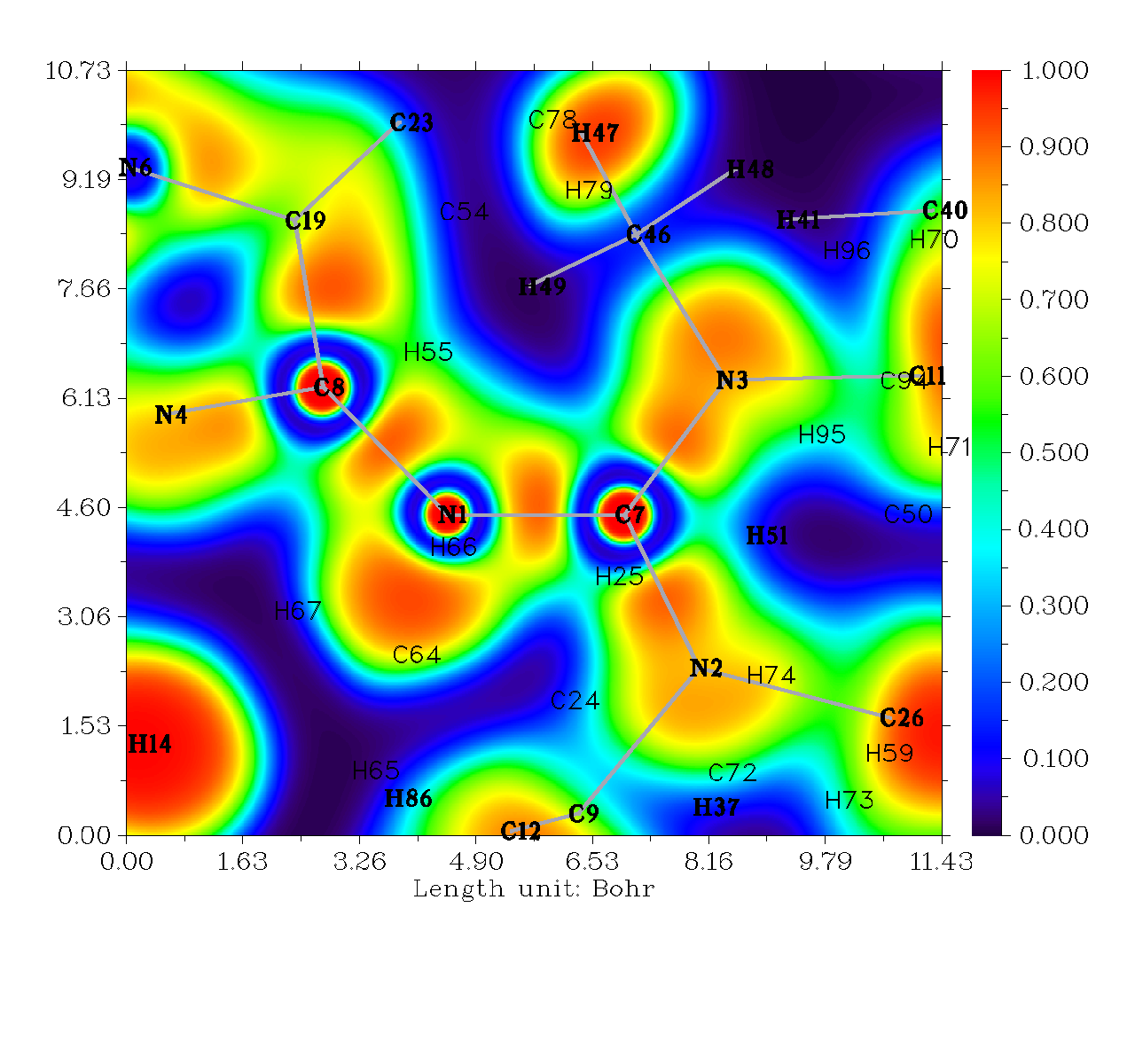


**Figure S47**. Heatmap of the ELF on the plane defined by the atoms 7, 1 and 8, calculated and plot using the Multiwfn program. The threshold for showing atom labels was 5 Bohr from the plane.


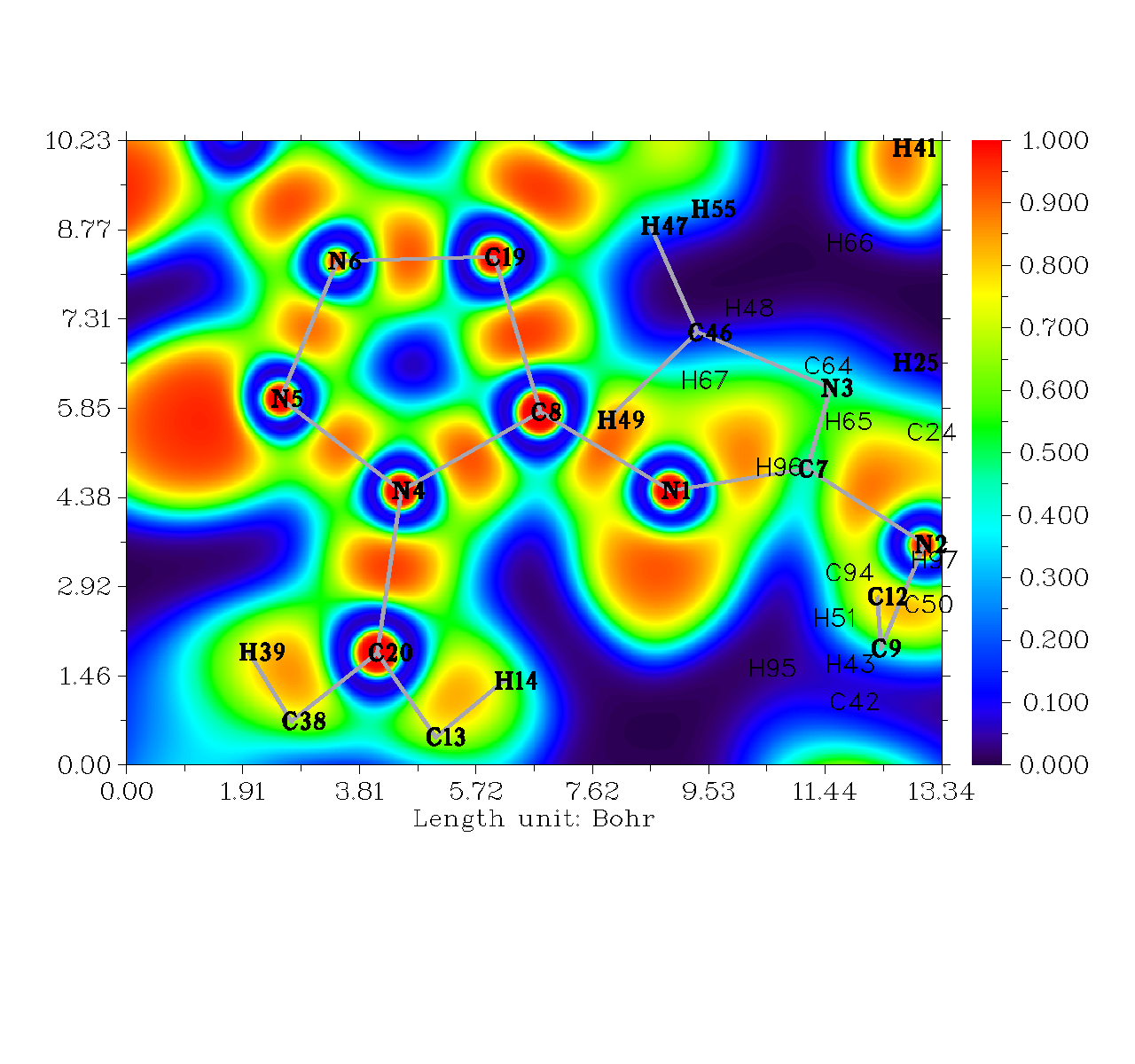


**Figure S48**. Heatmap of the ELF on the plane defined by the atoms 1, 4 and 8, calculated and plot using the Multiwfn program. The threshold for showing atom labels was 5 Bohr from the plane.


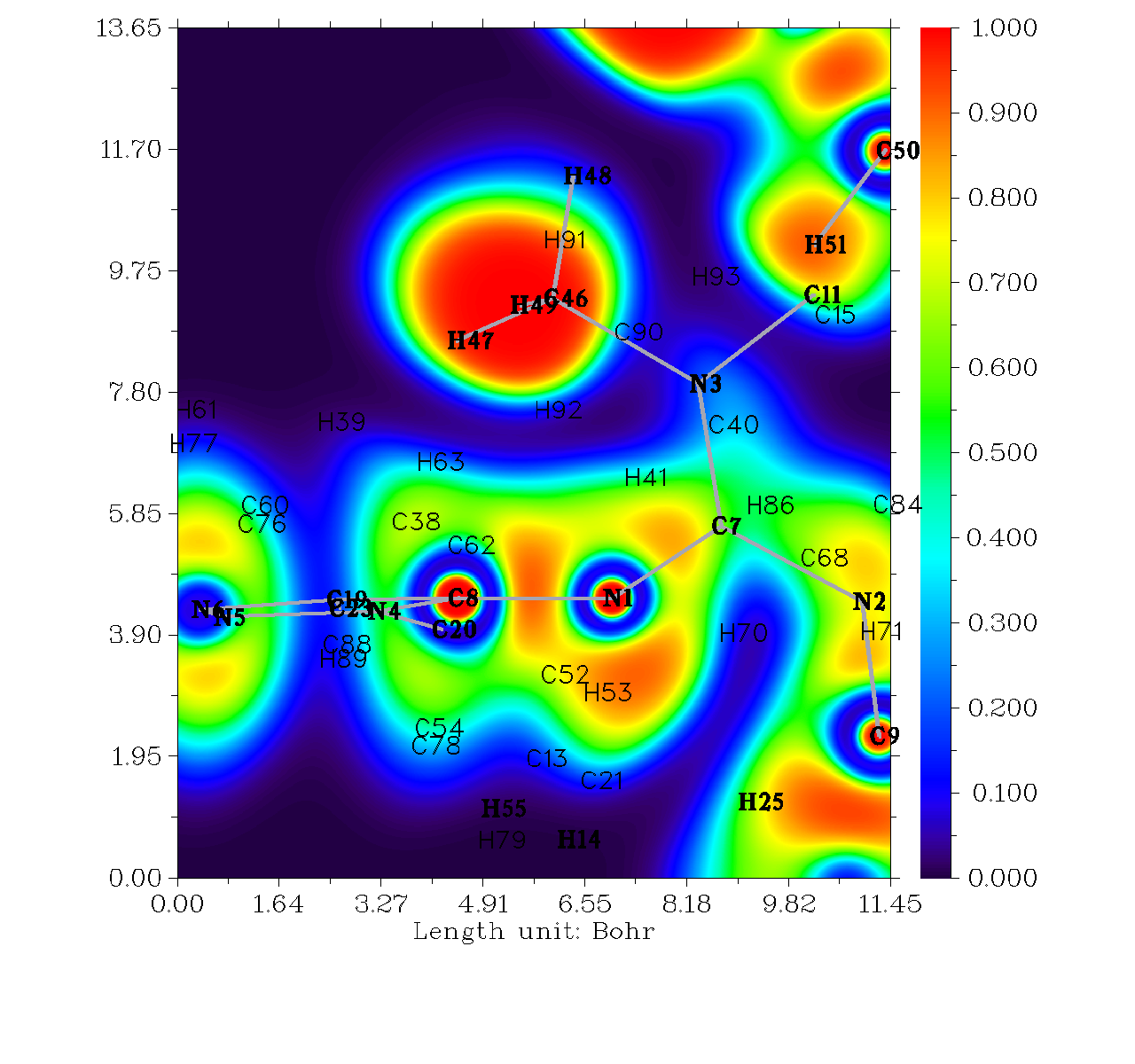


**Figure S49**. Heatmap of the ELF on the plane defined by the atoms 1, 8, 49, calculated and plot using the Multiwfn program. Atoms 49 and 65 are H atoms and they were chosen to approximate a plane perpendicular to the MIC group. The threshold for showing atom labels was 5 Bohr from the plane.


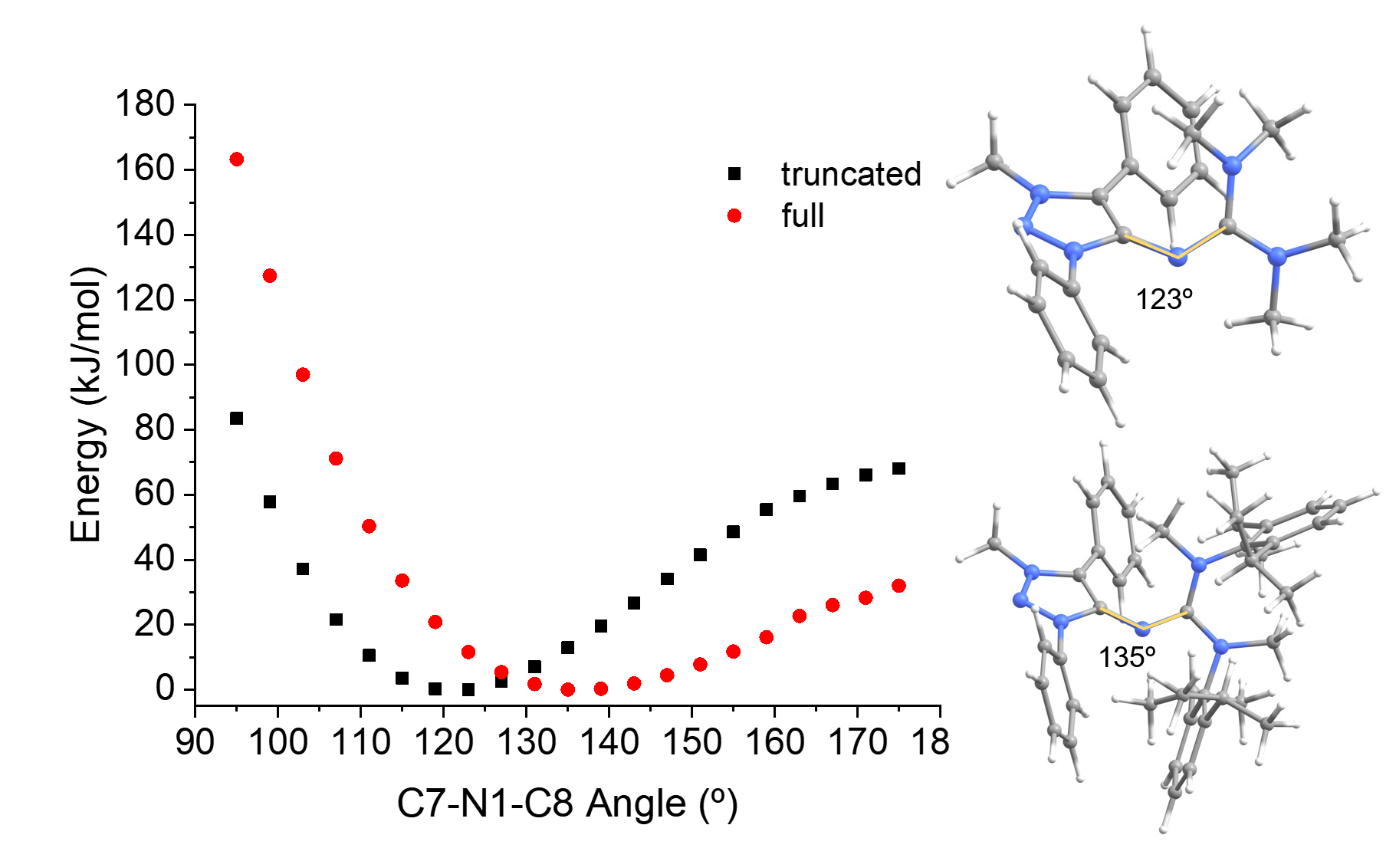


**Figure S50**. Energy profile for the bending of the C7-N1-C8 angle of **Dipp-N(I)**, and a truncated form with the Dipp ligands replaced by Me groups. The minimum energy angle for **Dipp-N(I)** is 135º, while for the truncated form it is 123º. The numbering corresponds to the coordinates given below.

Minimum energy geometry of the scan of the C7-N1-C8 angle of **Dipp-N(I)**

N 9.369136926087 21.354274235458 3.679178959487

N 11.290861421826 20.371125767654 4.327273807197

N 9.419705815984 18.996460275673 3.751199390672

N 7.699381064134 22.698484484549 2.738565349421

N 6.435712729765 23.042180747970 2.865593961786

N 6.000998708881 22.352733815174 3.870292292404

C 9.986597482817 20.220811846347 3.944284023045

C 8.109566244726 21.749525028627 3.662675972873

C 11.839205818523 21.701480642157 4.244988839763

C 12.479507140485 22.087750015390 3.053469481542

C 10.057443245726 17.724384708975 3.961086855445

C 11.790489830085 22.548899076855 5.367165291453

C 9.601885768358 24.106421021377 2.187996667719

H 9.879559196356 24.125020692199 3.243093877165

C 9.947825690093 17.101894851979 5.217023063499

C 10.699292752271 17.094123610560 2.874086933413

C 10.574242164886 15.862883615710 5.394089742395

H 10.522012002036 15.371121495670 6.368625684882

C 6.945379368992 21.546361883352 4.436048274712

C 8.501274253688 23.362299515069 1.765253974560

C 10.337646635719 24.810421016492 1.239173743690

H 11.202766917215 25.394618970624 1.558834450380

C 6.717308567627 20.809661909141 5.683314171448

C 11.167477499707 22.095342649179 6.670852698278

H 10.483599873321 21.266829932944 6.429675812649

C 12.268842930432 19.338759512786 4.649345859494

H 11.930714741763 18.690457277612 5.464591854800

H 13.176559015992 19.855845968624 4.981042441974

H 12.532712324959 18.711997688475 3.787112194293

C 11.293918523263 15.849198244053 3.096118344045

H 11.807084610344 15.345847895085 2.273594876829

C 11.248988033692 15.244000990739 4.348704751966

H 11.731503707919 14.276061758167 4.505399345248

C 13.050249349787 23.363381261577 2.993044047769

H 13.545797838416 23.684518750196 2.073218135486

C 12.591480537888 21.156603606723 1.863439043434

H 12.100914353006 20.214460414683 2.138690550052

C 8.133667024080 23.295346943982 0.423039538163

H 7.268765507997 22.698933938809 0.126155993093

C 9.161069558327 17.705939656579 6.362477776747

H 8.723701863981 18.651926537167 6.011092239051

C 12.374455182694 23.815153900923 5.260709950802

H 12.349135464268 24.492530197431 6.117057079920

C 12.994408714377 24.224654973937 4.082637053205

H 13.443182480536 25.219211656705 4.018905678002

C 8.104034320436 18.905730733404 3.131007739725

H 7.292919745752 19.100095474387 3.846226403223

H 7.978834244414 17.883834593550 2.759053044357

H 8.018517065599 19.597212847119 2.280651036269

C 10.751342569484 17.711206182045 1.489144477172

H 10.439561947821 18.763882939012 1.578492949763

C 9.976897077873 24.764750375594 -0.107886358621

H 10.559273029930 25.320105337420 -0.846766318954

C 7.554245829223 21.066367203469 6.776822595188

H 8.378087378385 21.768944129995 6.661476708188

C 12.159445721157 17.690396007889 0.894787796516

H 12.475495743232 16.665833199847 0.645552978045

H 12.903943961646 18.111992509693 1.585437553230

H 12.186862909746 18.279258507653 -0.034686653997

C 5.647050458061 19.914750533882 5.833572902345

H 5.000766808363 19.685583368430 4.983357772205

C 8.880457258249 24.004638922352 -0.515853117753

H 8.601783859422 23.962135042631 -1.571125016899

C 10.358720590652 23.194878319155 7.353865566094

H 11.004460023542 24.008883773531 7.717397780704

H 9.826751031240 22.790159996509 8.228820691764

H 9.615729825001 23.636280498394 6.671434334988

C 10.043477607330 18.026291677143 7.568512168401

H 10.525969649902 17.119492845118 7.966155020621

H 9.437453710020 18.465576701608 8.375157285629

H 10.834072091010 18.748407365681 7.315194085157

C 12.233500729019 21.541742974389 7.619852281533

H 12.794665002675 20.713693259589 7.163477867573

H 11.769165481888 21.166901946731 8.545543850746

H 12.955432746217 22.327683425833 7.894301964822

C 5.406560208080 19.315288956061 7.067039512515

H 4.569134312485 18.622493819762 7.176463174518

C 7.314291205413 20.460167771976 8.006564147913

H 7.968516824524 20.679100966327 8.853730607022

C 4.623510633601 22.529811298040 4.290254128824

H 4.264410204759 23.465873573361 3.849957623613

H 4.588395223332 22.583949756771 5.384720293238

H 4.012016697259 21.688693160207 3.939331253557

C 11.866666180572 21.688412470286 0.629579518923

H 12.310178387295 22.631708855767 0.274992856325

H 10.804330510546 21.876131881475 0.840853688260

H 11.931335065482 20.956551727325 -0.191027528326

C 6.234144740081 19.590732081716 8.156315107089

H 6.039710855450 19.120251669234 9.123057815308

C 8.009519874746 16.777700697453 6.752617807243

H 7.340535960113 16.592643755167 5.898210786575

H 7.413400159480 17.225931093928 7.559930979008

H 8.383847245001 15.804737120785 7.107995931995

C 9.770996645234 17.013442499129 0.541814343818

H 9.786328620051 17.495849410574 -0.447893506981

H 8.738449202111 17.042743281923 0.917958492977

H 10.050164493626 15.956232024544 0.407555307274

C 14.055623474831 20.840385607569 1.556843323855

H 14.130045163713 20.123260030170 0.725136776845

H 14.562484159280 20.404072510921 2.431317121370

H 14.606836148245 21.748406364405 1.265141067467

Minimum energy geometry of the scan of the C7-N1-C8 angle of trunc-N(I)

N 9.672018218924 21.528929705702 3.843997580724

N 10.922148211979 20.093688895189 5.111146140047

N 8.960363192548 19.303778913908 4.167400107453

N 8.242714963131 22.959999751219 2.614371485203

N 7.016982520032 23.451274358946 2.627295832648

N 6.452082904002 22.903874513658 3.658851632097

C 9.823705457515 20.327135653492 4.371532841380

C 8.480981375317 22.089970822213 3.649177150925

C 10.419495827496 23.711676356564 1.848046555916

H 10.761215049748 23.737376927147 2.873597924365

C 7.266717495419 22.065160431293 4.344314160902

C 9.121642363188 23.320604652189 1.557081722411

C 11.258794682484 24.065798051854 0.803534182975

H 12.274745908629 24.375128227016 1.017969108953

C 6.910434015031 21.347670362464 5.563503379817

C 11.597225637237 18.811756750350 5.125702822596

H 11.572296721356 18.353765821143 6.117718887033

H 12.641733014447 18.964590475946 4.841503129268

H 11.142767097492 18.135212635717 4.406546033735

C 8.647861574995 23.279645553638 0.254518860142

H 7.631151739187 22.962446200129 0.058891444645

C 8.125862067915 19.243063962678 2.990210920724

H 7.091528018837 19.531847289728 3.205200064919

H 8.121999766294 18.215898859997 2.617728925642

H 8.524356741425 19.890373415244 2.211249534727

C 10.800466566107 24.033677448382 -0.506828249862

H 11.462146913339 24.313029069816 -1.318251586567

C 7.767303475232 21.409343176601 6.663591395401

H 8.665887465634 22.012667490945 6.610033230774

C 5.748721447945 20.580353118177 5.643112772580

H 5.088700551015 20.504797682222 4.787039373710

C 9.496006654024 23.644646811702 -0.779084511852

H 9.136196765210 23.616734336815 -1.800599918438

C 5.447908391014 19.891139344655 6.807584215548

H 4.544408100210 19.295036282105 6.858822099245

C 7.465968357783 20.712740844199 7.821457680641

H 8.137266032238 20.767549995982 8.670434008022

C 5.082993897245 23.265841399991 3.971558066081

H 4.878382884242 24.219548676345 3.492901564838

H 4.981291230594 23.351882778145 5.051201154609

H 4.404131840236 22.502926324447 3.593162546765

C 6.305514099913 19.953237369713 7.896782759026

H 6.070590976040 19.409197885184 8.804171964356

C 8.697995766917 18.288881731786 5.166920622276

H 7.620043632675 18.243799856132 5.343255914856

H 9.038976265057 17.301766912439 4.842492236730

H 9.184270096055 18.549082085656 6.103604345218

C 11.693197655655 21.199533012373 5.633994588869

H 12.136045147983 20.897290829231 6.584974114802

H 12.498383655860 21.481769003779 4.947472166439

H 11.050886580462 22.061382651762 5.793890817679

**
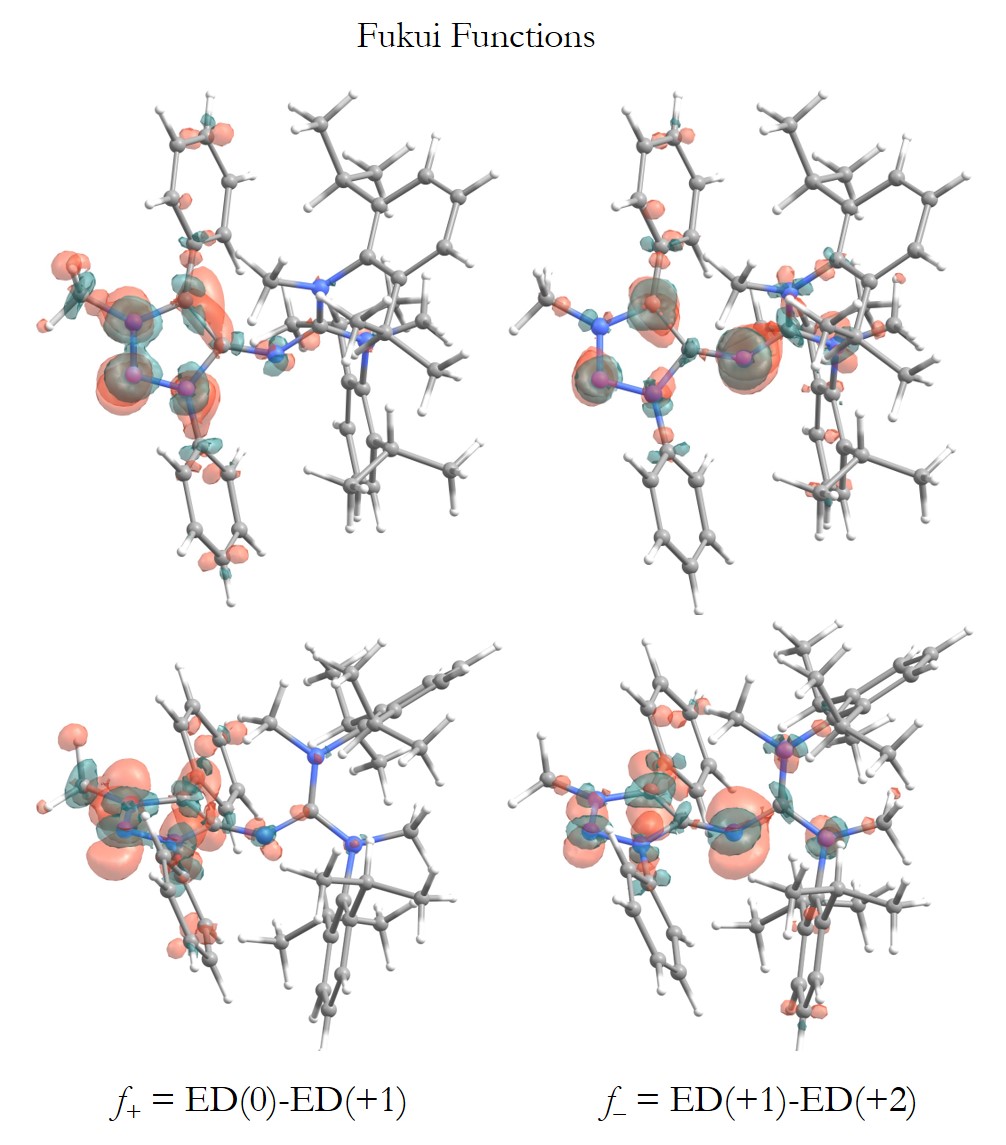
**

**Figure S51.** Fukui functions between the electron densities of the reduced and starting species (left, two views), and between the electron densities of the starting and oxidized species (right, two views). The *f_+_* Fukui function indicates the place of electron addition, and *f_–_* indicates the place of electron subtraction.


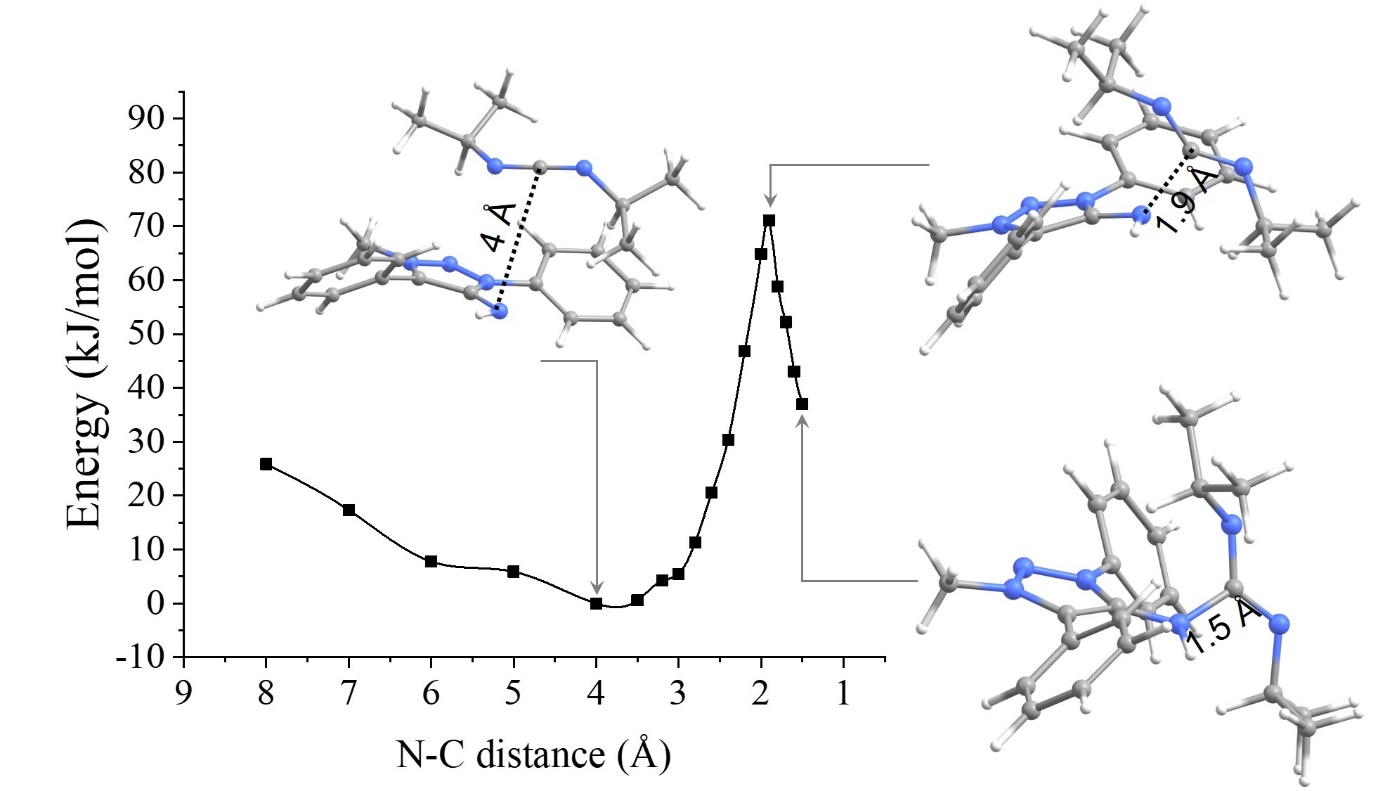


**Figure S52**. Energy profiles for the approach of the N atom in **MII** to the carbodiimide C atom of **iPrCDI**. Selected structures are shown.


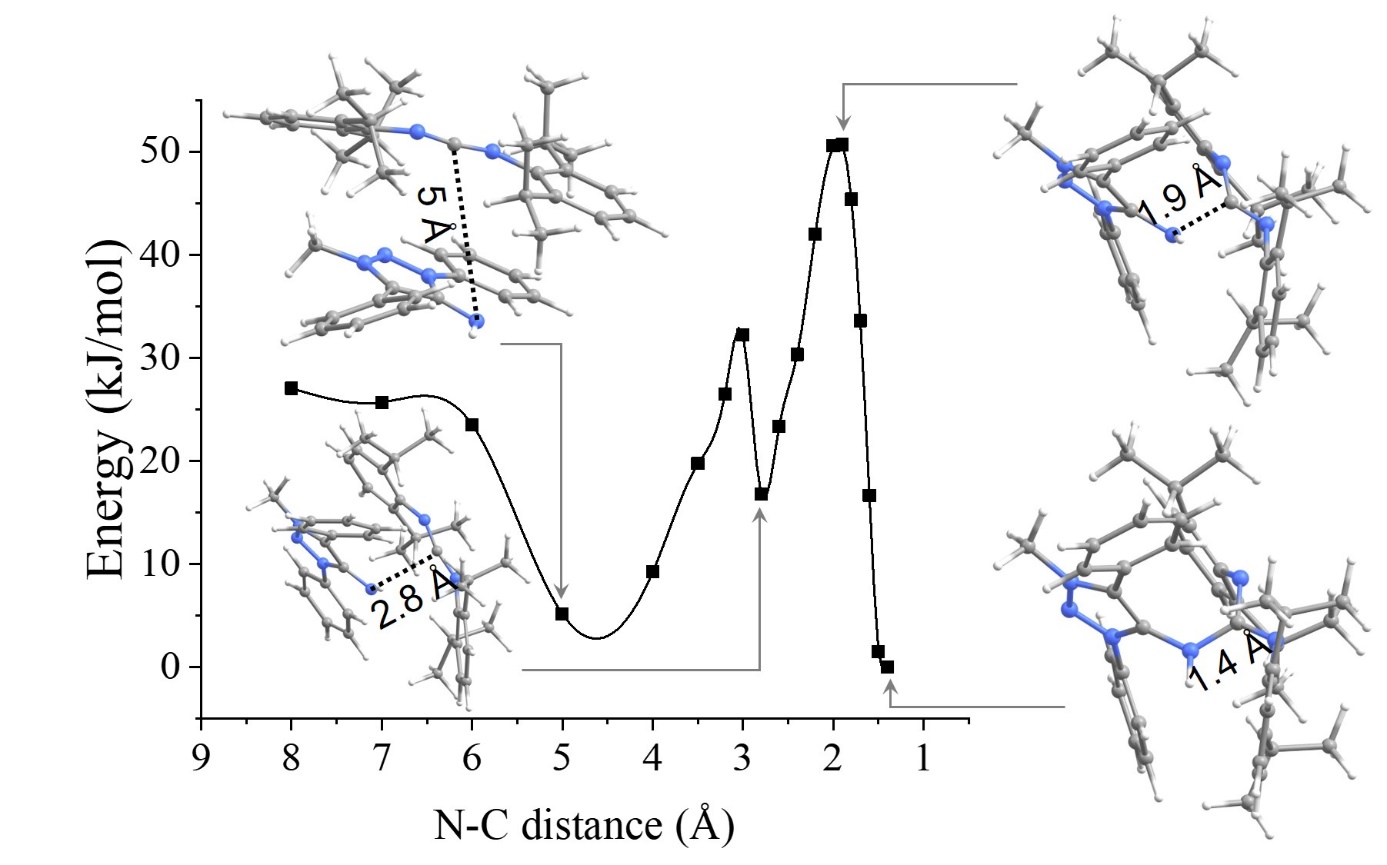


**Figure S53**. Energy profiles for the approach of the N atom in **MII** to the carbodiimide C atom of **DippCDI**. Selected structures are shown.

Structure 5 (4 Å) of the scan for the reaction of MII with iPrCDI

N 6.520280531023 5.849066681137 14.084543180213

N 8.534349421505 3.776795169037 10.759296329539

H 7.895027050422 3.434799696768 10.042782759313

N 6.936908022372 7.126940825473 12.052897633219

N 9.875036959287 5.709430755946 11.171449134108

N 10.199640096764 6.871608659924 10.592663267551

N 9.540428550967 6.883726209179 9.476594534910

C 6.795437164288 6.515266028937 13.099587325370

C 8.976771464892 4.942318710828 10.408018684201

C 8.774705165144 5.788877329639 9.264848538181

C 10.333374649108 5.461591536916 12.480653769252

C 10.493996173744 4.149459308308 12.939125710708

H 10.235254422607 3.322758894296 12.280046503898

C 10.625553750448 6.544795715680 13.319889927677

H 10.486504472890 7.562342256553 12.953409719098

C 7.872879962531 5.570402687299 8.140730530219

C 6.583645273699 5.068374843575 8.399433860896

H 6.278495677893 4.886918633944 9.432823579980

C 11.243006789878 5.003319483891 15.080961639224

H 11.593593874376 4.823765414958 16.099967297014

C 11.081966259543 6.309261840549 14.613650537814

H 11.304327016822 7.157593550371 15.265403563107

C 10.946912245284 3.931631318516 14.239258687266

H 11.067010885126 2.905470236865 14.595287725113

C 5.692830381251 4.829227305167 7.357063592557

H 4.694375764519 4.443570161227 7.578182713109

C 9.686878415202 8.040656710653 8.616937694915

H 10.022576419060 8.876856821292 9.239879173649

H 8.717113098074 8.273555023856 8.161366655921

H 10.426853595907 7.839266446339 7.830993931899

C 8.241676659131 5.821211449918 6.807728391525

H 9.247852317406 6.176768962642 6.577016919735

C 6.066152472191 5.092987218400 6.038352875728

H 5.365235345085 4.907312895425 5.220953011175

C 7.341688373816 5.591351558963 5.769981136803

H 7.645650815301 5.791754267254 4.739550287791

C 7.396557154043 4.914713756178 14.791419373506

H 8.445504612370 5.152771838100 14.549747290120

C 7.106030087419 3.499066171151 14.314995112395

C 7.197075592630 5.076623135508 16.288271211062

H 7.424442843189 6.105497949813 16.605162214255

H 6.156072034582 4.850619400530 16.569239830070

H 7.862265504981 4.392330959428 16.836199364504

H 6.054084759000 3.233418694720 14.506344362572

H 7.748927496891 2.781877288449 14.847895409029

H 7.307767967309 3.406734725728 13.237100564795

C 7.406359879779 8.489068839474 11.839316837422

H 8.459939628970 8.402308384559 11.520460219390

C 7.359035400433 9.333096819298 13.103190890033

C 6.621202814289 9.106503817698 10.692472268898

H 6.678192472930 8.470014830976 9.796785987235

H 5.560142853404 9.221131182283 10.965255699696

H 7.023916339051 10.098802534773 10.441143882285

H 6.323562473013 9.432963048799 13.466639722377

H 7.753784639411 10.341115870011 12.906593242459

H 7.964184183229 8.879179054352 13.903242042603

Structure 14 (1.9 Å) of the scan for the reaction of MII with iPrCDI

N 6.830445242881 5.080025649496 13.995459343414

N 7.958859484286 5.229220595573 11.665636668533

H 7.235621024898 4.820986173004 11.077650758382

N 6.442263499550 7.074891246623 12.782089935755

N 10.049309530507 6.382816255936 11.361529277370

N 10.763460437001 6.928323239912 10.377114810718

N 10.081721863989 6.703368889202 9.299170192450

C 6.919483330578 5.946785909718 13.085072131843

C 8.856348617496 5.825022330329 10.912569797865

C 8.918586880398 6.039047034374 9.507202852785

C 10.536243037896 6.499354554618 12.687552426265

C 10.214726546636 5.534894637076 13.643720341923

H 9.575262378624 4.696275898864 13.376005260814

C 11.354424547400 7.585604611418 13.014173579463

H 11.588642826906 8.333119089956 12.255789015501

C 7.915613336300 5.701170240978 8.502304050829

C 6.555634100890 5.901903803439 8.802910067167

H 6.268214911333 6.327702624817 9.767159386876

C 11.526074306309 6.750638218649 15.277464805934

H 11.909284521973 6.850921897723 16.295509644295

C 11.848966307069 7.702950777997 14.309357320252

H 12.484807645905 8.553695448279 14.565206031877

C 10.711249504480 5.671331043387 14.938388487353

H 10.456142568724 4.918221097808 15.687600575445

C 5.571752692506 5.576000788355 7.873699311360

H 4.520756749561 5.741827838482 8.122280003932

C 10.613277888975 7.192187857663 8.041768559453

H 11.309747341485 8.005318433815 8.271697147252

H 9.786393110391 7.561180052876 7.423597858630

H 11.139993601394 6.385238850382 7.515568637741

C 8.259758890015 5.159328609652 7.252671092005

H 9.305230756804 4.957326016234 7.012836184570

C 5.925055037870 5.052360746781 6.629570770982

H 5.151736238164 4.799765703236 5.900214031908

C 7.270740584509 4.846253883449 6.323774442055

H 7.554851558811 4.424771736294 5.356507446181

C 7.069248918134 3.668617536934 13.858882429307

H 7.923045230384 3.508971740502 13.171278665564

C 5.859805225949 2.960897848651 13.248266794975

C 7.421352826203 3.080339929951 15.218985158048

H 8.305342450513 3.581459943617 15.642283796918

H 6.586488592622 3.218842515772 15.925142831967

H 7.635398728576 2.003100006076 15.143257026103

H 4.969155359495 3.108432236848 13.880483654299

H 6.039057254944 1.878243802383 13.151517973412

H 5.632631558943 3.360968623704 12.247604208587

C 7.066597630368 8.166785656824 12.090252066276

H 7.722494165555 7.791102417131 11.280783988213

C 7.935723981577 8.991695329795 13.039411732098

C 5.996466131875 9.032083965950 11.434888660175

H 5.402966068631 8.446016606919 10.716406128584

H 5.307512354394 9.428116426533 12.198285907891

H 6.446651341627 9.881940538454 10.899153794912

H 7.315518455390 9.427861253884 13.838943435571

H 8.442816945402 9.811439818635 12.505603173577

H 8.704084151380 8.362124926588 13.510552671722

Structure 18 (1.5 Å) of the scan for the reaction of MII with iPrCDI

N 7.005288398877 5.148963883383 13.940496769705

N 7.751764700080 5.520488676598 11.705775604062

H 7.046983709220 4.992545140669 11.200995395764

N 7.474906378402 7.327591988984 13.274876441099

N 9.975404182437 6.409897993058 11.424326556666

N 10.780928219921 6.801607048961 10.447582026536

N 10.123590873621 6.573046039503 9.354567405443

C 7.376954389615 6.037198395724 13.063173222983

C 8.764461444682 5.964793692416 10.956031972910

C 8.883470233584 6.056132394935 9.560097792931

C 10.452975311718 6.475012107288 12.764973255748

C 10.333532055034 5.356174865232 13.586349730912

H 9.855849009839 4.449139102308 13.215005718375

C 11.090551636111 7.636643748793 13.198987437349

H 11.176812215023 8.491390158578 12.526408461438

C 7.896415036538 5.737861002097 8.527519680755

C 6.578840641159 6.199802683252 8.678160289413

H 6.313742824425 6.812131576063 9.542488693186

C 11.464581544969 6.579662867908 15.339734162478

H 11.857184640743 6.622448679966 16.358259152940

C 11.598382759313 7.682453116788 14.493584470778

H 12.094608654895 8.589470757099 14.846043196031

C 10.836858393274 5.421012292256 14.883534382403

H 10.741467455273 4.552476866089 15.538952577893

C 5.616418638259 5.897467878140 7.719015877339

H 4.595595049096 6.265913049499 7.844234055564

C 10.745279510609 6.900358109800 8.085391790519

H 11.508370283709 7.661929911934 8.277190310154

H 9.979092105411 7.289350619147 7.404804850962

H 11.208143216459 6.003560964019 7.653502386354

C 8.229712670757 4.965983832695 7.404168526345

H 9.243375094294 4.576435908443 7.289168830794

C 5.956556455801 5.137193080837 6.599482559249

H 5.200294334594 4.902522947204 5.846569454615

C 7.263665849188 4.674524543918 6.444315922266

H 7.532782857440 4.072112676864 5.573568478587

C 7.116418069923 3.736360780841 13.685822212864

H 7.887743458949 3.509047776641 12.917922202524

C 5.790334061027 3.172147888379 13.166206587886

C 7.528540044114 3.021008011349 14.970432240161

H 8.497663930853 3.399353417227 15.330261779156

H 6.784544232316 3.208416385082 15.762080197489

H 7.612535586865 1.932123048747 14.823640014448

H 4.984950357143 3.382878338934 13.888890805545

H 5.841337663804 2.082044311685 13.010248912335

H 5.504363007546 3.639412094415 12.209735469114

C 7.297075465835 8.284152328940 12.218047073698

H 7.840098744743 8.022627908621 11.279824325804

C 7.849013745946 9.634679816920 12.663003108270

C 5.820155098909 8.412809011315 11.830661767539

H 5.413865952478 7.438815180577 11.513278750721

H 5.230884562125 8.745865057036 12.700974999552

H 5.668989360259 9.133529667900 11.009420656847

H 7.325420970471 9.970925947937 13.572894667200

H 7.725084603425 10.403673840483 11.883779085705

H 8.919178583367 9.553703498069 12.906344049967

Structure 4 (5 Å) of the scan for the reaction of MII with DippCDI

N 7.751074230244 9.827040610745 13.848307038440

N 7.453049218008 5.603999604571 10.394283003600

H 7.196096351888 5.884716764835 9.448954107424

N 6.647447157432 7.862909586234 14.715091284575

N 8.989287889967 6.304821155791 12.095918023849

N 9.912436125199 7.254729642216 12.297829770599

N 9.911164657390 7.966017694221 11.216039915160

C 7.238336600189 8.799060049090 14.234268353187

C 8.360577966990 6.415133101244 10.839634524775

C 8.928621903212 10.529670055212 13.707219021009

C 5.730319549740 6.846663541961 14.552998769122

C 8.922876956558 11.624361370483 12.813152129815

C 9.021386915480 7.563409309717 10.280208191741

C 10.080896843330 10.184397308431 14.453794385859

C 5.600675476541 5.922146385451 15.613529432533

C 8.816273059306 5.322251069597 13.093888783387

C 10.038159343328 9.023716124634 15.424726006221

H 9.442204922040 8.230440396947 14.944174387937

C 7.853653011213 4.315404951714 12.953185751965

H 7.228632535726 4.312722512234 12.062114021761

C 10.098483898695 12.363228739509 12.668161534532

H 10.120212461695 13.210753062633 11.980436230453

C 9.637674132535 5.352214102774 14.231129430534

H 10.389401800372 6.132586990495 14.334950980728

C 11.244461855087 12.038106024504 13.390808798235

H 12.155696767202 12.628080510145 13.266056260854

C 11.229041287082 10.960887938803 14.274783827961

H 12.133564170047 10.716014921025 14.834574908547

C 8.784692086701 8.189441731150 8.986081740840

C 4.940726801002 6.759420277639 13.382129042243

C 7.461621041763 8.309246748659 8.521806641166

H 6.639838324111 7.955239140285 9.146315955494

C 6.426873746254 6.112605622506 16.869852689518

H 7.443984625481 6.380099079945 16.534262979364

C 8.549526507047 3.359026409025 15.070852678905

H 8.450685228131 2.587859469754 15.838077281667

C 9.496399394049 4.375583270436 15.210671476544

H 10.142465613308 4.408415935248 16.091583352225

C 7.662239627241 11.932605541572 12.031976376625

H 6.821912732142 11.744102145095 12.722124715878

C 7.732403230728 3.341714890573 13.943054126531

H 6.978929838007 2.559200365931 13.824367422927

C 5.040010557641 7.829117340525 12.313097212390

H 6.113402214552 8.043768095602 12.164391771257

C 4.682491022206 4.881770996746 15.467680935148

H 4.567988824044 4.147897419238 16.267289495476

C 4.043368142210 5.692935289067 13.285303046580

H 3.428961459930 5.588559057228 12.389572717165

C 11.404014763295 8.416398393741 15.718559791815

H 11.927623961771 8.135151172048 14.792004864700

H 11.285250555752 7.510255492996 16.332079184595

H 12.050697681049 9.108278201002 16.280696902784

C 9.328341284566 9.421855390115 16.722609936438

H 9.919012468786 10.176484127468 17.266095850565

H 9.201322796103 8.546765205559 17.379252632025

H 8.332644925181 9.848908214431 16.532766042387

C 3.915511806587 4.759436889179 14.311110604186

H 3.208265707561 3.932543995854 14.209571058564

C 7.187784984783 8.906241291011 7.295157866712

H 6.151614775801 8.996623309659 6.959627372131

C 6.533917632111 4.864529564098 17.735581966728

H 5.563803602929 4.596762491256 18.184245241997

H 7.238047093262 5.042451043321 18.562676771737

H 6.895850663198 4.000356733792 17.160731554792

C 10.837490658031 9.077693245920 11.138085648913

H 11.079389588999 9.383384186839 12.161024005246

H 10.359740653932 9.907160009610 10.606298602067

H 11.748879817039 8.768627214116 10.609486535332

C 4.359787414133 9.121643422507 12.780670346929

H 4.534765818793 9.932865625505 12.056567070001

H 4.724962099413 9.459127543137 13.761137716823

H 3.272381118934 8.966574739045 12.865259416161

C 9.824426694133 8.671531225464 8.171409104012

H 10.863649165634 8.553444984969 8.482041734052

C 7.567168776474 13.377966705289 11.560624724526

H 7.698060620739 14.087918610235 12.391619883120

H 6.580621674196 13.560990295583 11.108318276912

H 8.323783131616 13.608464798001 10.793634762760

C 4.485445557981 7.406464940254 10.959287138294

H 3.400871914848 7.220213379912 11.001460808970

H 4.989378492712 6.501664423251 10.591149628073

H 4.646396920210 8.211981805947 10.226737353074

C 7.500973614707 10.963654049131 10.858053241590

H 8.300072226051 11.112012950527 10.113814990928

H 6.536696443623 11.121412559250 10.349398108839

H 7.538130484167 9.916434242898 11.190974536276

C 5.890666579362 7.287309475607 17.694814495453

H 5.848468461490 8.211757215231 17.101635366020

H 6.535953201816 7.472577319542 18.568246740497

H 4.875465845728 7.066930664236 18.062558699530

C 8.227004541181 9.400663490417 6.505657152446

H 8.011642071957 9.874323261832 5.544848132610

C 9.544050562181 9.279383023899 6.949965397200

H 10.366453756014 9.650140529792 6.333138647717

Structure 9 (2.8 Å) of the scan for the reaction of MII with DippCDI

N 7.497082016092 9.663800465266 13.437857381901

N 7.895018493348 6.638047710201 12.425122231420

H 7.122949714108 6.852257313047 11.796761992630

N 5.824868580665 8.113213315762 14.231994158671

N 10.279861992401 6.784860102497 12.464966800019

N 11.276081000869 7.318255511405 11.752709996954

N 10.714789198724 7.821542523156 10.702754480657

C 6.732438815642 8.784863849737 13.796103272705

C 9.015105092552 7.002450324250 11.882934369143

C 8.849405097212 9.970439850722 13.511721319290

C 4.999072272666 7.034314238143 13.961687900077

C 9.374697448842 10.802544927307 12.497230172157

C 9.368239888818 7.671625103885 10.658194867228

C 9.657173495637 9.510450325932 14.579920441094

C 5.088208141869 5.896440803663 14.792286648848

C 10.619857742879 6.071171960440 13.633394510542

C 9.033642401851 8.763524053037 15.742146223923

H 8.395414454488 7.971513232086 15.314520251848

C 9.663284376054 5.320056689400 14.326498044479

H 8.633853011502 5.328055833343 13.970608032777

C 10.742269245069 11.084384885376 12.513318914765

H 11.171833136995 11.708448152098 11.726943542647

C 11.951202659292 6.091841359325 14.076613585620

H 12.690064790745 6.681777011180 13.536155729798

C 11.564320416378 10.588534718301 13.522308507704

H 12.634485352198 10.809951794881 13.515512012629

C 11.017903457120 9.826916858875 14.552605116224

H 11.666478547711 9.467286603849 15.352923982626

C 8.501422402239 8.090045252815 9.566563226461

C 4.057216919868 7.117968094415 12.911676701493

C 7.214632704250 8.582729339637 9.849539400102

H 6.892931247812 8.674116812066 10.887082701450

C 6.074911139663 5.884924444378 15.941754615848

H 6.993368988546 6.367396464919 15.569111392237

C 11.372430251140 4.598905949684 15.892340196485

H 11.664500371734 4.024119922824 16.774160475798

C 12.317504230499 5.357933838603 15.199471280464

H 13.356766640154 5.383503066617 15.536384375196

C 8.436823985757 11.429532829218 11.486487198445

H 7.637244398313 10.698590810663 11.300881454308

C 10.052068796860 4.587844717483 15.448080752247

H 9.301335364330 4.000233570538 15.980468626200

C 3.935990161033 8.392999184777 12.101180976168

H 4.960503575535 8.747231532051 11.896584986803

C 4.230279340611 4.825511654947 14.532972811464

H 4.286462482724 3.929124616539 15.153176901009

C 3.222181452006 6.018370932276 12.694773958603

H 2.490240887363 6.050915117565 11.885872055816

C 10.036270612659 8.074482805889 16.656797307625

H 10.705436293969 7.402824372695 16.103227201050

H 9.499761808717 7.473814754729 17.406759012335

H 10.653729321028 8.806252740931 17.202049095514

C 8.143180864700 9.701034845910 16.567198791680

H 8.759978419495 10.474236435032 17.052557311692

H 7.619275432999 9.138298208344 17.354917653670

H 7.386639273617 10.211459330905 15.954883000001

C 3.306064490495 4.880192187516 13.492400761513

H 2.645530858453 4.030601595161 13.302500036201

C 6.366813545382 8.999544842020 8.827892576651

H 5.379236078780 9.393289705111 9.076960934018

C 6.449406298201 4.487769011236 16.417478312726

H 5.594576433981 3.970233906928 16.881123466202

H 7.241687802197 4.551835107171 17.178561582400

H 6.818496689465 3.860900115921 15.591202598379

C 11.578840057826 8.501792305153 9.758288136627

H 12.460662383375 8.843866245223 10.310269915022

H 11.046746327009 9.359639446408 9.333339431833

H 11.882892273778 7.816783143346 8.956038956943

C 3.233371005525 9.480536190972 12.920245474501

H 3.211748232224 10.431649673833 12.365438197185

H 3.742645109746 9.655715636763 13.879028885372

H 2.193625759764 9.185486518038 13.134456982075

C 8.894615120768 8.000925510934 8.217592378873

H 9.862833657065 7.572299536201 7.955592214801

C 7.778039507562 12.668580065859 12.100590071598

H 7.255966201888 12.417040121823 13.035879935871

H 7.043012011775 13.104279743844 11.405068295461

H 8.532439287636 13.439527932472 12.326916029386

C 3.247751314852 8.204883902341 10.755551551088

H 2.189392724741 7.924621968645 10.875252321214

H 3.740295523110 7.430110312326 10.148576107783

H 3.269896025613 9.147995635490 10.188898583598

C 9.079228813577 11.760824811524 10.146911045920

H 9.844500773320 12.548203130709 10.235946029140

H 8.314041525573 12.126630316600 9.445244058902

H 9.548998692174 10.875662217195 9.693978678408

C 5.550588999200 6.731637995213 17.105060441823

H 5.319254913905 7.756499434334 16.781000254282

H 6.299531231635 6.789669683826 17.910833007621

H 4.632975530317 6.286478999086 17.522386691219

C 6.780227520963 8.931863595654 7.497243926307

H 6.116306059283 9.264175485072 6.695725349803

C 8.046364636222 8.426676542327 7.199240952081

H 8.375419085129 8.350056069010 6.159934317891

Structure 14 (1.9 Å) of the scan for the reaction of MII with DippCDI

N 7.939732355042 9.397134196598 13.539890151241

N 7.730200928993 6.953901772689 12.521159452682

H 6.891600345335 6.801853428231 11.960884594419

N 5.968816704856 8.161573917025 13.951017137216

N 10.092139600612 7.091065344498 12.129404519906

N 10.945728669248 7.379367956669 11.161405706198

N 10.209868070852 7.626833389930 10.121821085151

C 7.141870395027 8.427632912561 13.566139661193

C 8.769029981563 7.172795727212 11.723252890145

C 9.184676511055 9.760309970605 13.987961212879

C 5.151504820125 7.078655151625 13.653910504207

C 10.036393925043 10.490552639733 13.114920015542

C 8.878182481869 7.536173923160 10.365239358014

C 9.590316841407 9.519330269004 15.329782939980

C 5.318008220211 5.837888495725 14.318211720937

C 10.619565760576 6.718707574825 13.395741142368

C 8.635789811839 8.845481466957 16.293554792742

H 8.152487687040 8.017418365387 15.750601166700

C 10.001959595713 5.711560205651 14.138834689771

H 9.081938919262 5.256652467661 13.775295316319

C 11.272820971078 10.936592078055 13.586074072755

H 11.932823935650 11.491504015955 12.915695142460

C 11.791653479334 7.329366397428 13.838528207589

H 12.236741237698 8.135068558330 13.254691378328

C 11.679766982381 10.690986959840 14.895315492801

H 12.651725079929 11.045631060876 15.247432214440

C 10.835103468207 9.989479928725 15.751738895935

H 11.154448820456 9.802946167341 16.779032026177

C 7.821485053553 7.871663815834 9.409525209537

C 4.084427035018 7.263502441218 12.739049271946

C 6.868953908110 8.838831861405 9.767267511650

H 6.929322856623 9.318094427700 10.747264460389

C 6.392813674533 5.684941850950 15.374066322167

H 7.258581045689 6.267050605005 15.025328470029

C 11.765755039414 5.899337122736 15.786091881163

H 12.215666723097 5.576063719906 16.727657638281

C 12.364778610366 6.910041916190 15.035304731569

H 13.279442009510 7.390985378458 15.388696440495

C 9.561156125821 10.802072681479 11.712287379753

H 9.032359977054 9.901354743632 11.362491422671

C 10.583430270639 5.308235152732 15.337870146851

H 10.108855516535 4.517430695866 15.922878255265

C 3.864416757976 8.627575135552 12.114347044249

H 4.860626816512 9.023560868336 11.856734002034

C 4.434699693193 4.796073072715 14.023843123002

H 4.556736308515 3.832884966810 14.523801405358

C 3.229234000760 6.189595145493 12.475272327354

H 2.410625811560 6.315291491654 11.763944645463

C 9.307053559724 8.243033379576 17.521104528642

H 10.121623276376 7.558550852081 17.243230453273

H 8.570320470064 7.675130256947 18.109562356196

H 9.722606354366 9.019442599506 18.183293523168

C 7.534303875414 9.825265524605 16.709965352269

H 7.969517855312 10.675537472589 17.259934872165

H 6.800265550733 9.333416754026 17.367333678336

H 6.998929557322 10.216782337856 15.833378657252

C 3.400786618542 4.960183735239 13.105470591066

H 2.724823848604 4.130181032758 12.884429706576

C 5.871405960314 9.204871236692 8.868170743040

H 5.143382428293 9.967545537124 9.151190673525

C 6.864340587427 4.250891526464 15.577881465363

H 6.076192592916 3.612707326330 16.008290378250

H 7.712964858117 4.230747612598 16.278659017674

H 7.190896985389 3.791761471785 14.631608313090

C 10.857592585950 7.971330172196 8.871648811269

H 11.872510970034 8.309856265066 9.104296884899

H 10.289943298741 8.773425348521 8.385443865880

H 10.894968213841 7.091348294932 8.216234456989

C 3.249797091723 9.585872738604 13.139052566091

H 3.148767537307 10.598420739973 12.716506594574

H 3.876988416173 9.649131039730 14.039511255044

H 2.246680630176 9.239357617189 13.436690971998

C 7.747533315748 7.266925012997 8.146342835031

H 8.469639129088 6.495993714401 7.868156991913

C 8.534403777888 11.937422348309 11.724675842389

H 7.703163950987 11.705657264379 12.405924213407

H 8.117771922061 12.100389654361 10.717412151302

H 9.002742048273 12.878493511525 12.056426876209

C 3.029959356346 8.598410887096 10.840458073753

H 1.989051375450 8.298816202211 11.042237715220

H 3.445240940056 7.905188043664 10.093240296878

H 2.996006285001 9.601827428594 10.389264379306

C 10.681046161129 11.099257166118 10.723132773274

H 11.175034224181 12.059940872869 10.940414238576

H 10.275758295241 11.166364174173 9.701461967609

H 11.453976064249 10.315726524529 10.734519211938

C 5.916605196945 6.296675027948 16.693279661414

H 5.626765854632 7.348437044386 16.556256367742

H 6.711962108110 6.257804183554 17.454491540360

H 5.043915419282 5.747344252827 17.082317126162

C 5.807947029680 8.605678812725 7.609512843614

H 5.024373511366 8.894976234060 6.905019444181

C 6.745096397265 7.635738040971 7.252093985583

H 6.693792391141 7.158541094847 6.270611009431

Structure 19 (1.4 Å) of the scan for the reaction of MII with DippCDI

N 7.793535220858 9.567594682389 13.390427093847

N 7.849422548639 7.270111777667 12.930931309147

H 7.299386800571 6.411316364705 12.899439105998

N 5.908985232885 8.256199873260 13.746352521439

N 10.170732184011 7.025715143674 12.297046561285

N 10.960867226693 7.205965397806 11.276768371521

N 10.196599606987 7.643629783776 10.316773878986

C 7.163966417045 8.398769827506 13.395991419075

C 8.866057493473 7.354343314804 12.016680386070

C 9.102314877710 9.788929338225 13.760685650296

C 5.181057106147 7.093212230748 13.528935344755

C 9.975092455029 10.532127548314 12.906642797770

C 8.886150522024 7.738950191837 10.679441958273

C 9.583073996467 9.434675520817 15.060870962294

C 5.389172843035 5.904731790187 14.288740707299

C 10.714067370840 6.511336921106 13.511655058601

C 8.631876648764 8.833010740978 16.075907707573

H 7.987794548989 8.117658868420 15.548150501810

C 10.057653137741 5.473798226560 14.171666335001

H 9.128992390323 5.062518426464 13.776675318130

C 11.272545027050 10.835276674503 13.325739318323

H 11.932241842633 11.392132629085 12.656326425436

C 11.919143072559 7.032403272747 13.975333002806

H 12.391156769788 7.862053250177 13.448362357714

C 11.744483941776 10.451245298724 14.578571815425

H 12.764762038035 10.692285080136 14.887845717650

C 10.886688590043 9.765997188667 15.434272818693

H 11.242766451626 9.483748146636 16.427526112331

C 7.806518303501 8.128015310233 9.766165702357

C 4.114385865151 7.124624180711 12.584404338535

C 6.886923789313 9.120371619976 10.134144581980

H 6.964976011905 9.588649221904 11.119414985039

C 6.350574250117 5.906240874090 15.464003800057

H 7.244945194877 6.473612196416 15.170409750250

C 11.839094126099 5.456970541986 15.805692289152

H 12.282782286982 5.041336726670 16.713264846568

C 12.482209468737 6.493154088116 15.127775744131

H 13.423308168934 6.898279605707 15.505639194255

C 9.461008403752 11.025911495133 11.572069231048

H 8.861373227576 10.205749197178 11.155856153241

C 10.628576812412 4.951979846215 15.329649401385

H 10.123964945989 4.139132332440 15.855793651444

C 3.779217196758 8.431024528409 11.894222347623

H 4.738602220456 8.925281625696 11.682791272619

C 4.605621290884 4.774773775258 14.022954340264

H 4.773627510236 3.860971211982 14.596729432115

C 3.357098895396 5.974220285562 12.359337619156

H 2.551669420525 5.997203357423 11.621956116944

C 9.312313795119 8.067347877555 17.203283936756

H 9.998579341200 7.300529965142 16.815036051543

H 8.557187256846 7.563853391093 17.826440030685

H 9.886975601364 8.732734824717 17.867473348895

C 7.712595393659 9.923499928701 16.631700285532

H 8.298698428429 10.681031010120 17.177718384434

H 6.971249614691 9.498022711866 17.326750674768

H 7.171676259371 10.422936850975 15.814993604788

C 3.605923088641 4.793754575972 13.057065800369

H 3.010566036806 3.898545359289 12.859851901597

C 5.900194224101 9.515882461135 9.235167291295

H 5.196646299443 10.300199920808 9.521725199995

C 6.831534356665 4.525781262253 15.893822884739

H 6.019711017715 3.920048366336 16.326946610146

H 7.609616774366 4.626077331984 16.666183286899

H 7.260388441441 3.960221958899 15.051533599599

C 10.801403873238 7.959190874625 9.034952441148

H 11.843954949312 8.237883315266 9.221120022948

H 10.256885771544 8.797741571875 8.587017724032

H 10.758170709300 7.084516944034 8.373323076544

C 3.010934506052 9.346515191169 12.851820618132

H 2.810547022430 10.324130635198 12.383860600354

H 3.592706995574 9.516611317741 13.768855632370

H 2.043011486845 8.896834273528 13.128056516381

C 7.697852506401 7.511026545730 8.510195678055

H 8.385779781660 6.710751792954 8.229587503159

C 8.507798030171 12.208799179534 11.756240870903

H 7.698279399370 11.942849941942 12.450315722984

H 8.056784289404 12.505591596567 10.795035691658

H 9.046954043760 13.080070708626 12.163133086578

C 3.027582893731 8.269228432422 10.579730436661

H 2.014817095699 7.862610678379 10.733135403336

H 3.559654433991 7.602854795741 9.883476382769

H 2.911636400866 9.246881554110 10.087190216336

C 10.546977057656 11.361152397374 10.557510020825

H 11.111925300455 12.263290351325 10.842837969539

H 10.096480587246 11.558133530636 9.571990855066

H 11.270904545196 10.539503666642 10.444301937567

C 5.708495487068 6.649698033418 16.637842992710

H 5.422763842817 7.668714605344 16.340327008045

H 6.404493579979 6.722193747405 17.488375901078

H 4.804187206117 6.120515118032 16.979504239206

C 5.804600958013 8.913419800408 7.981528365024

H 5.025483253891 9.226388421808 7.282278595583

C 6.697921977460 7.902561800419 7.624891280882

H 6.616803045404 7.414081654772 6.651231401613

**8. References**

[1] a) R. Rudolf, N. I. Neuman, R. R. M. Walter, M. R. Ringenberg, B. Sarkar, *Angew. Chem. Int. Ed.* **2022**, *61*, e202200653. b) A. R. Ali, H. Ghosh, B. K. Patel, *Tetrahedron Lett*. **2010**, *51*, 1019. c) J. Tönnemann, J. Risse, Z. Grote, R. Scopelliti, K. Severin, *Eur. J. Inorg. Chem*. **2013**, 4558.

[2] G. R. Fulmer, A. J. M. Miller, N. H. Sherden, H. E. Gottlieb, A. Nudelman, B. M. Stoltz, J. E. Bercaw, K. I. Goldberg, *Organometallics* **2010**, *29*, 2176.

[3] a) J. Klein, A. Stuckmann, S. Sobottka, L. Suntrup, M. van der Meer, P. Hommes, H.-U. Reissig, B. Sarkar, *Chem. Eur. J.* **2017**, *23*, 12314; b) M. Krejčik, M. Daněk, M., F. Hartl, J*. Electroanal. Chem. Interf. Electrochem.* **1991**, *317*, 179.

[4] a) A. L. Spek, *J. Appl. Crystallogr*. **2003**, *36*, 7; b) G. M. Sheldrick, *Acta Crystallogr., Sect. A: Found. Crystallogr*. **2008**, *64*, 112.

[5] a) G. M. Sheldrick, SHELXS-97, Program for Crystal Structure Solution and Refinement, University of Göttingen, Göttingen, Germany, 1997; b) G. M. Sheldrick, SHELXL Version 2014/7, Program for Chrystal Structure Solution and Refinement, University of Göttingen, Germany, 2014; c) G. M. Sheldrick, SADABS Ver. 2008/1, SADABS. Program for Empirical Absorption Correction, University of Gottingen, Germany, 2012.

[6] a) G. M. Sheldrick, *Acta Crystallogr. A* **2008**, *64*, 112; b) G. M. Sheldrick, *Acta Crystallogr. C* **2015**, *71*, 3; c) G. M. Sheldrick, *Acta Crystallogr. A* **2015**, *71*, 3. d) SAINT+, Data Integration Engine, Version 8.27b©, Bruker AXS Inc., Madison, Wisconsin, USA, 1997-2012.

[7] a) F. Neese, *Wiley Interdiscip. Rev.: Comput. Mol. Sci.* **2012**, *2*, 73; b) F. Neese, *Wiley Interdiscip. Rev.: Comput. Mol. Sci.* **2022**, *12*, e1606.

[8] C. Adamo, V. Barone, *J. Chem. Phys.* **1999**, *110*, 6158.

[9] F. Weigend, R. Ahlrichs, *PCCP* **2005**, *7*, 3297.

[10] J. D. Rolfes, F. Neese, D. A. Pantazis, *J. Comput. Chem.* **2020**, *41*, 1842.

[11] A. V. Marenich, C. J. Cramer, D. G. Truhlar, *J. Phys. Chem. B* **2009**, *113*, 6378.

[12] V. Barone, M. Cossi, *J. Phys. Chem. A* **1998**, *102*, 1995.

[13] a) T. Petrenko, S. Kossmann, F. Neese, *J. Chem. Phys.* **2011**, *134*, 54116; b) F. Neese, G. Olbrich, *Chem. Phys. Lett.* **2002**, *362*, 170; c) R. Izsák, F. Neese, *J. Chem. Phys.* **2011**, *135*, 144105; d) J. L. Whitten, *J. Chem. Phys.* **1973**, *58*, 4496; e) O. Vahtras, J. Almlöf, M.W. Feyereisen, *Chem. Phys. Lett.* **1993**, *213*, 514; f) F. Neese, F. Wennmohs, A. Hansen, U. Becker, *Chem. Phys.* **2009**, *356*, 98.

[14] a) K. Eichkorn, O. Treutler, H. Öhm, M. Häser, R. Ahlrichs, *Chem. Phys. Lett.* **1995**, *242*, 652; b) K. Eichkorn, F. Weigend, O. Treutler, R. Ahlrichs, *Theor. Chem. Acc.* **1997**, *97*, 119; c) F. Weigend, *PCCP* **2006**, *8*, 1057.

[15] S. Grimme, J. Antony, S. Ehrlich, H. Krieg, *J. Chem. Phys.* **2010**, *132*, 154104.

[16] "Chemcraft - graphical software for visualization of quantum chemistry computations. https://www.chemcraftprog.com".

[17] M. J. Frisch, G. W. Trucks, H. B. Schlegel, G. E. Scuseria, M. A. Robb, J. R. Cheeseman, G. Scalmani, V. Barone, B. Mennucci, G. A. Petersson, H. Nakatsuji, M. Caricato, X. Li, H. P. Hratchian, A. F. Izmaylov, J. Bloino, G. Zheng, J. L. Sonnenberg, M. Hada, M. Ehara, K. Toyota, R. Fukuda, J. Hasegawa, M. Ishida, T. Nakajima, Y. Honda, O. Kitao, H. Nakai, T. Vreven, J. A. Montgomery, Jr. J. E. Peralta, F. Ogliaro, M. Bearpark, J. J. Heyd, E. Brothers, K. N. Kudin, V. N. Staroverov, T. Keith, R. Kobayashi, J. Normand, K. Raghavachari, A. Rendell, J. C. Burant, S. S. Iyengar, J. Tomasi, M. Cossi, N. Rega, J. M. Millam, M. Klene, J. E. Knox, J. B.Cross, V. Bakken, C. Adamo, J. Jaramillo, R. Gomperts, R. E. Stratmann, O. Yazyev, A. J. Austin, R. Cammi, C. Pomelli, J. W.Ochterski, R. L. Martin, K. Morokuma, V. G. Zakrzewski, G. A. Voth, P. Salvador, J. J. Dannenberg, S. Dapprich, A. D. Daniels, O. Farkas, J. B. Foresman, J. V. Ortiz, J. Cioslowski and D. J. Fox, Gaussian 09 (Revision B.01), Gaussian, Inc., Wallingford CT, 2010.

[18] G. Dubey, S. Awari, T. Singh, S. C. Sahoo, P. V. Bharatam, *ChemPlusChem*. **2021**, *86*, 1416.

[19] V. Ásgeirsson, B. O. Birgisson, R. Bjornsson, U. Becker, F. Neese, C. Riplinger, H. Jónsson, *J. Chem. Theory Comput.* **2021**, *17*, 4929.
