# Supplementary material for: Exploring Mesoionic Imine‐Carbodiimide (MII‐CDI) Adducts: 1,3 H‐Shift, N(I) Compounds and Guanidinate‐Type Ligands
Source: Angew Chem Int Ed Engl. 2025 Jul 16;64(34):e202502097. doi: 10.1002/anie.202502097 (PMC12363636; doi:10.1002/anie.202502097)

D:\2025\...\Mahata-AM382-ASAP_positiv Mahata 05/09/25 10:58:59
ASAP positiv am Exactive Plus AM382

Mahata-AM382-ASAP_positiv #461-507 RT: 4.02-5.23 AV: 47 SB: 122 0.28-1.33 NL: 1.10E8
T: FTMS + p ESI Full ms [100.0000-950.0000]

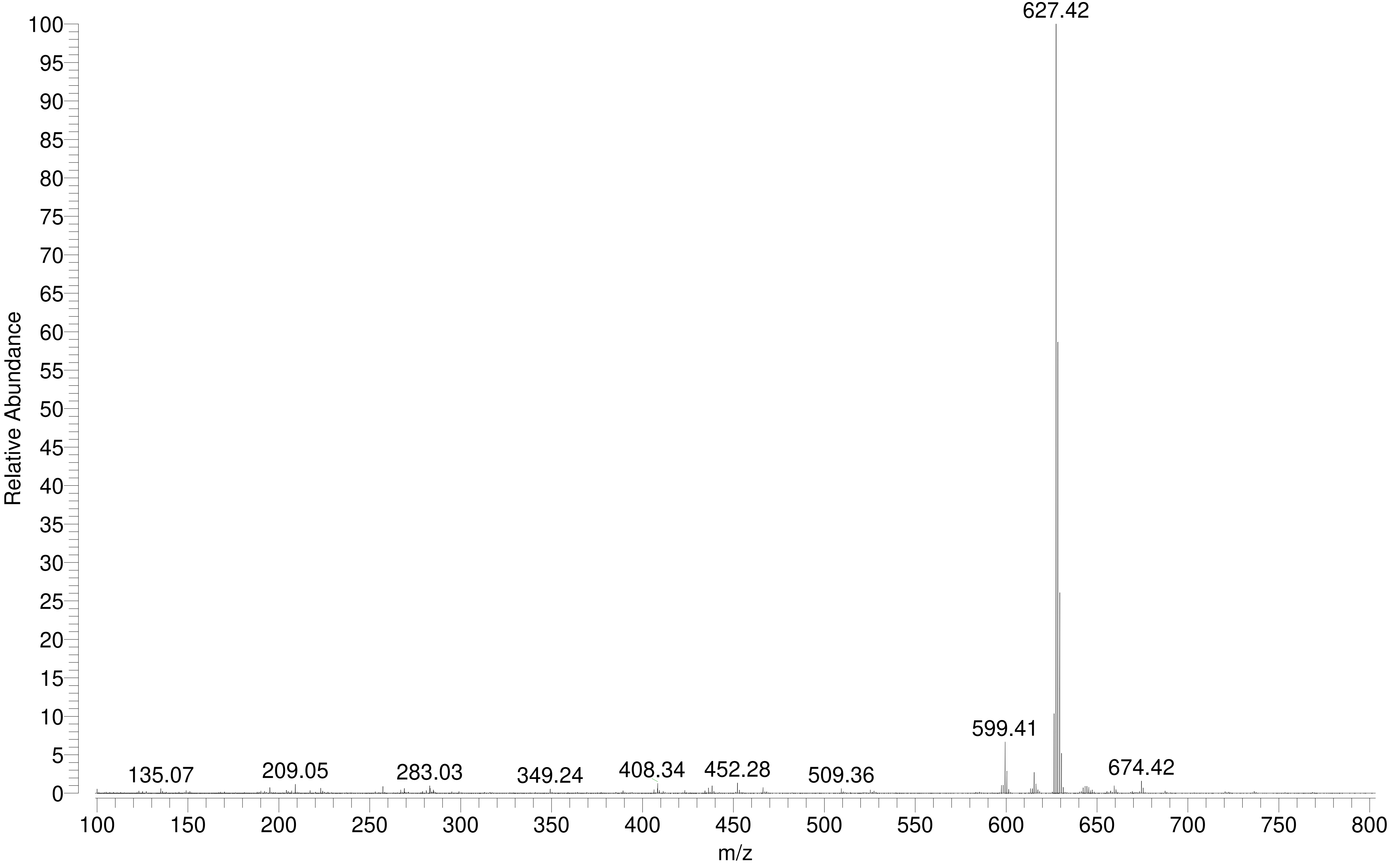

D:\2025\...\Mahata-AM382-ASAP_positiv Mahata 05/09/25 10:58:59
ASAP positiv am Exactive Plus AM382

Mahata-AM382-ASAP_positiv #461-507 RT: 4.02-5.23 AV: 47 SB: 122 0.28-1.33 NL: 1.10E8
T: FTMS + p ESI Full ms [100.0000-950.0000]

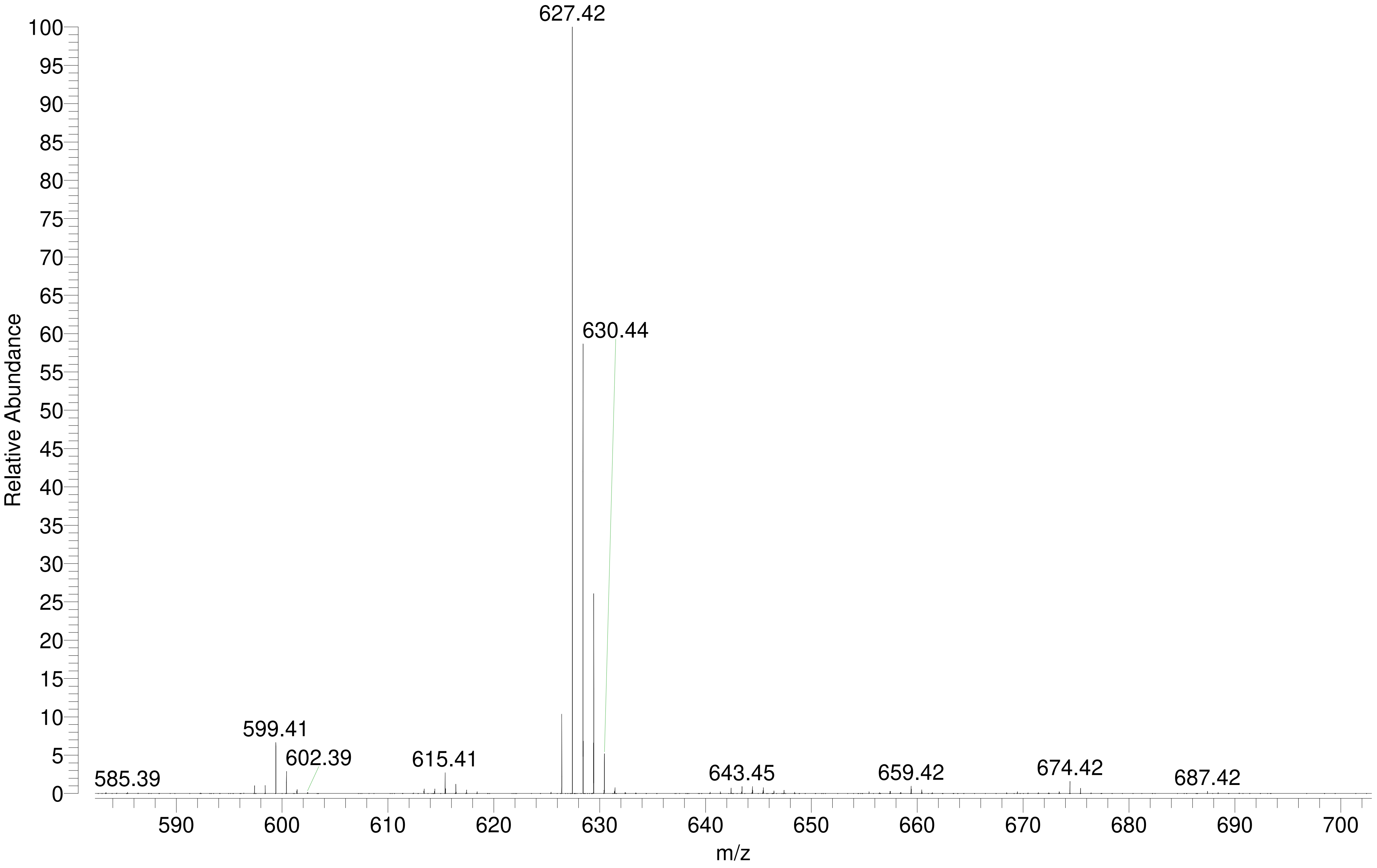

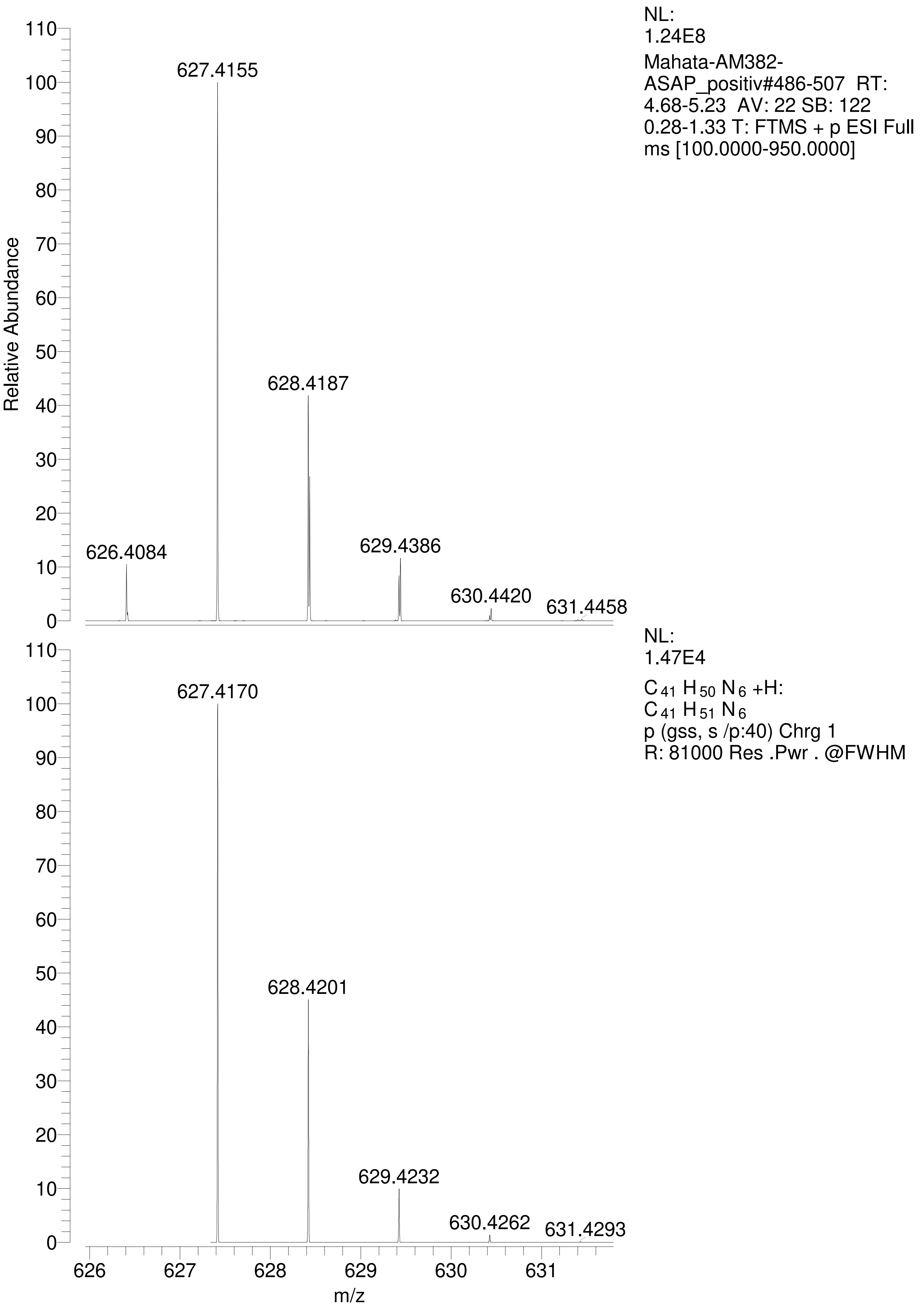
NL:
1.24E8
Mahata-AM382-ASAP_positiv#486-507 RT: 4.68-5.23 AV: 22 SB: 122 0.28-1.33 T: FTMS + p ESI Full ms [100.0000-950.0000]
627.4155
628.4187
626.4084
629.4386
630.4420
631.4458
NL:
1.47E4
C 41 H 50 N 6 +H:
C 41 H 51 N 6
p (gss, s /p:40) Chrg 1
R: 81000 Res .Pwr . @FWHM
627.4170
628.4201
629.4232
630.4262
631.4293
Relative Abundance
m/z

D:\2025\...\Mahata-AM382-ASAP_positiv Mahata 05/09/25 10:58:59
ASAP positiv am Exactive Plus AM382

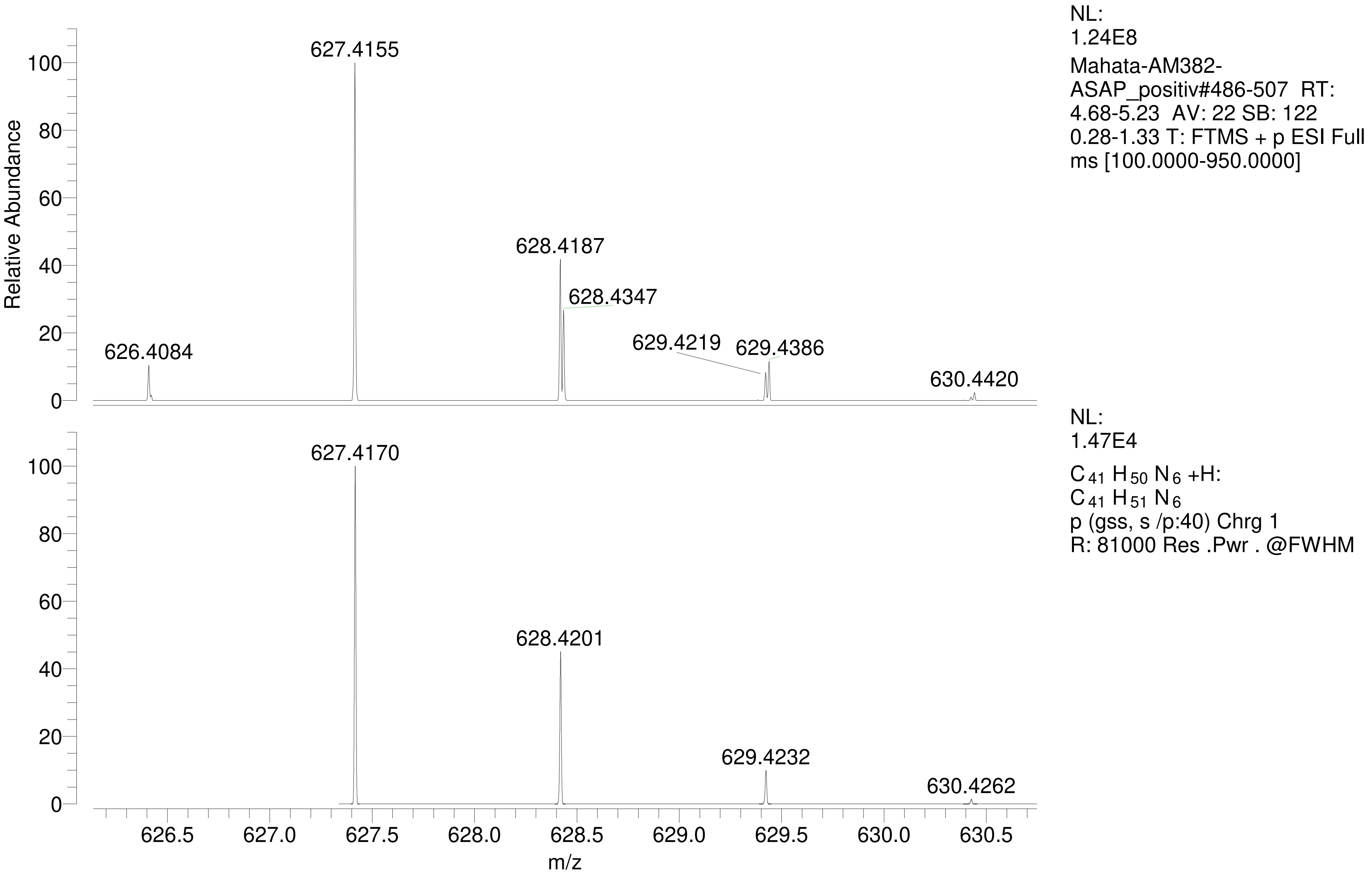

Supplement: Supplementary file 2 — Supporting Information [file ANIE-64-e202502097-s001.zip › Mahata-AM382-ASAP_positiv.pdf]
