# Supplementary material for: Exploring Mesoionic Imine‐Carbodiimide (MII‐CDI) Adducts: 1,3 H‐Shift, N(I) Compounds and Guanidinate‐Type Ligands
Source: Angew Chem Int Ed Engl. 2025 Jul 16;64(34):e202502097. doi: 10.1002/anie.202502097 (PMC12363636; doi:10.1002/anie.202502097)

Mahata-AM382-ESI_positiv_direkt Mahata 05/09/25 10:46:57
ESI positiv am Exactive Plus, direkt AM382

Mahata-AM382-ESI_positiv_direkt #448-596 RT: 4.56-7.35 AV: 149 SB: 78 0.82-1.49 NL: 1.81E8
T: FTMS + p ESI Full ms [100.0000-950.0000]

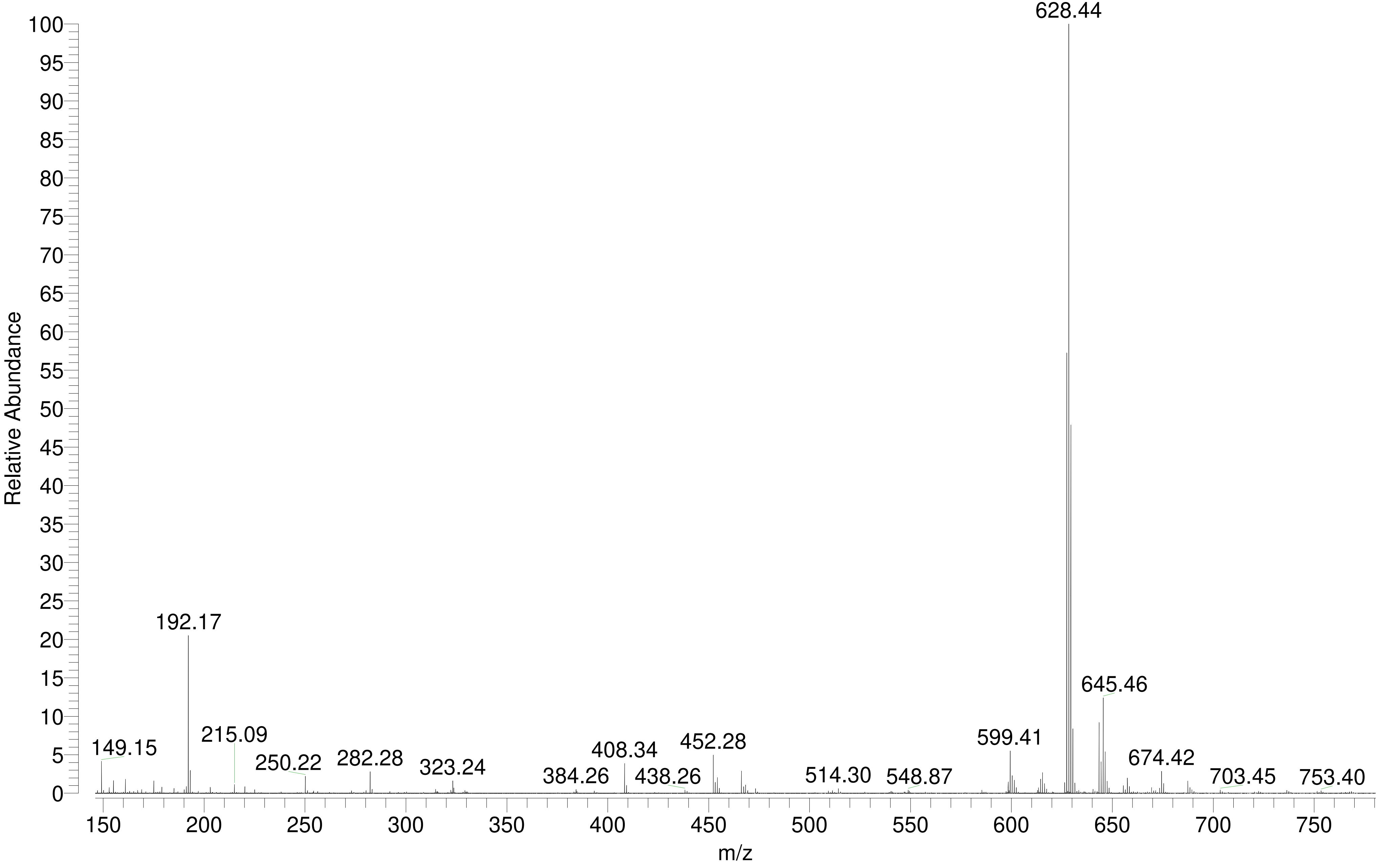

Mahata-AM382-ESI_positiv_direkt
ESI positiv am Exactive Plus, direkt

Mahata
AM382

05/09/25 10:46:57

Mahata-AM382-ESI_positiv_direkt #448-596 RT: 4.56-7.35 AV: 149 SB: 78 0.82-1.49 NL: 1.81E8
T: FTMS + p ESI Full ms [100.0000-950.0000]

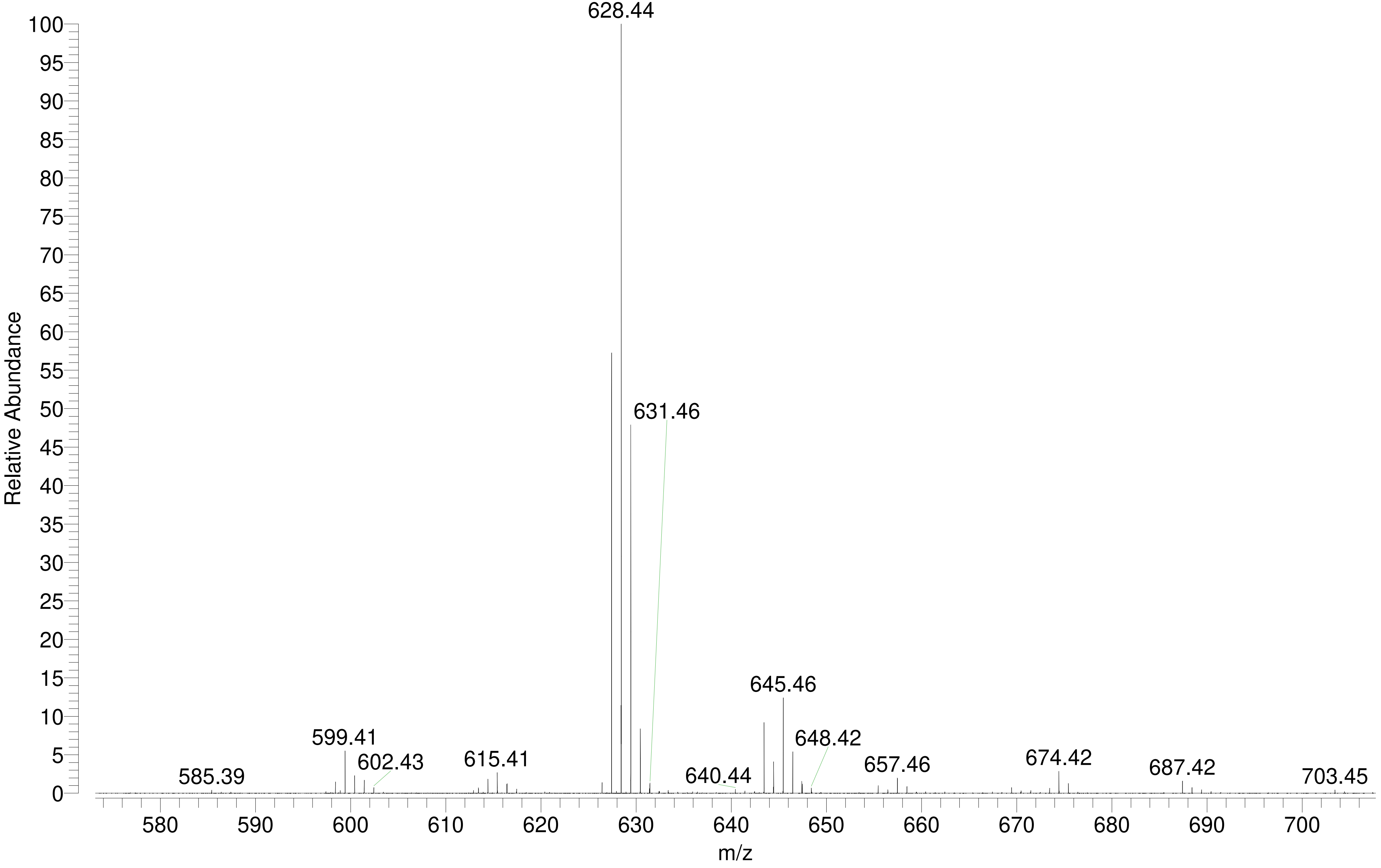

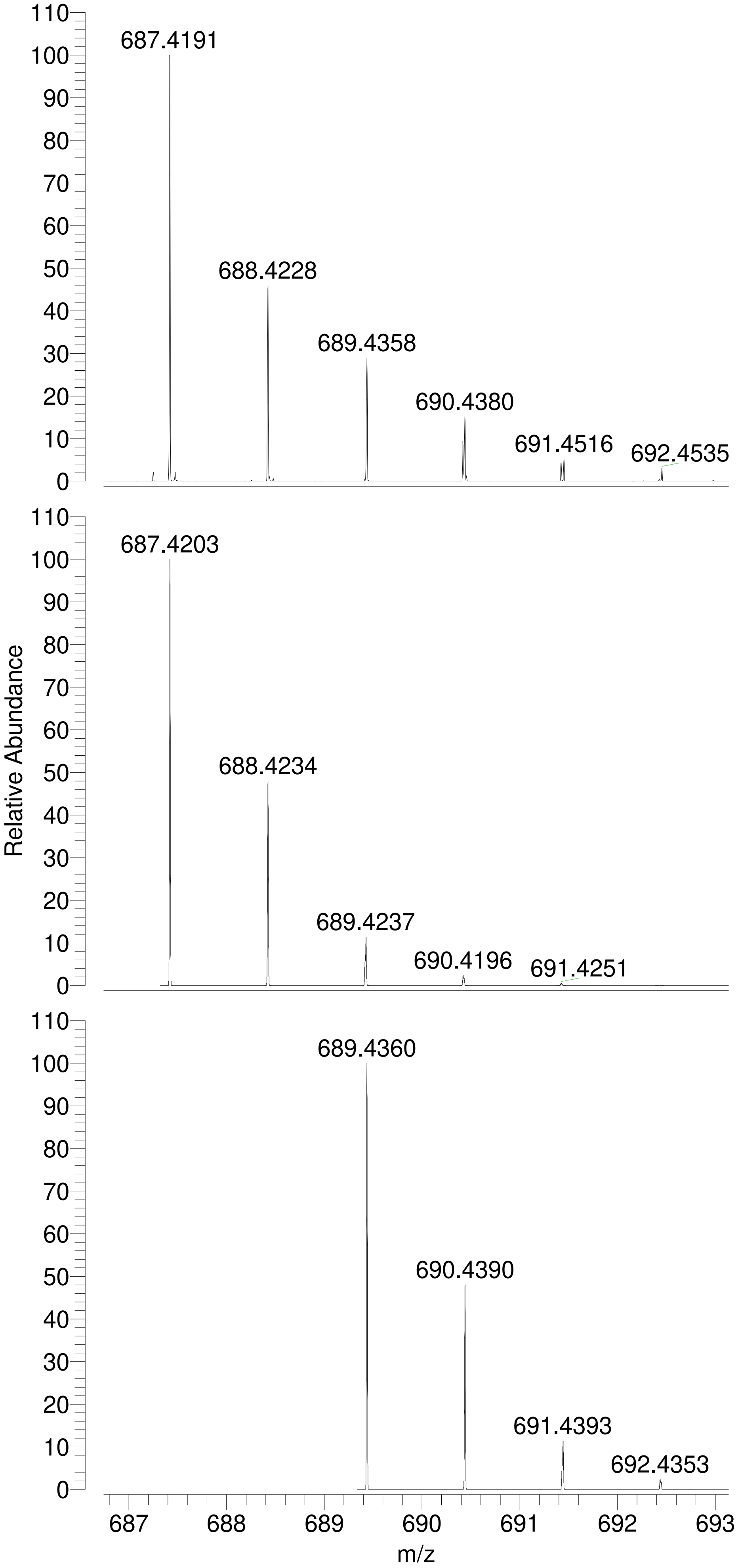

NL:
2.22E6
Mahata-AM382-ESI_positiv_direkt#386-596 RT: 3.46-7.35 AV: 211 SB: 78 0.82-1.49 T: FTMS + p ESI Full ms [100.0000-950.0000]

NL:
1.36E4
$C_{43}H_{55}N_6S_1$:
$C_{43}H_{55}N_6S_1$
p (gss, s /p:40) Chrg 1
R: 76000 Res .Pwr . @FWHM

NL:
1.36E4
$C_{43}H_{57}N_6S_1$:
$C_{43}H_{57}N_6S_1$
p (gss, s /p:40) Chrg 1
R: 76000 Res .Pwr . @FWHM

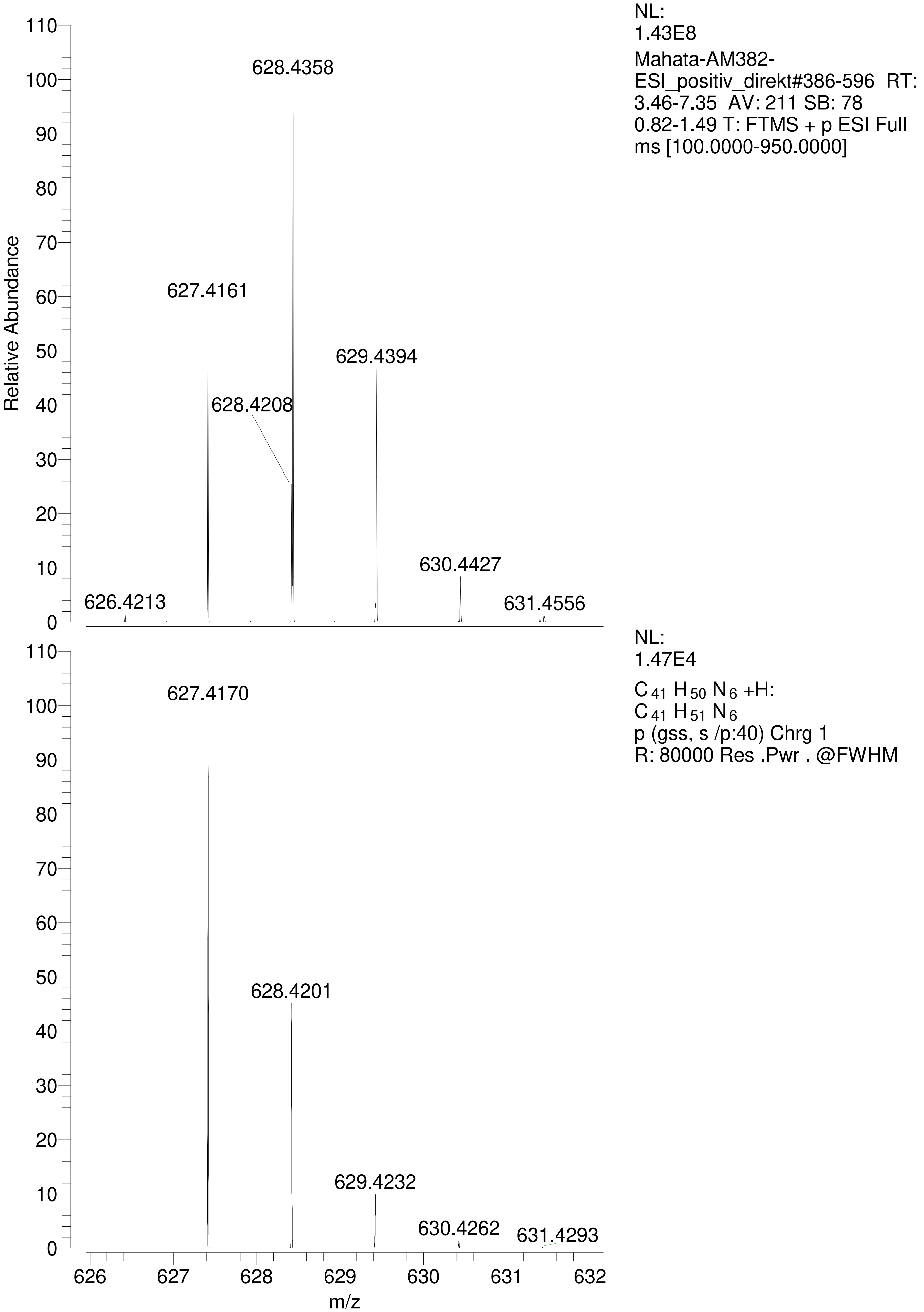
NL:
1.43E8
Mahata-AM382-ESI_positiv_direkt#386-596 RT: 3.46-7.35 AV: 211 SB: 78 0.82-1.49 T: FTMS + p ESI Full ms [100.0000-950.0000]
628.4358
627.4161
629.4394
628.4208
630.4427
626.4213
631.4556
NL:
1.47E4
C 41 H 50 N 6 +H:
C 41 H 51 N 6
p (gss, s /p:40) Chrg 1
R: 80000 Res .Pwr . @FWHM
627.4170
628.4201
629.4232
630.4262
631.4293
Relative Abundance
m/z

Mahata-AM382-ESI_positiv_direkt
ESI positiv am Exactive Plus, direkt

Mahata
AM382

05/09/25 10:46:57

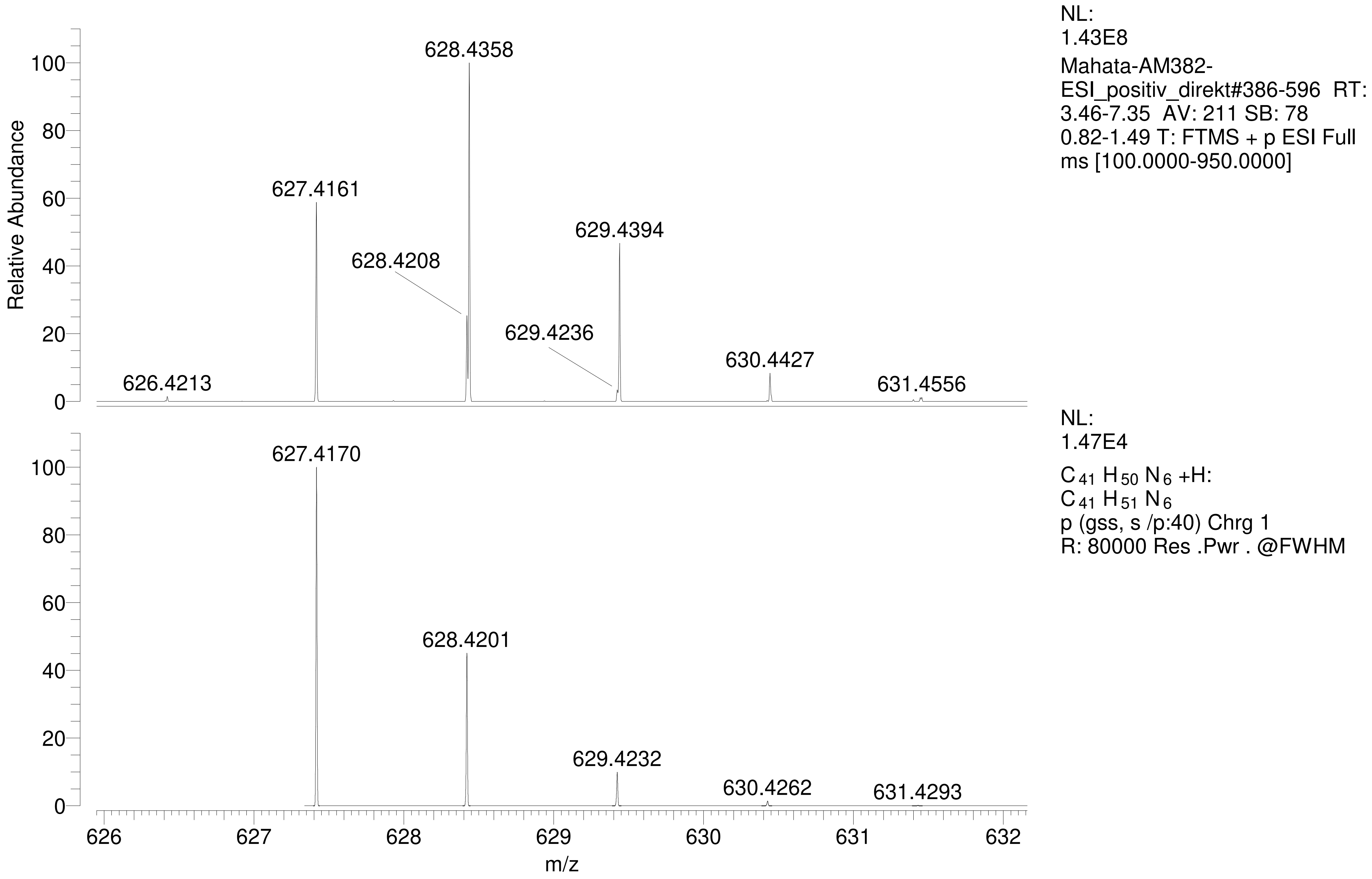

Mahata-AM382-ESI_positiv_direkt | Mahata | 05/09/25 10:46:57
ESI positiv am Exactive Plus, direkt | AM382

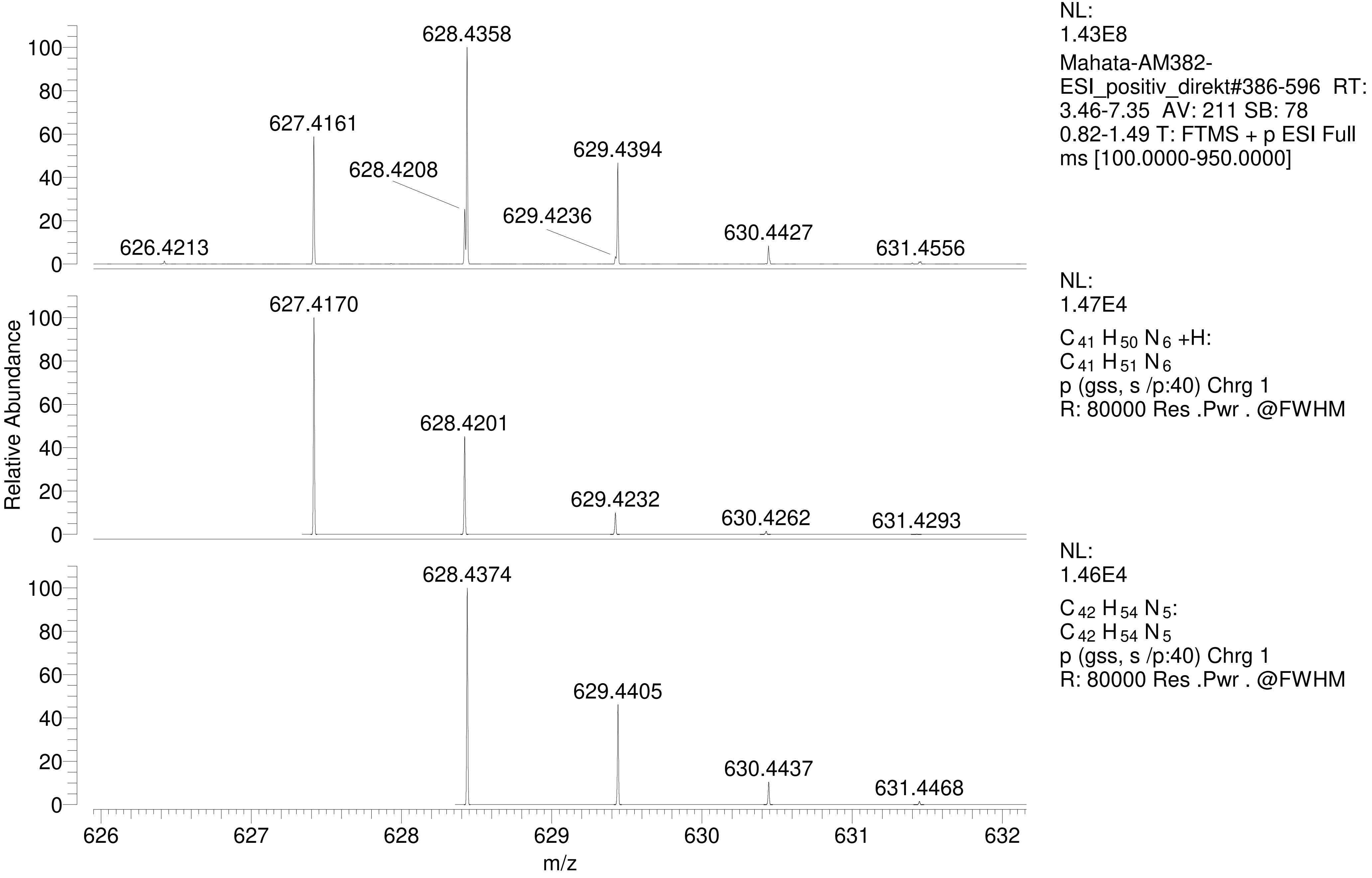

Supplement: Supplementary file 2 — Supporting Information [file ANIE-64-e202502097-s001.zip › Mahata-AM382-ESI_positiv_direkt.pdf]
