# Supplementary figures and images for: Exploring Mesoionic Imine‐Carbodiimide (MII‐CDI) Adducts: 1,3 H‐Shift, N(I) Compounds and Guanidinate‐Type Ligands
Source: Angew Chem Int Ed Engl. 2025 Jul 16;64(34):e202502097. doi: 10.1002/anie.202502097 (PMC12363636; doi:10.1002/anie.202502097)

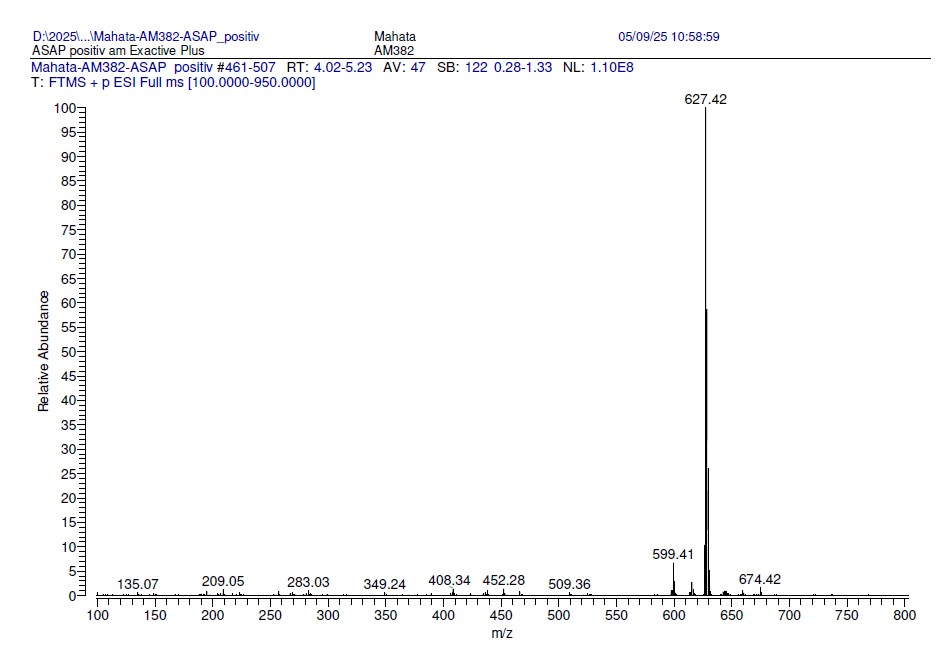


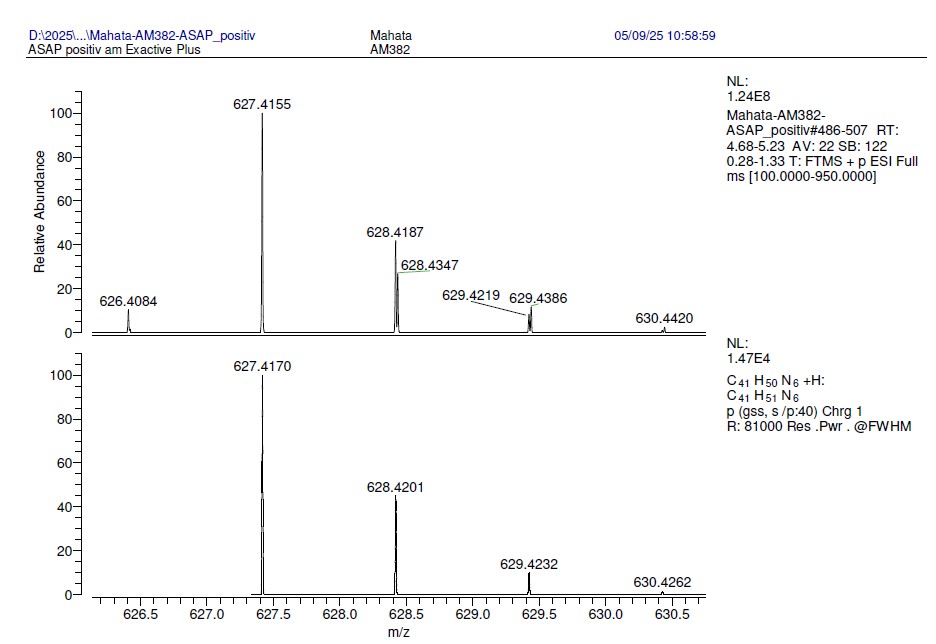


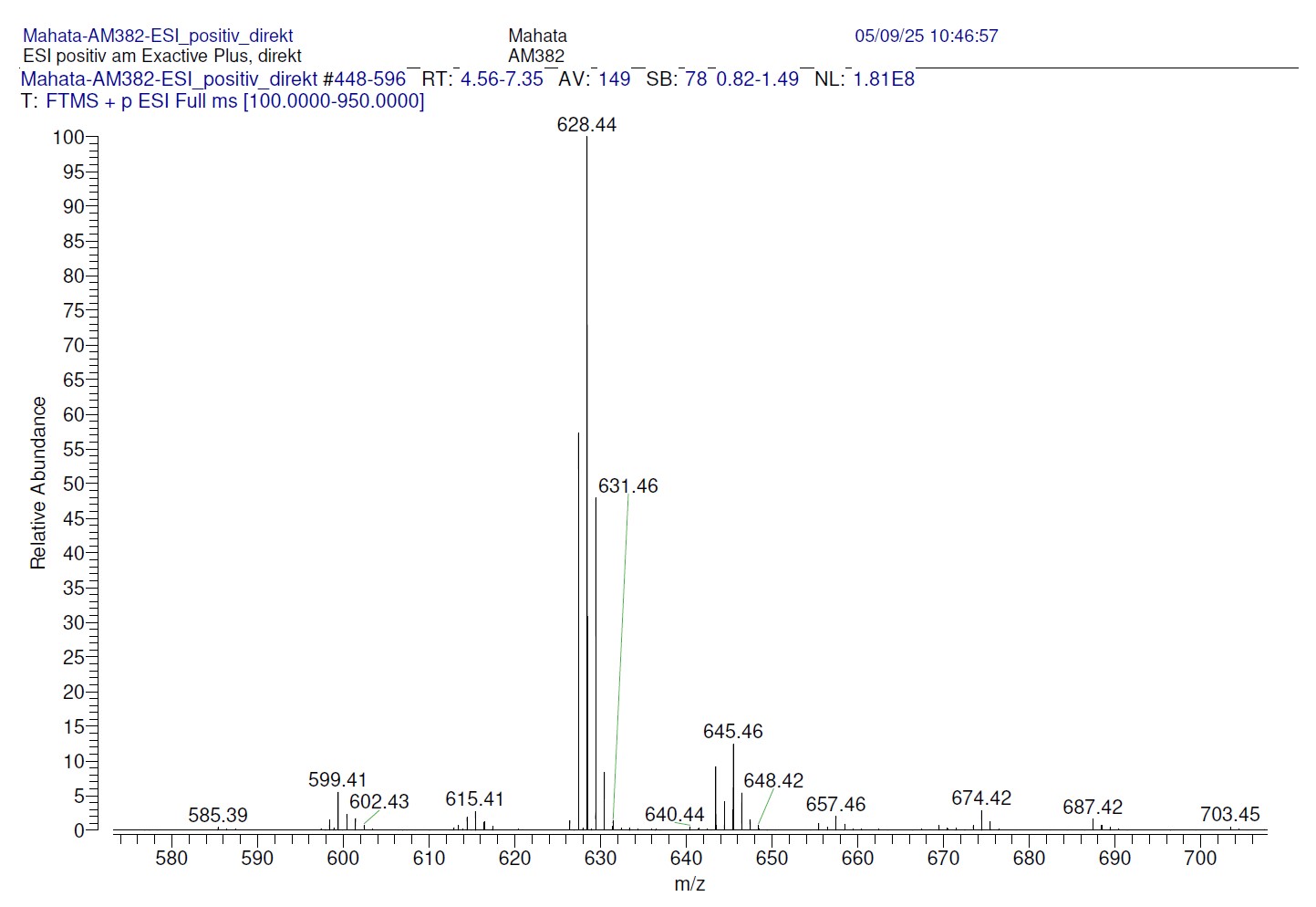


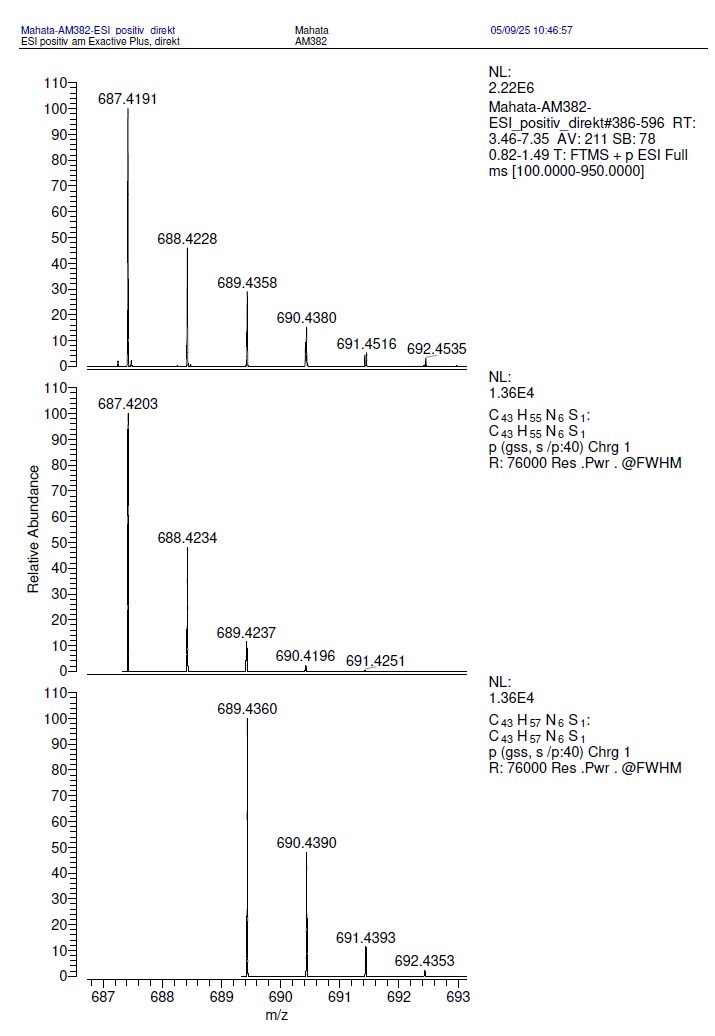

Supplement: Supplementary file 2 — Supporting Information [file ANIE-64-e202502097-s001.zip › HRMS_DippN1_Reduced(1).docx]
